# Supplementary material for: AlphaPeptDeep: a modular deep learning framework to predict peptide properties for proteomics
Source: Nat Commun. 2022 Nov 24;13:7238. doi: 10.1038/s41467-022-34904-3 (PMC9700817; doi:10.1038/s41467-022-34904-3)

Supplementary Figures

AlphaPeptDeep: A modular deep learning framework to predict peptide properties for proteomics

Wen-Feng Zeng[1], Xie-Xuan Zhou[1], Sander Willems[1], Constantin Ammar[1], Maria Wahle[1], Isabell Bludau[1], Eugenia Voytik[1], Maximillian T. Strauss[2], Matthias Mann[1,2,*]

1. Department of Proteomics and Signal Transduction, Max Planck Institute of Biochemistry, Martinsried, Germany
2. Proteomics Program, NNF Center for Protein Research, Faculty of Health Sciences, University of Copenhagen, Copenhagen, Denmark
* E-mail: mmann@biochem.mpg.de

Supplementary Figure 1. Amino acid (AA) and modification embedding. 2
Supplementary Figure 2. An illustrative example of using the model shop module..................... 3
Supplementary Figure 3. Prediction speed of the MS2, RT and CCS models in AlphaPeptDeep. . 4
Supplementary Figure 4. Comparing transformers with LSTM. 5
Supplementary Figure 5. RT model fine-tuning for short LC gradients... 6
Supplementary Figure 6. Visualizing transformer model attention with bertviz. 7
Supplementary Figure 7. Sequence motifs of identified phospho-HLA peptides......................... 8
Supplementary Figure 8. Inspecting of phospho-HLA peptides using MS2 and RT prediction. 9
Supplementary Figure 9. Identified modifications by AlphaPeptDeep with Open-pFind. 10
Supplementary Figure 10. Producer-consumer schema for rescoring with multiprocessing...... 11

Supplementary Figure 1. Amino acid (AA) and modification embedding.

There are two ways to embed AAs in AlphaPeptDeep: (1) one-hot encoding and (2) *torch.Embedding*. As shown in the left panel, one-hot encoding in AlphaPeptDeep generates a one-hot vector that is filled with zeros except for the position indicated by ID of the AA, which is set to one. The one-hot vector is then mapped into an embedded vector (also called tensor) using a linear layer. *torch.Embedding* generates an embedded vector directly from the ID of each AA.

To represent a modification on an AA site, we use its chemical composition. To generate the embedded vector, we fix the number of C, H, N, O, S, and P atoms as the first 6 elements of the vector. For other atoms, we map them into a 2-D vector using a linear layer and concatenate the 2-D vector with the fixed 6 elements. Finally, a vector of size 8 is able to represent a modification.

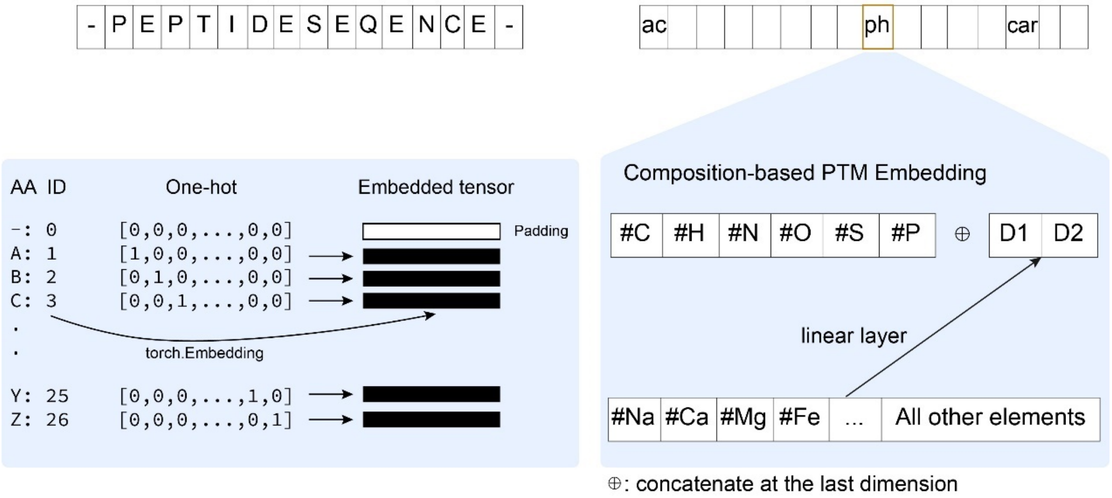

Supplementary Figure 2. An illustrative example of using the model shop module.

We built a deep learning model with only a few lines of code to predict if a peptide elutes in the first half or the second half of the LC gradient (‘is_first_half_lc’). We then called ‘model.train()’ on the training set to train the model and ‘model.predict()’ on the testing set. We used the 351,804 PSMs of tryptic HeLa peptides identified by MaxQuant at 1% FDR measured by a two-hour gradient[1]. ‘is_first_half_lc’ is set as 1 if the PSM is at the first hour, otherwise 0. We then split the PSMs into two sets, where 80% for training and 20% for testing, without any intersections on sequence-level. The training took 16 min, and achieved 95% accuracy on the 70,018 testing PSMs.

```
from peptdeep.model.model_shop import (
    Model_for_Generic_ModAASeq_BinaryClassification_Transformer,
    ModelInterface_for_Generic_ModAASeq_BinaryClassification
)

model = ModelInterface_for_Generic_ModAASeq_BinaryClassification(
    model_class=Model_for_Generic_ModAASeq_BinaryClassification_Transformer
)
model.target_column_to_train = 'is_first_half_lc'
model.target_column_to_predict = 'predicted_is_first_half_lc'
model.train(train_df, epoch=50, warmup_epoch=20, lr=1e-5, verbose=True)
model.predict(test_df)
```

✓ 16m 13.6s

	sequence	mods	mod_sites	rt_norm	is_first_half_lc	predicted_is_first_half_lc
0	DFSHTGR			0.103493	1	0.998056
8	RLPPIVR			0.343450	1	0.940949
13	DYHATWK			0.230220	1	0.997083
14	ISHFTQR			0.127492	1	0.997336
15	ISHFTQR			0.127609	1	0.997336
...	...	...	...	...	...	...
351797	SPVGSGAPQAAAPAPAAHVAGNPGGDAAPAATGTAAAASLATAAGS...			0.644790	0	0.005870
351798	AAAAAAAAAAAATGTEAGPGTAGGSENGSEVAAQPAGLSGPAEVGP...			0.718966	0	0.001388
351799	SPVGSGAPQAAAPAPAAHVAGNPGGDAAPAATGTAAAASLATAAGS...			0.624617	0	0.005870
351800	AAAAAAAAAAAATGTEAGPGTAGGSENGSEVAAQPAGLSGPAEVGP...			0.718805	0	0.001388
351801	SPVGSGAPQAAAPAPAAHVAGNPGGDAAPAATGTAAAASLATAAGS...			0.630824	0	0.005870

70018 rows × 6 columns

Testing accuracy = 95% (pred cutoff=0.5)

Supplementary Figure 3. Prediction speed of the MS2, RT and CCS models in AlphaPeptDeep.

(a) To verify the speed of AlphaPeptDeep, we first compared the time of fragment intensity prediction with Prosit-Transformer on the 1,419,705 test peptides used in Prosit-Transformer paper (https://figshare.com/articles/dataset/LMDB_data_Tape_Input_Files/16688905). On a RTX3080 GPU, AlphaPeptDeep is nearly 40 times faster than Prosit-Transformer, this is because MS2 model of AlphaPeptDeep is much smaller than Prosit-Transformer (4M NN parameters vs 64M NN parameters), thus AlphaPeptDeep can predict many more peptides in parallel. And the other reason may be that AlphaPeptDeep expands the transformer model based on peptide lengths, this much saves the prediction time for short peptides. While Prosit and Prosit-Transformer converts all amino acid sequences into 30-lengh tokens, no matter how short the sequences are.

To further test the prediction speed, we used AlphaPeptDeep to predict the proteome library. Peptides were in silico digested from reviewed E. coli, fission yeast, and human protein sequences by using trypsin with at most 2 missed cleavages, with peptide lengths from 7 to 30. Carbamidomethyl@C was set as the fixed modification. Oxidation@M was set as the variable modification with a maximum occurrence of 2. **(b)** shows the overall peptide and precursor numbers for each species. Precursor charges are from 2 to 4 and m/z are from 200 to 2000. **(c)** shows the prediction time on CPU with multiprocessing. There is no separate time for CCS prediction as CCS and MS2 (including fragment m/z calculation) are predicted together in the multiprocessing mode. **(d)** shows the prediction time on a RTX3080 GPU. Note that even for predicting nearly 8M precursors from human proteome, the total prediction time for RT, CCS, and MS2 was less than 10 minutes. Saving the predicted values into the HDF file took only ~2 minutes. This demonstrates that generating a predicted spectral library is very fast if a GPU is used. When multiprocessing is used, the prediction time is acceptable (~2 hours).

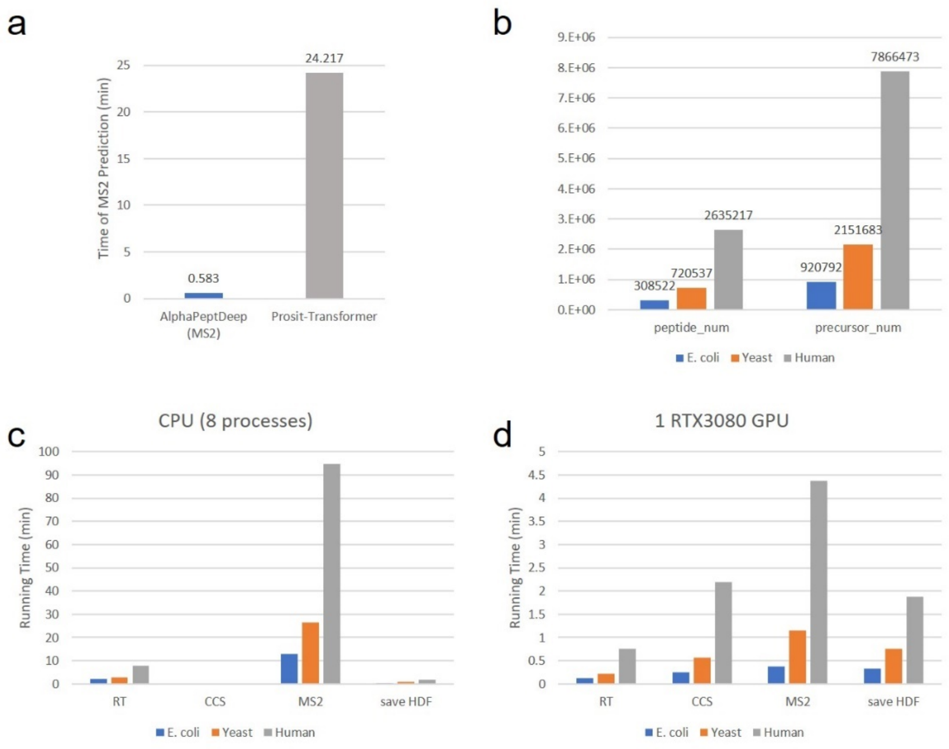

Supplementary Figure 4. Comparing transformers with LSTM.

The transformer and LSTM MS2 prediction models were trained and tested on the same tryptic peptides. The LSTM model is very similar to the pDeep model that we used before. The comparison shows that the transformer is slightly better than LSTM for MS2 prediction.

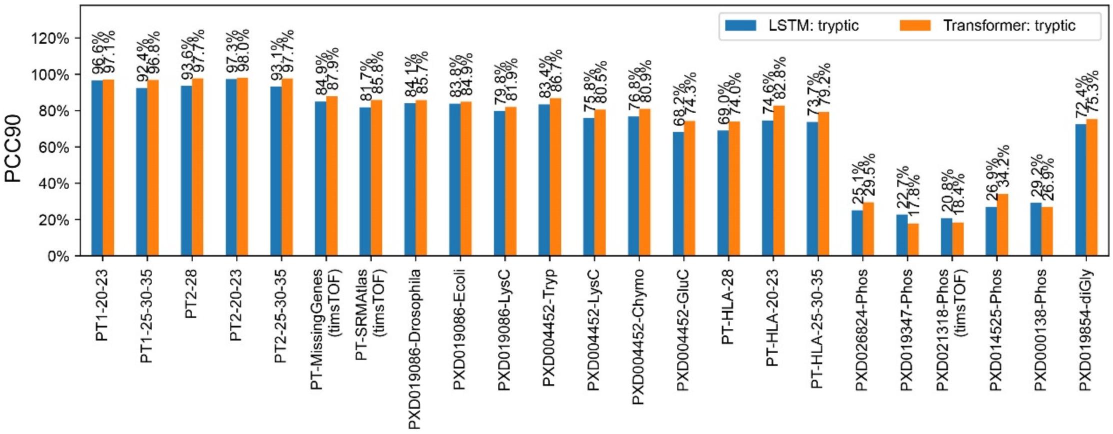

Supplementary Figure 5. RT model fine-tuning for short LC gradients.

Fine-tuning the RT model on **(a)** 30- and **(b)** 15-minute LC gradient. MaxQuant results were downloaded from PXD006932.[2]

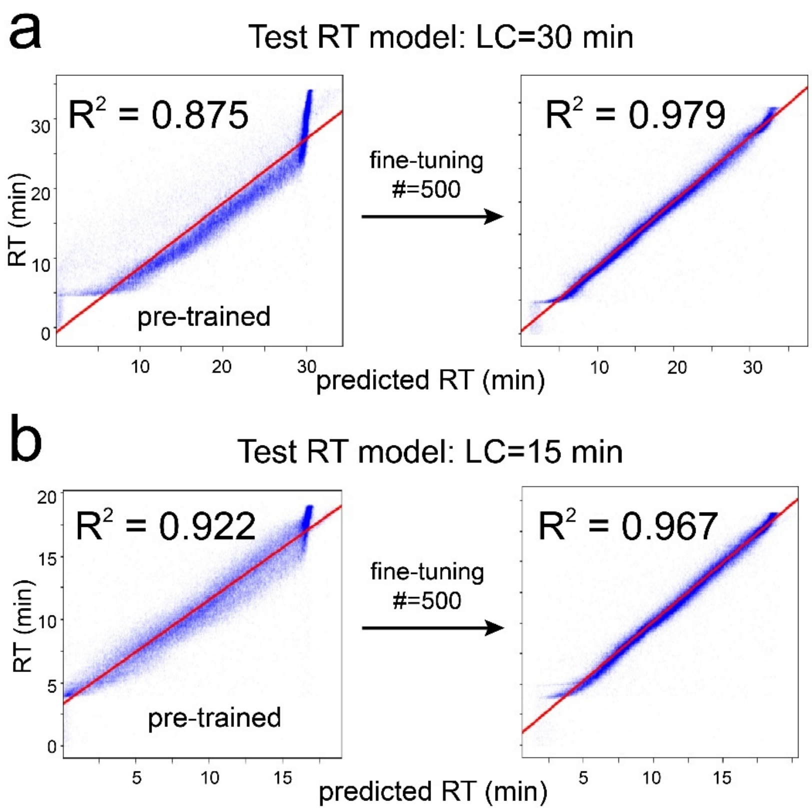

Supplementary Figure 6. Visualizing transformer model attention with bertviz.

The attention values of the 8 heads in the first transformer layer (Layer 0) and the last layer (Layer 3) of the pre-trained and transfer-learned MS2 models are shown. The right panels are the zoom-in plots of head 2 and 3 of these two models.

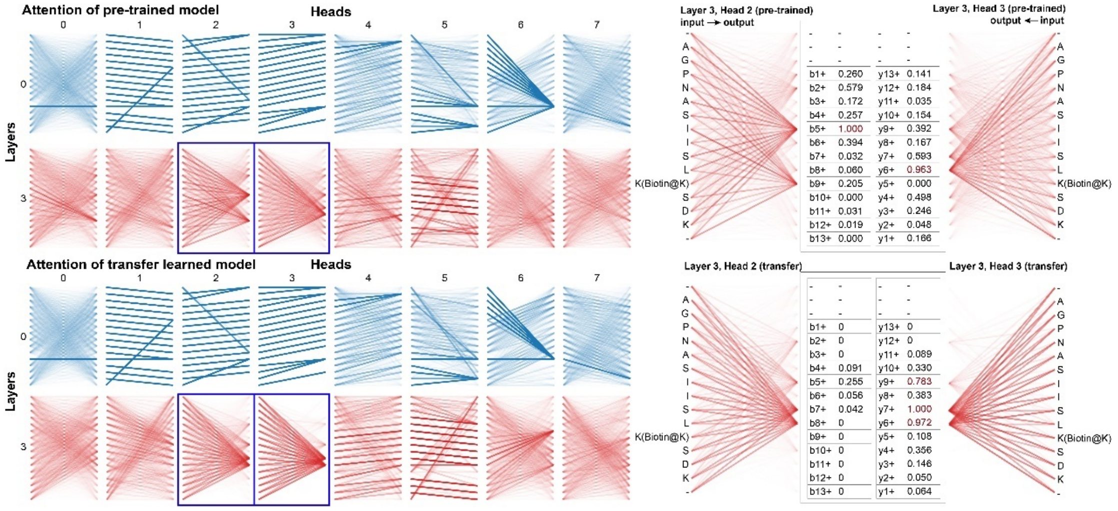

Supplementary Figure 7. Sequence motifs of identified phospho-HLA peptides.

Unmodified and phosphorylated peptides with 9-, 10-, and 11-mers are displayed. The peptides in the first panel were identified on tumor dataset from one of the patients (Mel-15). Peptides from other panels were identified from mono-allelic dataset.

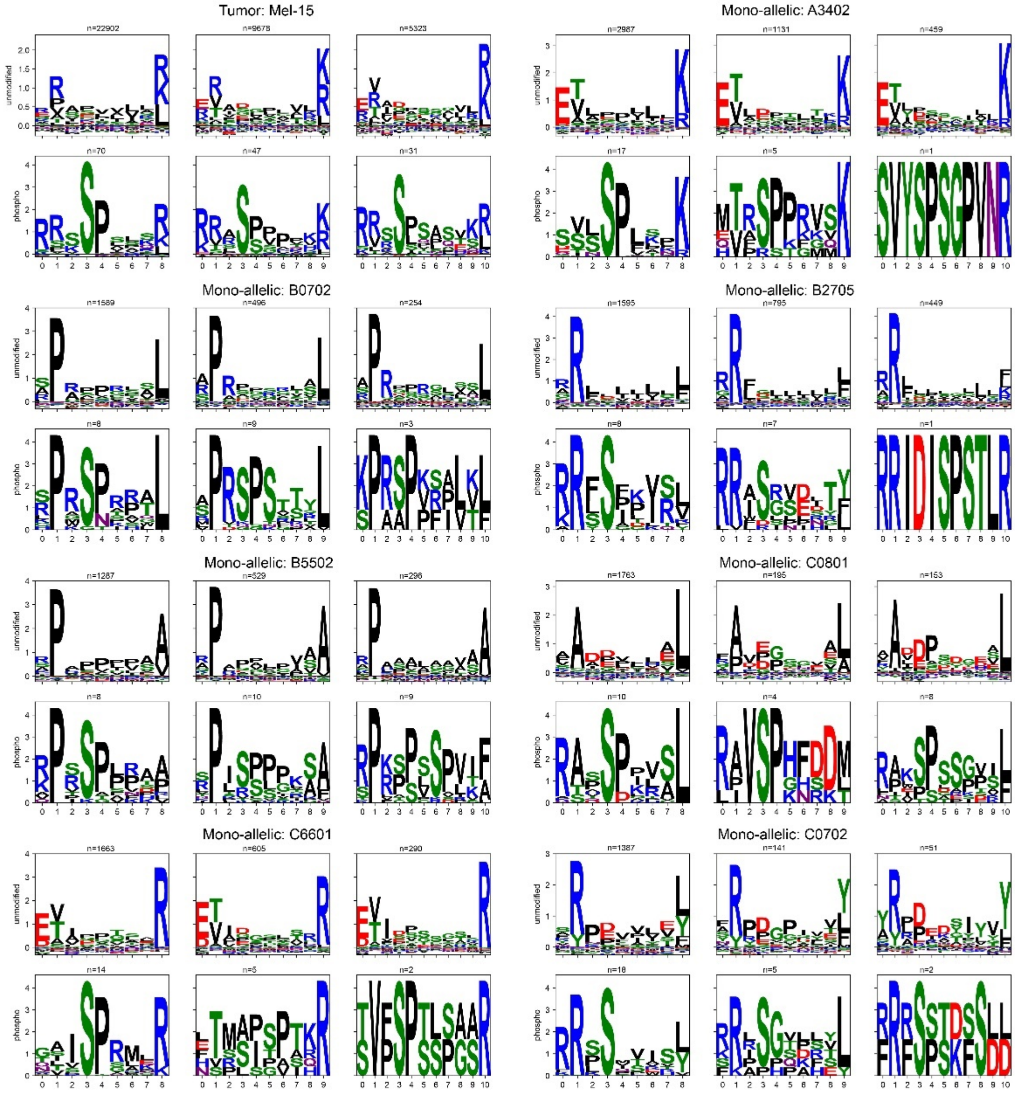

Supplementary Figure 8. Inspecting of phospho-HLA peptides using MS2 and RT prediction.

PCC distribution between experimental and predicted MS2 spectra for unmodified and phospho-HLA peptides on **(a)** the mono-allelic and **(b)** tumor datasets. It shows that PCCs of phospho-HLA peptides are quite close to the unmodified ones. **(c)** The RT difference between detected and predicted RT values on the mono-allelic dataset. Boxplot for n=2650 RT difference values in minutes is drawn with the interquartile range within the boxes and the median as a horizontal line. The whiskers extend to 1.5 times the size of the interquartile range**. (d)** The RT difference between detected and predicted RT values on the tumor dataset. Boxplot for n=1516 RT difference values in minutes is drawn with the interquartile range within the boxes and the median as a horizontal line. The whiskers extend to 1.5 times the size of the interquartile range**.**

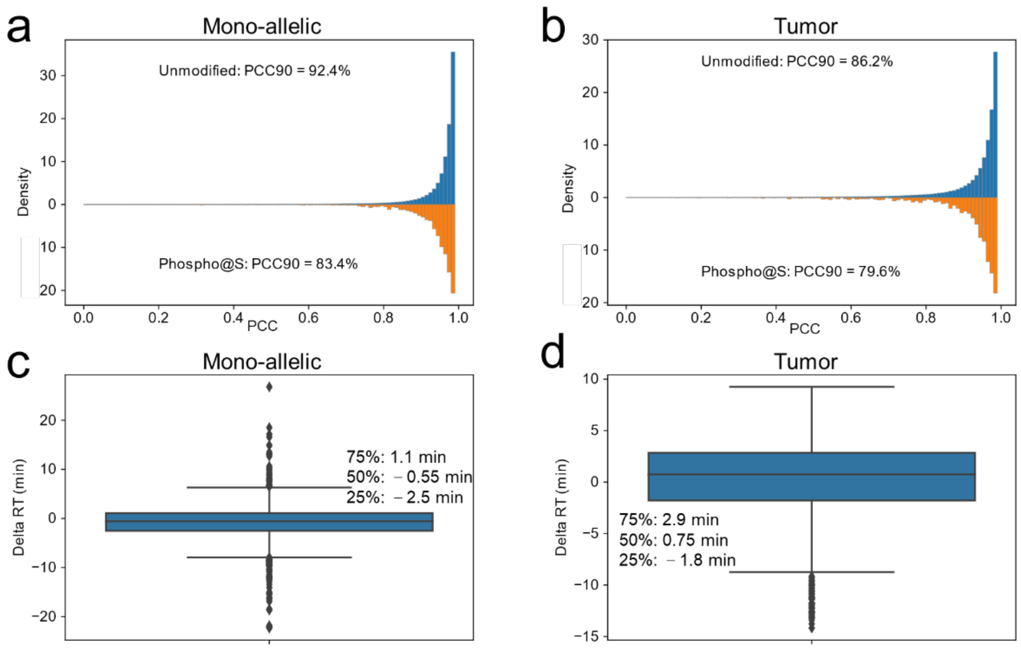

Supplementary Figure 9. Identified modifications by AlphaPeptDeep with Open-pFind.

(a) Mono-allelic dataset. (b) Tumor dataset.

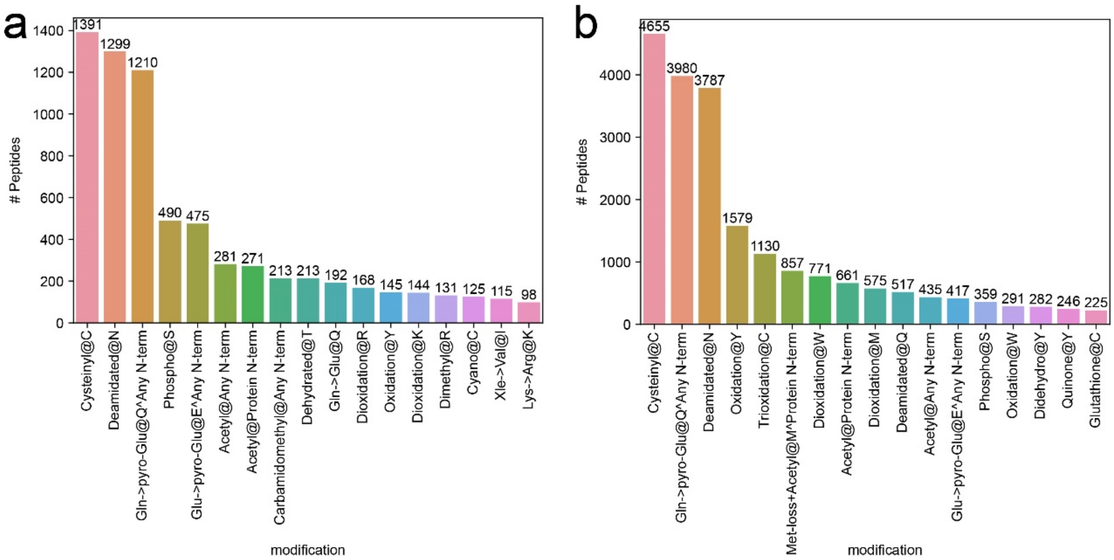

Supplementary Figure 10. Producer-consumer schema for rescoring with multiprocessing

The rectangle in the middle represents the main process, which contains model fine-tuning, prediction step on GPU, and the final Percolator rescoring. Other steps are processed in parallel, such as fragment matching, MS2 similarity calculation, and other feature extraction for Percolator rescoring. SA refers to spectral angle, and SPC refers to Spearman's rank correlation.

AlphaPeptDeep re-scores PSMs using a specified process number (8 by default).

All raw files are scheduled by the producer-consumer schema. After deep learning models are fine-tuned, the main process puts all RAW files in a queue and pops the first one into the producer processes for fragment extraction once there is an idle producer. The data will then be sent back to the main process for prediction, and this step is very fast using GPU. Next, the matched and predicted fragments will be sent to the consumer processes to extract all the 61-machine learning (ML) features. After all RAW files are processed, the PSMs and their ML features are combined in the main process for Percolator rescoring. From the perspective of each RAW file, it appears to be processed continuously without waiting for other RAW files.

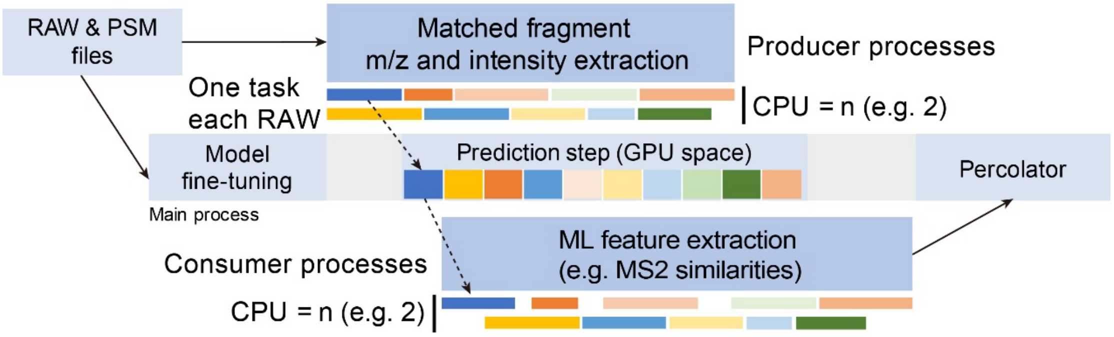

Supplement: Supplementary file 1 — Supplementary Information [file 41467_2022_34904_MOESM1_ESM.pdf]
