# Supplementary material for: AlphaPeptDeep: a modular deep learning framework to predict peptide properties for proteomics
Source: Nat Commun. 2022 Nov 24;13:7238. doi: 10.1038/s41467-022-34904-3 (PMC9700817; doi:10.1038/s41467-022-34904-3)
Supplement: Supplementary file 3 — Description of Additional Supplementary Files [file 41467_2022_34904_MOESM3_ESM.docx]

File Name: Supplementary Data 1

Description: Dataset information to train and test the MS2/RT/CCS data

File Name: Supplementary Data 2

Description: Description for 61 DL features for Percolator rescoring

File Name: Supplementary Data 3

Description: Mirrored MS2 plots for 21 PTMs of ProteomeTools

File Name: Supplementary Data 4

Description: Mirrored MS2 plots for randomly selected HLA-DDA peptides

File Name: Supplementary Data 5

Description: Elution profile and mirrored MS2 plots for randomly selected HLA-DIA peptides
