# Supplementary figures and images for: AlphaPeptDeep: a modular deep learning framework to predict peptide properties for proteomics (part 2 of 2)
Source: Nat Commun. 2022 Nov 24;13:7238. doi: 10.1038/s41467-022-34904-3 (PMC9700817; doi:10.1038/s41467-022-34904-3)

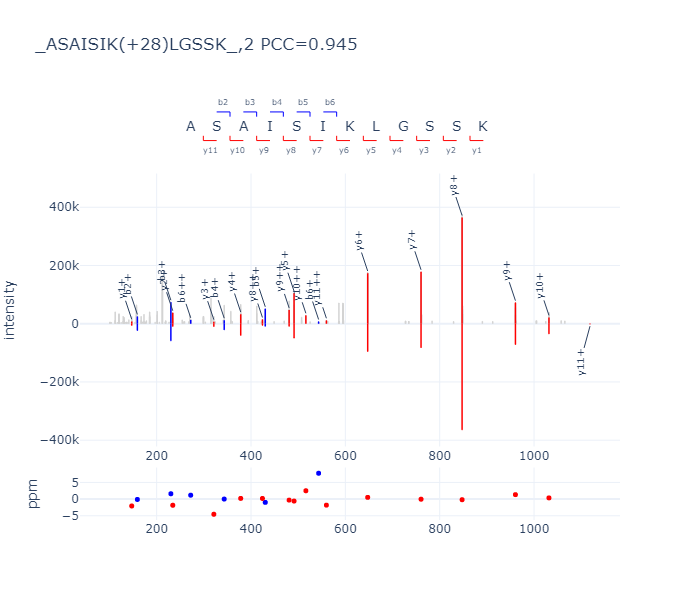

Supplement: Supplementary file 6 — Supplementary Data 3 [file 41467_2022_34904_MOESM6_ESM.zip › mirror-ms2-21ptm/Kmod_Formyl/_ASAISIK(+28)LGSSK_charge=2_nce=30_transfer_pcc=0.95.png]

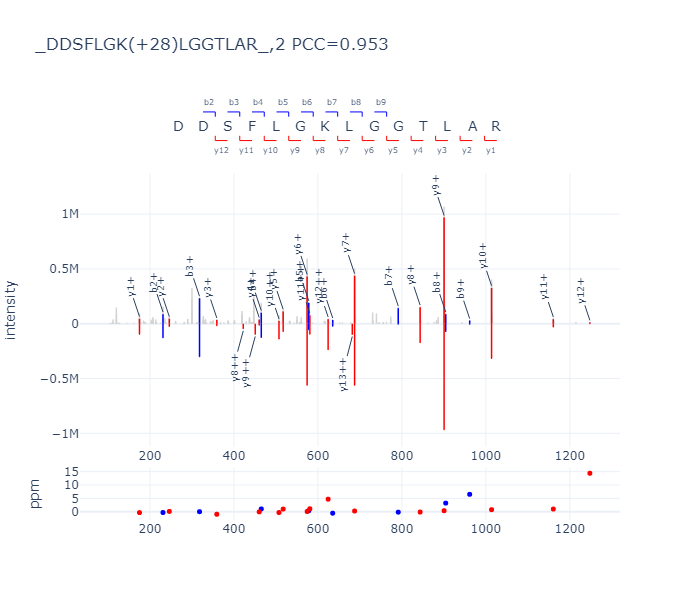

Supplement: Supplementary file 6 — Supplementary Data 3 [file 41467_2022_34904_MOESM6_ESM.zip › mirror-ms2-21ptm/Kmod_Formyl/_DDSFLGK(+28)LGGTLAR_charge=2_nce=30_pretrain_pcc=0.95.png]

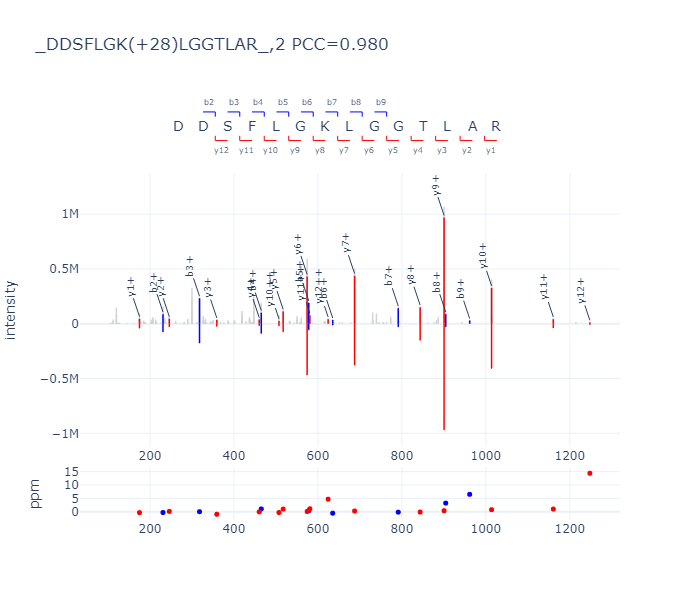

Supplement: Supplementary file 6 — Supplementary Data 3 [file 41467_2022_34904_MOESM6_ESM.zip › mirror-ms2-21ptm/Kmod_Formyl/_DDSFLGK(+28)LGGTLAR_charge=2_nce=30_transfer_pcc=0.98.png]

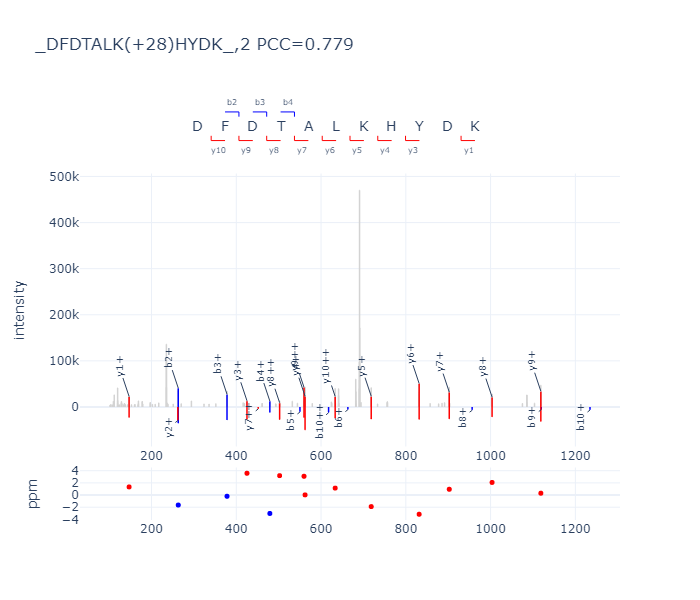

Supplement: Supplementary file 6 — Supplementary Data 3 [file 41467_2022_34904_MOESM6_ESM.zip › mirror-ms2-21ptm/Kmod_Formyl/_DFDTALK(+28)HYDK_charge=2_nce=30_pretrain_pcc=0.78.png]

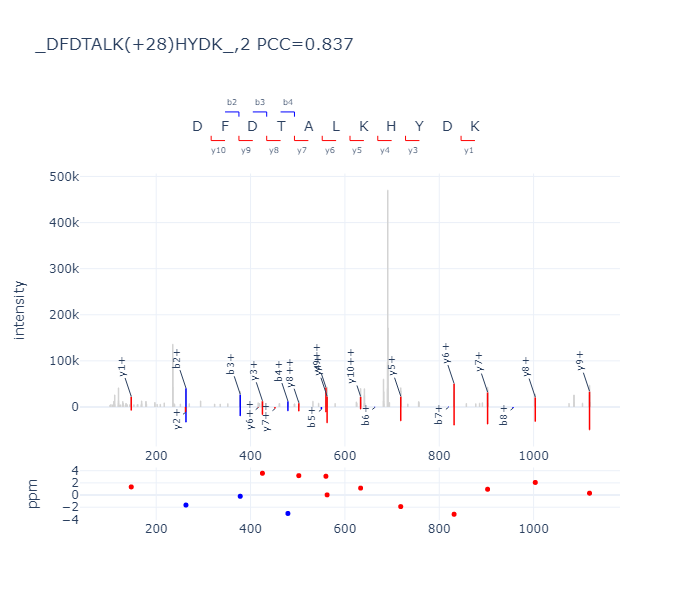

Supplement: Supplementary file 6 — Supplementary Data 3 [file 41467_2022_34904_MOESM6_ESM.zip › mirror-ms2-21ptm/Kmod_Formyl/_DFDTALK(+28)HYDK_charge=2_nce=30_transfer_pcc=0.84.png]

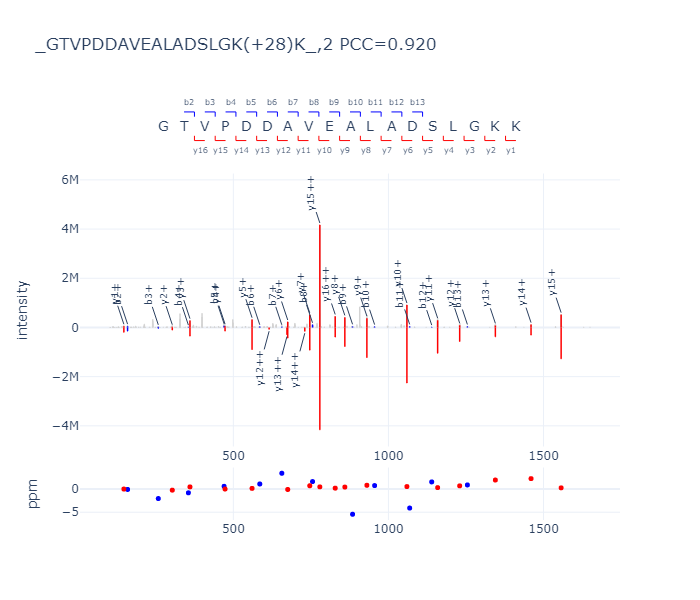

Supplement: Supplementary file 6 — Supplementary Data 3 [file 41467_2022_34904_MOESM6_ESM.zip › mirror-ms2-21ptm/Kmod_Formyl/_GTVPDDAVEALADSLGK(+28)K_charge=2_nce=30_pretrain_pcc=0.92.png]

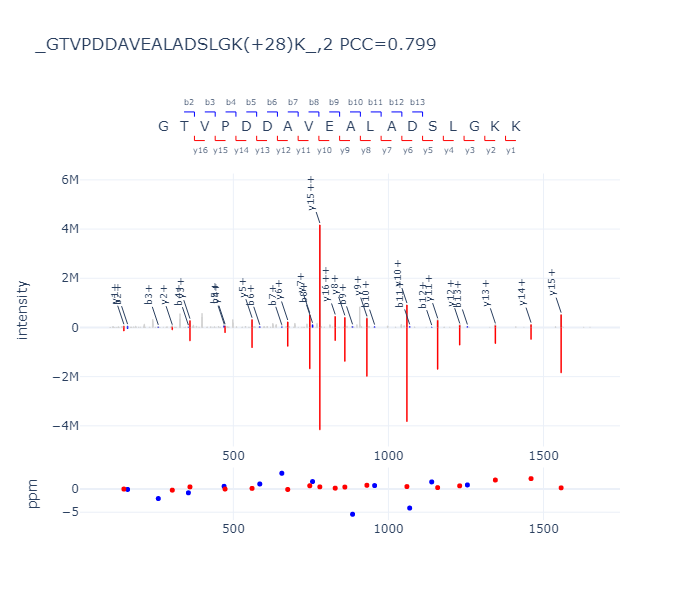

Supplement: Supplementary file 6 — Supplementary Data 3 [file 41467_2022_34904_MOESM6_ESM.zip › mirror-ms2-21ptm/Kmod_Formyl/_GTVPDDAVEALADSLGK(+28)K_charge=2_nce=30_transfer_pcc=0.80.png]

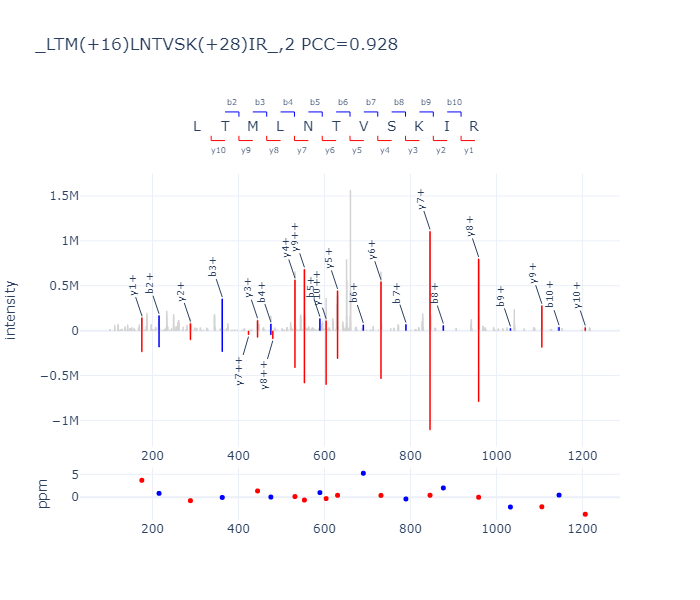

Supplement: Supplementary file 6 — Supplementary Data 3 [file 41467_2022_34904_MOESM6_ESM.zip › mirror-ms2-21ptm/Kmod_Formyl/_LTM(+16)LNTVSK(+28)IR_charge=2_nce=30_pretrain_pcc=0.93.png]

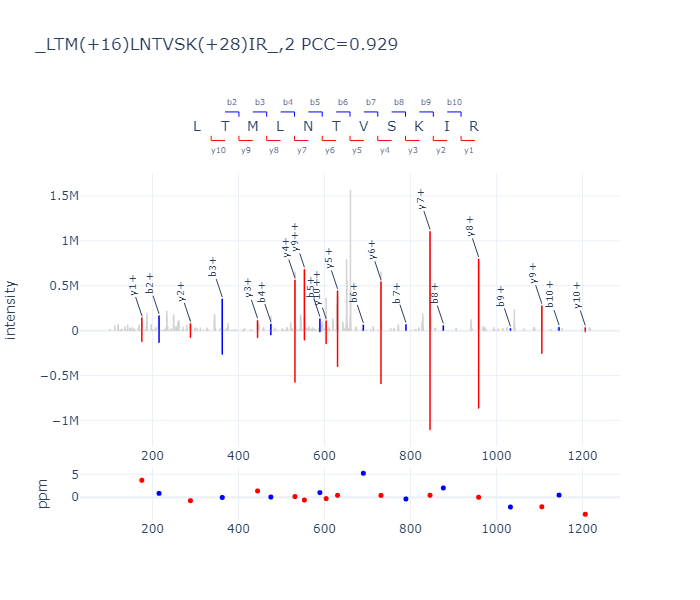

Supplement: Supplementary file 6 — Supplementary Data 3 [file 41467_2022_34904_MOESM6_ESM.zip › mirror-ms2-21ptm/Kmod_Formyl/_LTM(+16)LNTVSK(+28)IR_charge=2_nce=30_transfer_pcc=0.93.png]

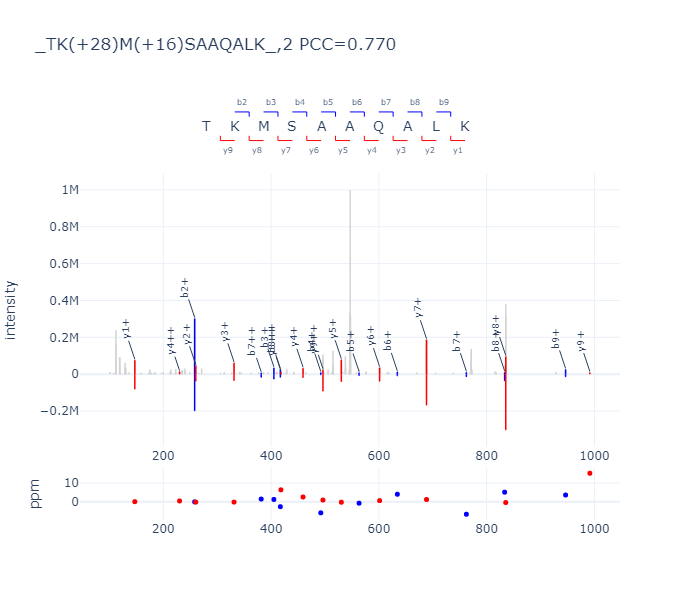

Supplement: Supplementary file 6 — Supplementary Data 3 [file 41467_2022_34904_MOESM6_ESM.zip › mirror-ms2-21ptm/Kmod_Formyl/_TK(+28)M(+16)SAAQALK_charge=2_nce=30_pretrain_pcc=0.77.png]

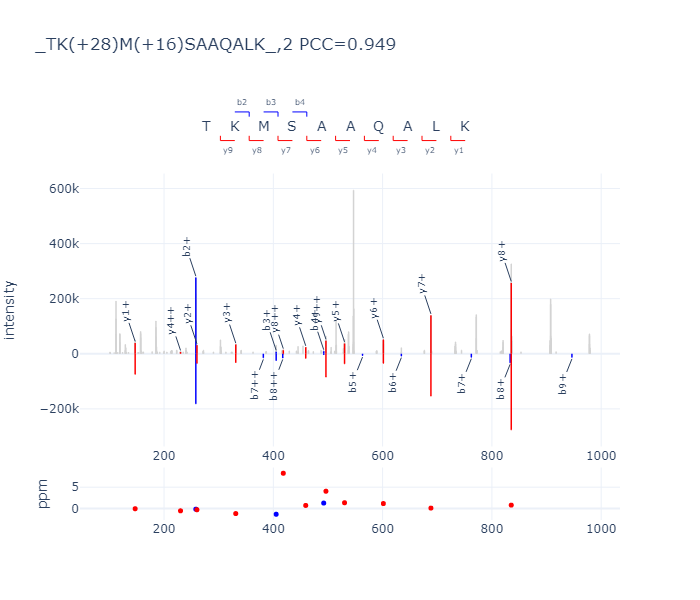

Supplement: Supplementary file 6 — Supplementary Data 3 [file 41467_2022_34904_MOESM6_ESM.zip › mirror-ms2-21ptm/Kmod_Formyl/_TK(+28)M(+16)SAAQALK_charge=2_nce=30_pretrain_pcc=0.95.png]

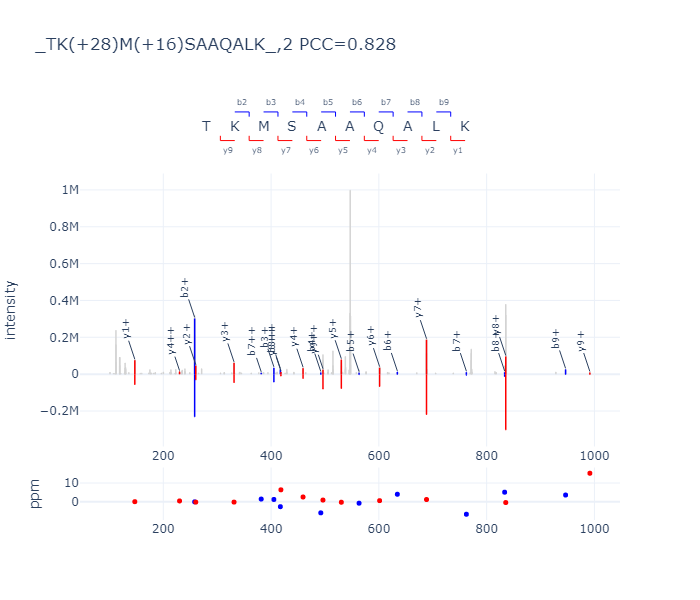

Supplement: Supplementary file 6 — Supplementary Data 3 [file 41467_2022_34904_MOESM6_ESM.zip › mirror-ms2-21ptm/Kmod_Formyl/_TK(+28)M(+16)SAAQALK_charge=2_nce=30_transfer_pcc=0.83.png]

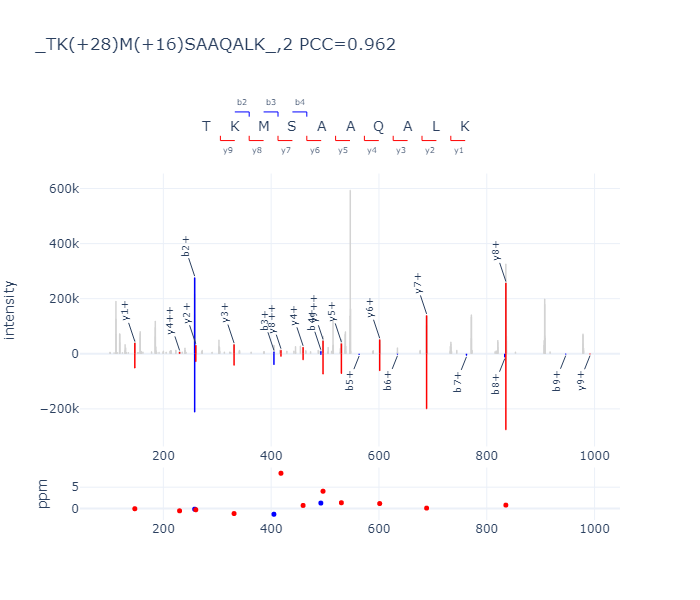

Supplement: Supplementary file 6 — Supplementary Data 3 [file 41467_2022_34904_MOESM6_ESM.zip › mirror-ms2-21ptm/Kmod_Formyl/_TK(+28)M(+16)SAAQALK_charge=2_nce=30_transfer_pcc=0.96.png]

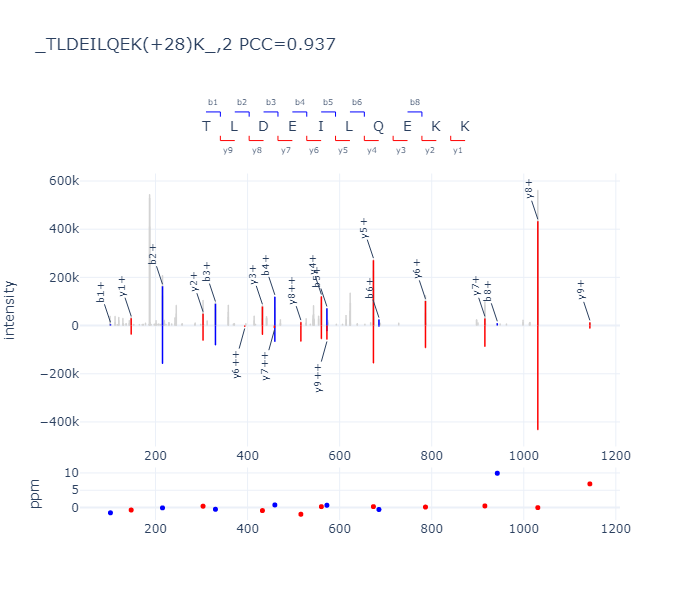

Supplement: Supplementary file 6 — Supplementary Data 3 [file 41467_2022_34904_MOESM6_ESM.zip › mirror-ms2-21ptm/Kmod_Formyl/_TLDEILQEK(+28)K_charge=2_nce=30_pretrain_pcc=0.94.png]

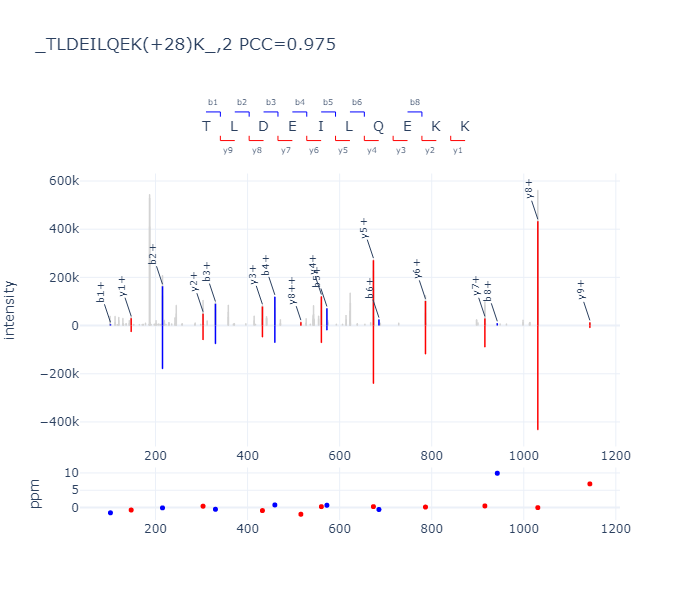

Supplement: Supplementary file 6 — Supplementary Data 3 [file 41467_2022_34904_MOESM6_ESM.zip › mirror-ms2-21ptm/Kmod_Formyl/_TLDEILQEK(+28)K_charge=2_nce=30_transfer_pcc=0.97.png]

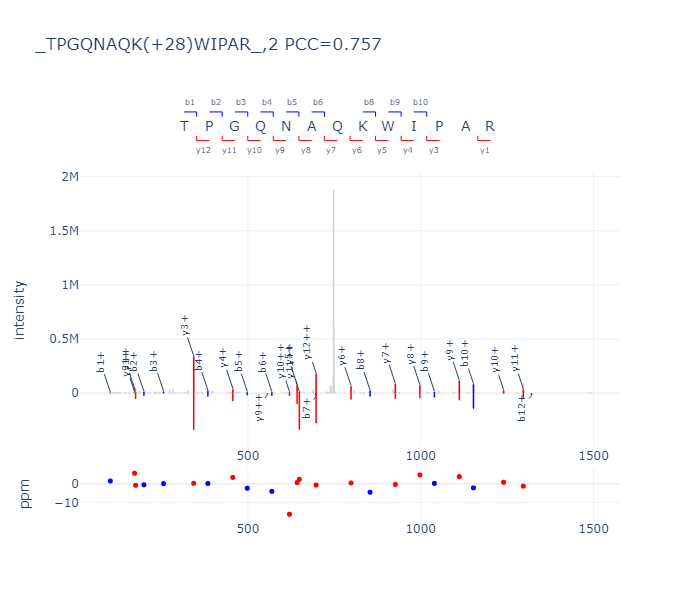

Supplement: Supplementary file 6 — Supplementary Data 3 [file 41467_2022_34904_MOESM6_ESM.zip › mirror-ms2-21ptm/Kmod_Formyl/_TPGQNAQK(+28)WIPAR_charge=2_nce=25_pretrain_pcc=0.76.png]

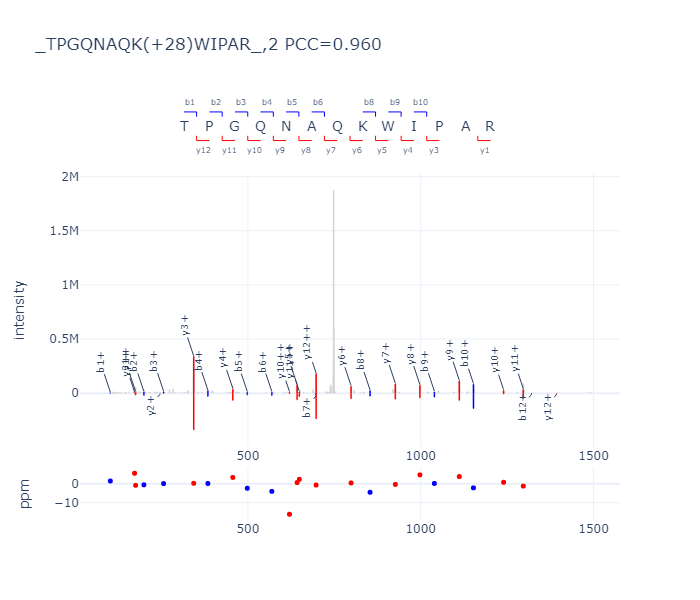

Supplement: Supplementary file 6 — Supplementary Data 3 [file 41467_2022_34904_MOESM6_ESM.zip › mirror-ms2-21ptm/Kmod_Formyl/_TPGQNAQK(+28)WIPAR_charge=2_nce=25_transfer_pcc=0.96.png]

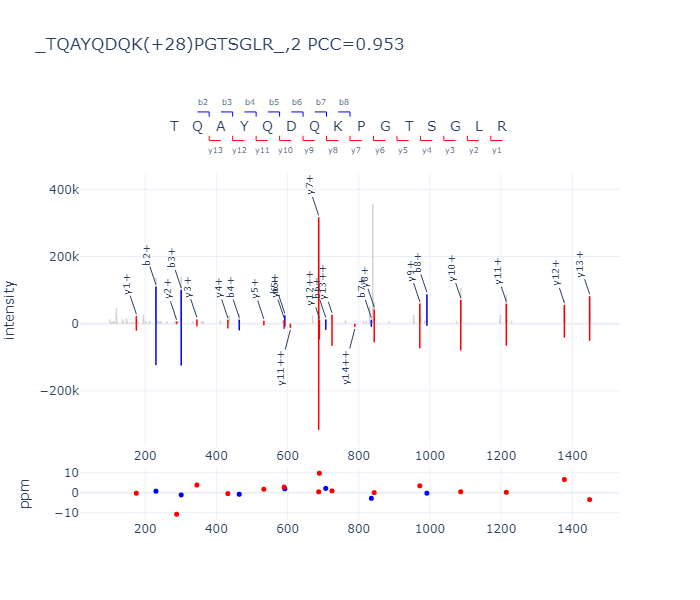

Supplement: Supplementary file 6 — Supplementary Data 3 [file 41467_2022_34904_MOESM6_ESM.zip › mirror-ms2-21ptm/Kmod_Formyl/_TQAYQDQK(+28)PGTSGLR_charge=2_nce=30_pretrain_pcc=0.95.png]

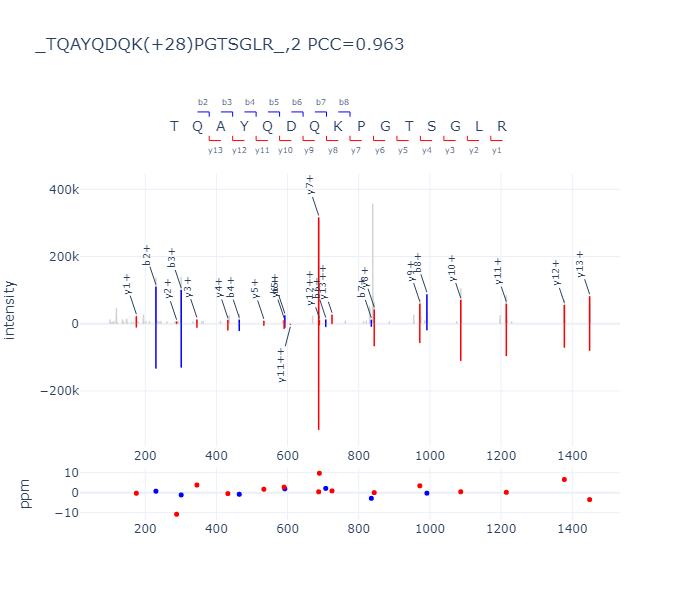

Supplement: Supplementary file 6 — Supplementary Data 3 [file 41467_2022_34904_MOESM6_ESM.zip › mirror-ms2-21ptm/Kmod_Formyl/_TQAYQDQK(+28)PGTSGLR_charge=2_nce=30_transfer_pcc=0.96.png]

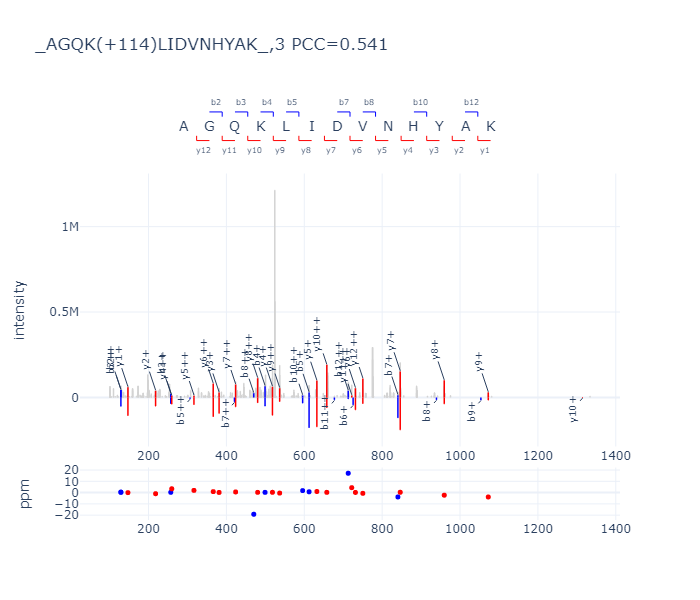

Supplement: Supplementary file 6 — Supplementary Data 3 [file 41467_2022_34904_MOESM6_ESM.zip › mirror-ms2-21ptm/Kmod_Glutaryl/_AGQK(+114)LIDVNHYAK_charge=3_nce=30_pretrain_pcc=0.54.png]

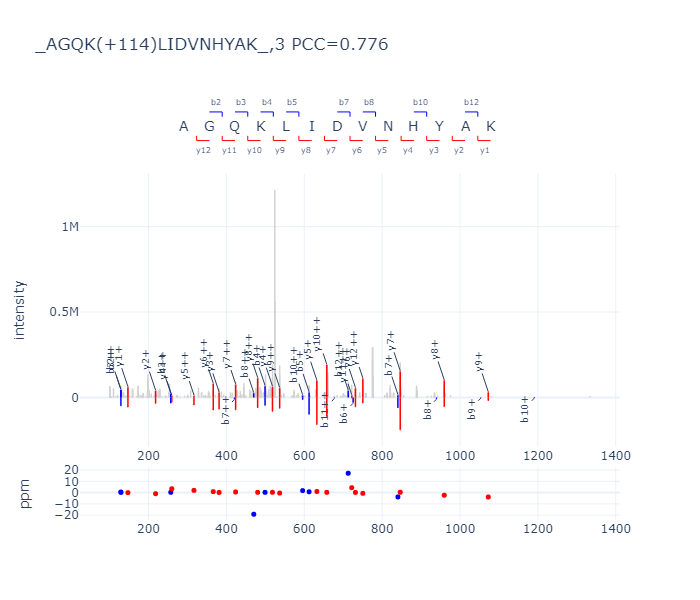

Supplement: Supplementary file 6 — Supplementary Data 3 [file 41467_2022_34904_MOESM6_ESM.zip › mirror-ms2-21ptm/Kmod_Glutaryl/_AGQK(+114)LIDVNHYAK_charge=3_nce=30_transfer_pcc=0.78.png]

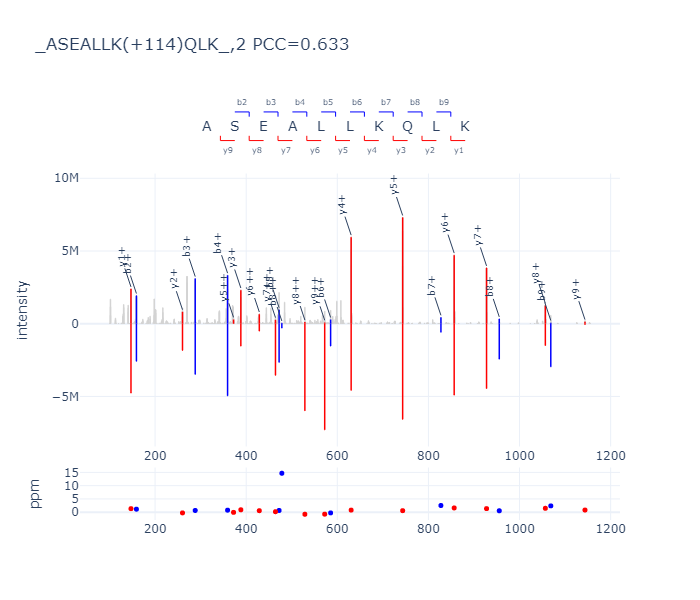

Supplement: Supplementary file 6 — Supplementary Data 3 [file 41467_2022_34904_MOESM6_ESM.zip › mirror-ms2-21ptm/Kmod_Glutaryl/_ASEALLK(+114)QLK_charge=2_nce=25_pretrain_pcc=0.63.png]

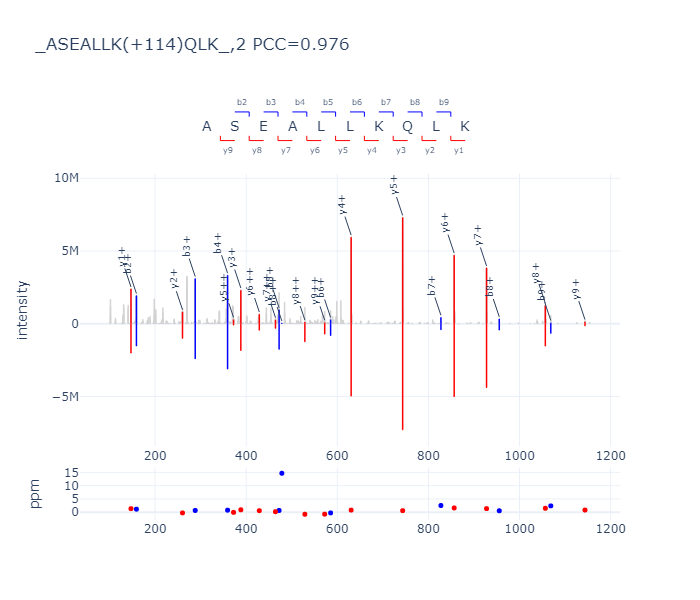

Supplement: Supplementary file 6 — Supplementary Data 3 [file 41467_2022_34904_MOESM6_ESM.zip › mirror-ms2-21ptm/Kmod_Glutaryl/_ASEALLK(+114)QLK_charge=2_nce=25_transfer_pcc=0.98.png]

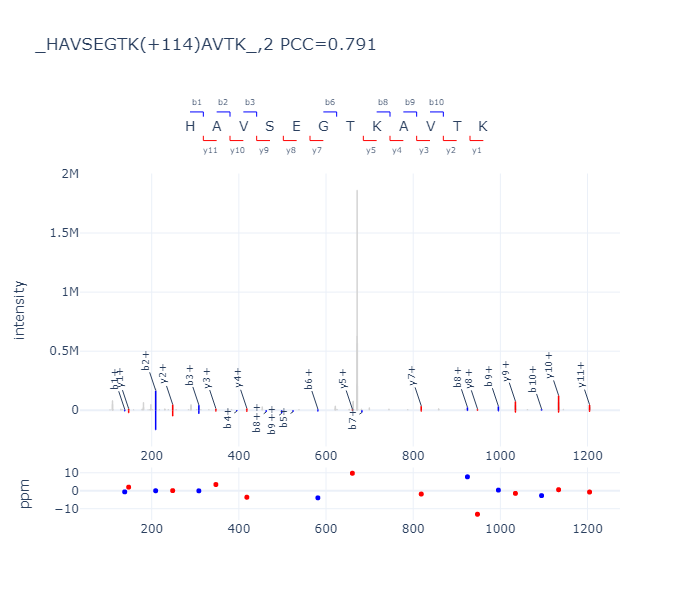

Supplement: Supplementary file 6 — Supplementary Data 3 [file 41467_2022_34904_MOESM6_ESM.zip › mirror-ms2-21ptm/Kmod_Glutaryl/_HAVSEGTK(+114)AVTK_charge=2_nce=35_pretrain_pcc=0.79.png]

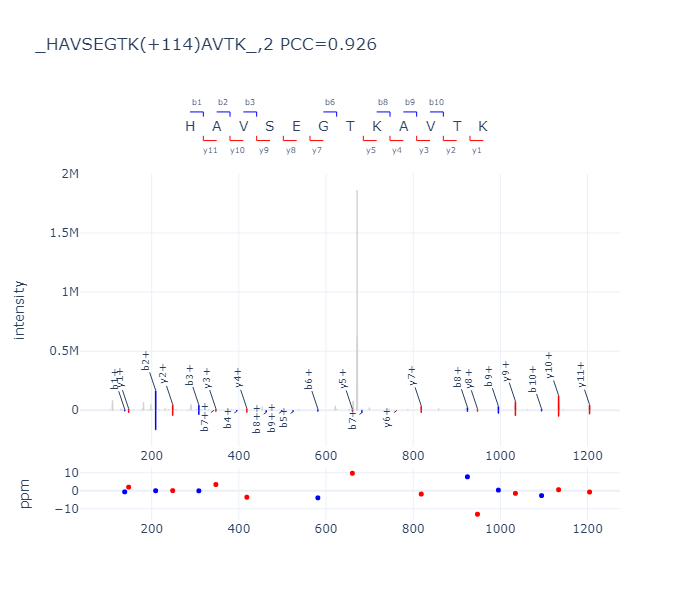

Supplement: Supplementary file 6 — Supplementary Data 3 [file 41467_2022_34904_MOESM6_ESM.zip › mirror-ms2-21ptm/Kmod_Glutaryl/_HAVSEGTK(+114)AVTK_charge=2_nce=35_transfer_pcc=0.93.png]

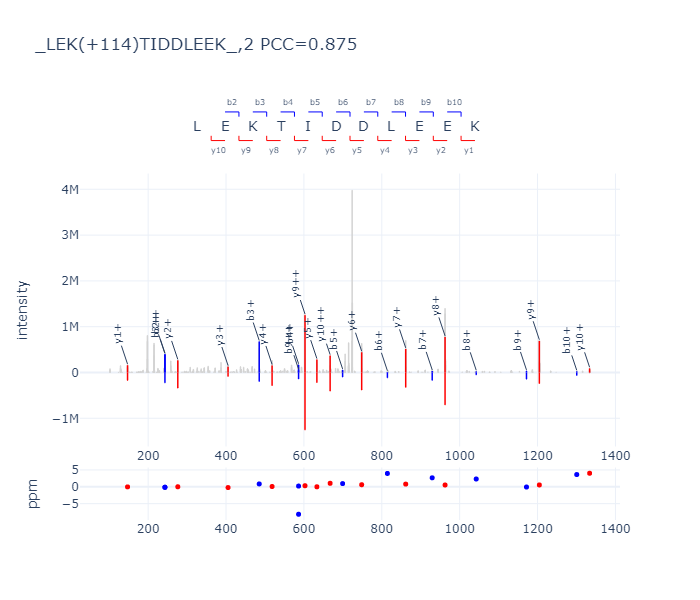

Supplement: Supplementary file 6 — Supplementary Data 3 [file 41467_2022_34904_MOESM6_ESM.zip › mirror-ms2-21ptm/Kmod_Glutaryl/_LEK(+114)TIDDLEEK_charge=2_nce=25_pretrain_pcc=0.88.png]

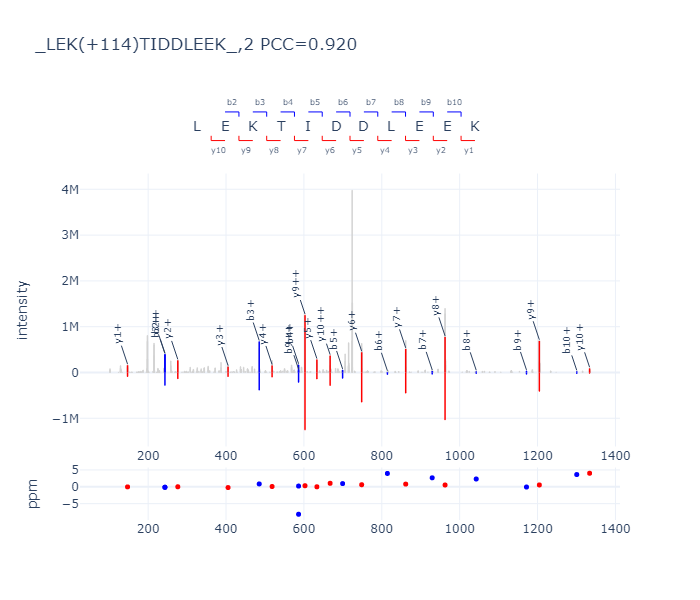

Supplement: Supplementary file 6 — Supplementary Data 3 [file 41467_2022_34904_MOESM6_ESM.zip › mirror-ms2-21ptm/Kmod_Glutaryl/_LEK(+114)TIDDLEEK_charge=2_nce=25_transfer_pcc=0.92.png]

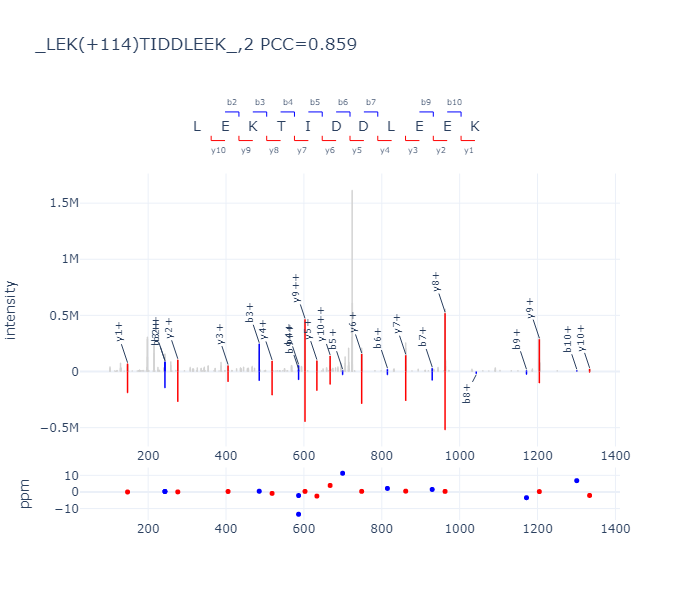

Supplement: Supplementary file 6 — Supplementary Data 3 [file 41467_2022_34904_MOESM6_ESM.zip › mirror-ms2-21ptm/Kmod_Glutaryl/_LEK(+114)TIDDLEEK_charge=2_nce=30_pretrain_pcc=0.86.png]

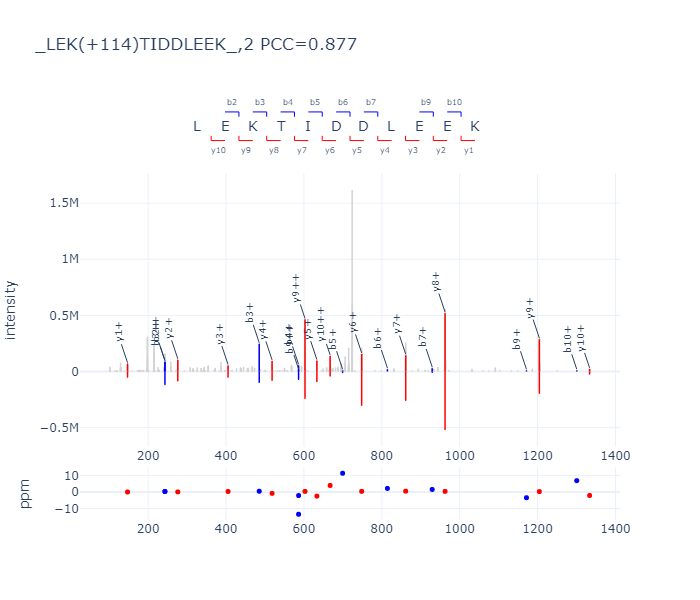

Supplement: Supplementary file 6 — Supplementary Data 3 [file 41467_2022_34904_MOESM6_ESM.zip › mirror-ms2-21ptm/Kmod_Glutaryl/_LEK(+114)TIDDLEEK_charge=2_nce=30_transfer_pcc=0.88.png]

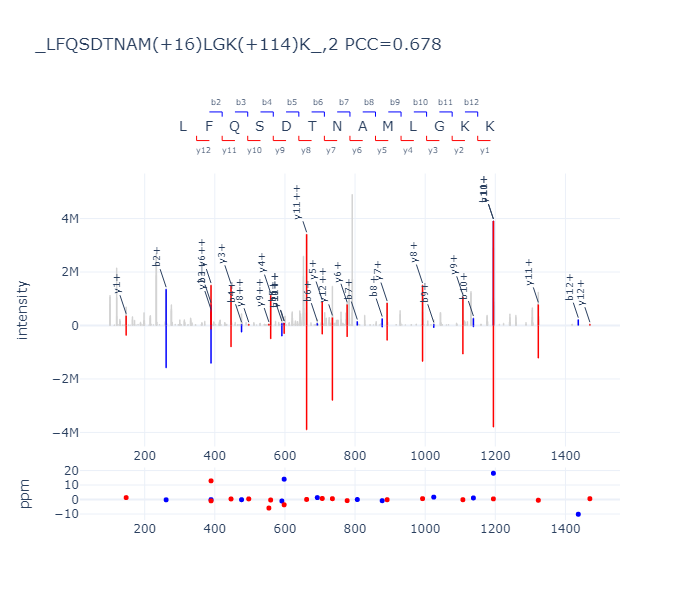

Supplement: Supplementary file 6 — Supplementary Data 3 [file 41467_2022_34904_MOESM6_ESM.zip › mirror-ms2-21ptm/Kmod_Glutaryl/_LFQSDTNAM(+16)LGK(+114)K_charge=2_nce=25_pretrain_pcc=0.68.png]

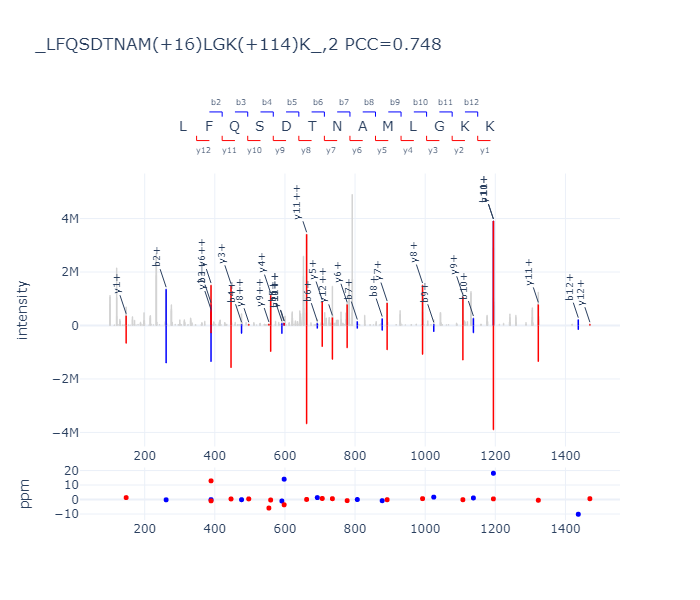

Supplement: Supplementary file 6 — Supplementary Data 3 [file 41467_2022_34904_MOESM6_ESM.zip › mirror-ms2-21ptm/Kmod_Glutaryl/_LFQSDTNAM(+16)LGK(+114)K_charge=2_nce=25_transfer_pcc=0.75.png]

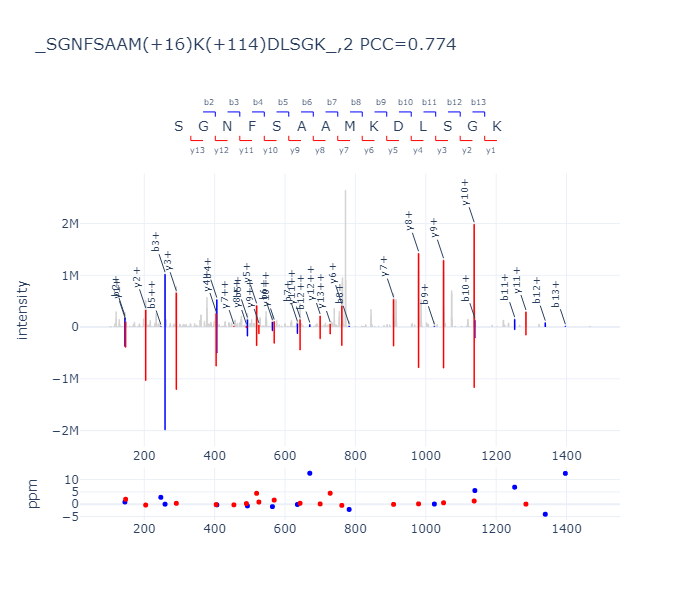

Supplement: Supplementary file 6 — Supplementary Data 3 [file 41467_2022_34904_MOESM6_ESM.zip › mirror-ms2-21ptm/Kmod_Glutaryl/_SGNFSAAM(+16)K(+114)DLSGK_charge=2_nce=30_pretrain_pcc=0.77.png]

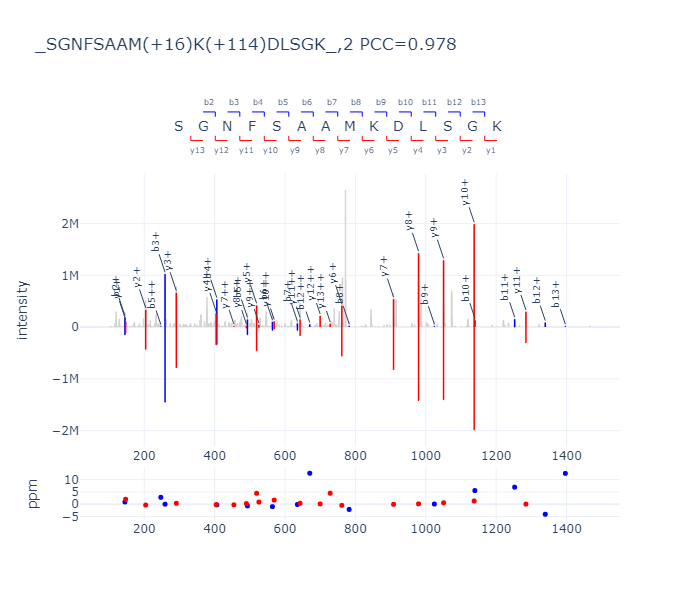

Supplement: Supplementary file 6 — Supplementary Data 3 [file 41467_2022_34904_MOESM6_ESM.zip › mirror-ms2-21ptm/Kmod_Glutaryl/_SGNFSAAM(+16)K(+114)DLSGK_charge=2_nce=30_transfer_pcc=0.98.png]

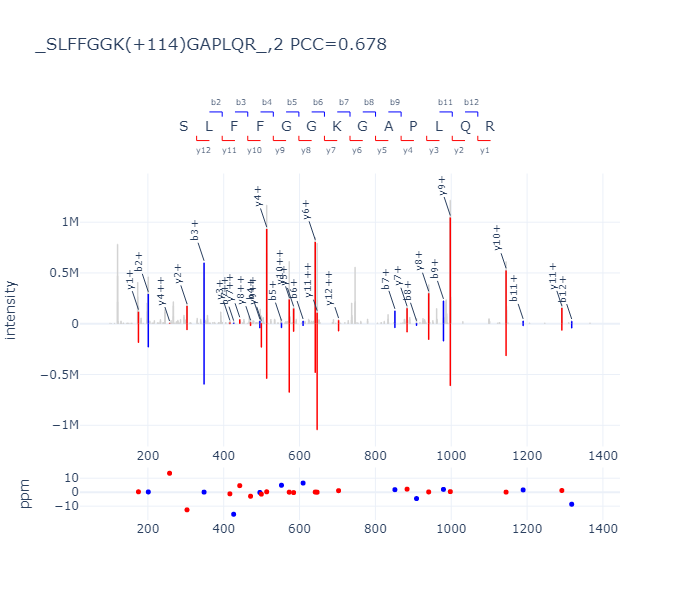

Supplement: Supplementary file 6 — Supplementary Data 3 [file 41467_2022_34904_MOESM6_ESM.zip › mirror-ms2-21ptm/Kmod_Glutaryl/_SLFFGGK(+114)GAPLQR_charge=2_nce=25_pretrain_pcc=0.68.png]

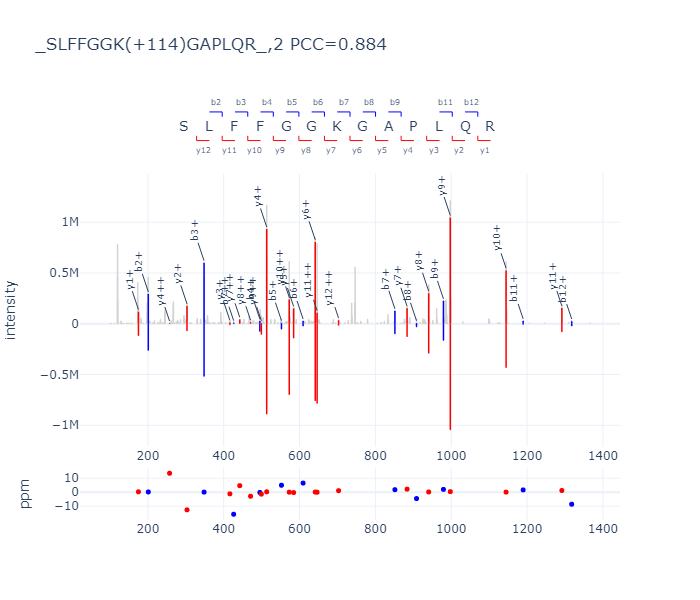

Supplement: Supplementary file 6 — Supplementary Data 3 [file 41467_2022_34904_MOESM6_ESM.zip › mirror-ms2-21ptm/Kmod_Glutaryl/_SLFFGGK(+114)GAPLQR_charge=2_nce=25_transfer_pcc=0.88.png]

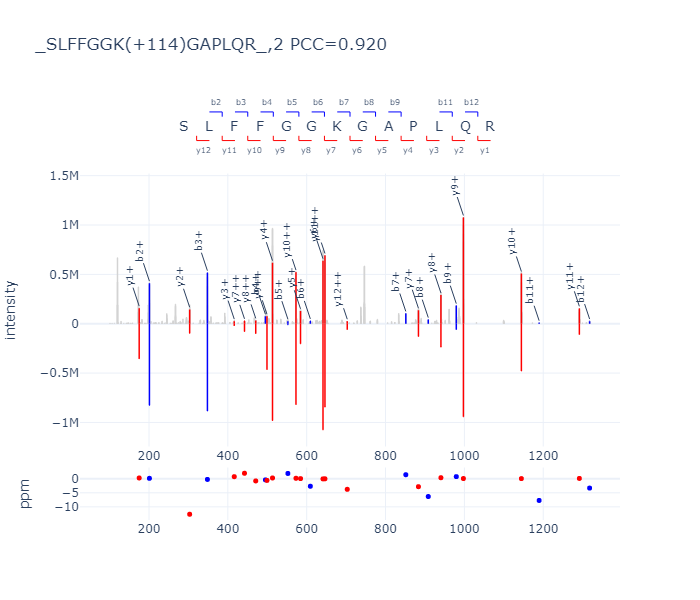

Supplement: Supplementary file 6 — Supplementary Data 3 [file 41467_2022_34904_MOESM6_ESM.zip › mirror-ms2-21ptm/Kmod_Glutaryl/_SLFFGGK(+114)GAPLQR_charge=2_nce=30_pretrain_pcc=0.92.png]

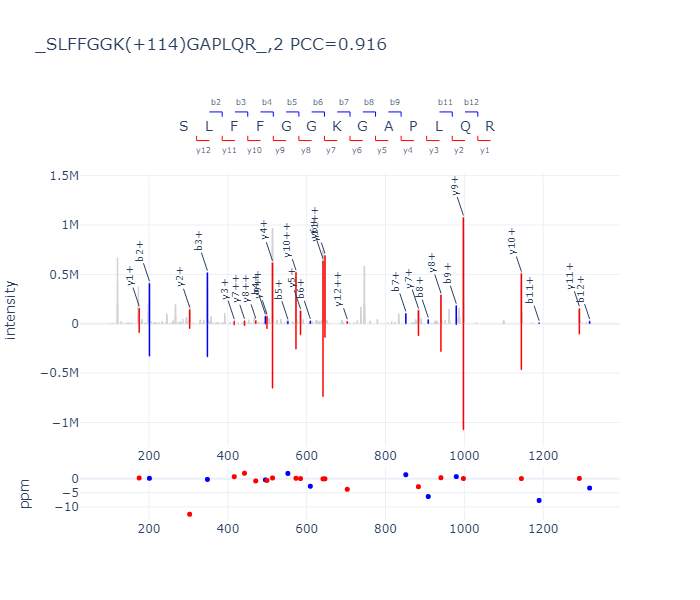

Supplement: Supplementary file 6 — Supplementary Data 3 [file 41467_2022_34904_MOESM6_ESM.zip › mirror-ms2-21ptm/Kmod_Glutaryl/_SLFFGGK(+114)GAPLQR_charge=2_nce=30_transfer_pcc=0.92.png]

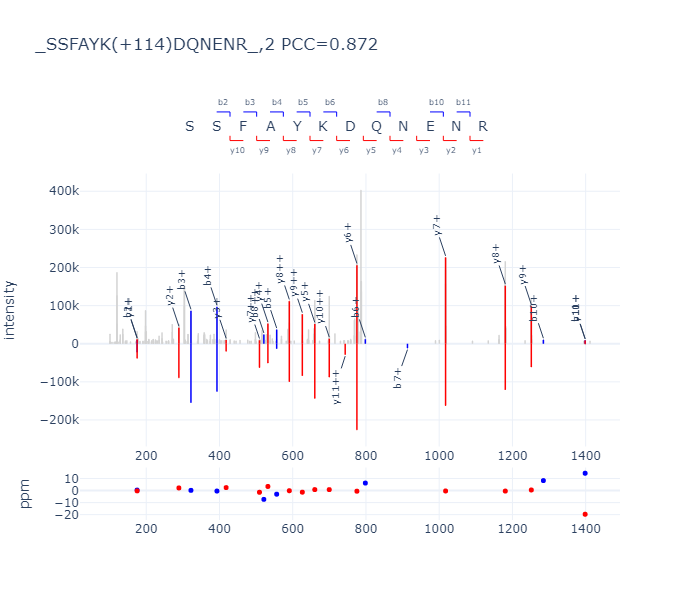

Supplement: Supplementary file 6 — Supplementary Data 3 [file 41467_2022_34904_MOESM6_ESM.zip › mirror-ms2-21ptm/Kmod_Glutaryl/_SSFAYK(+114)DQNENR_charge=2_nce=30_pretrain_pcc=0.87.png]

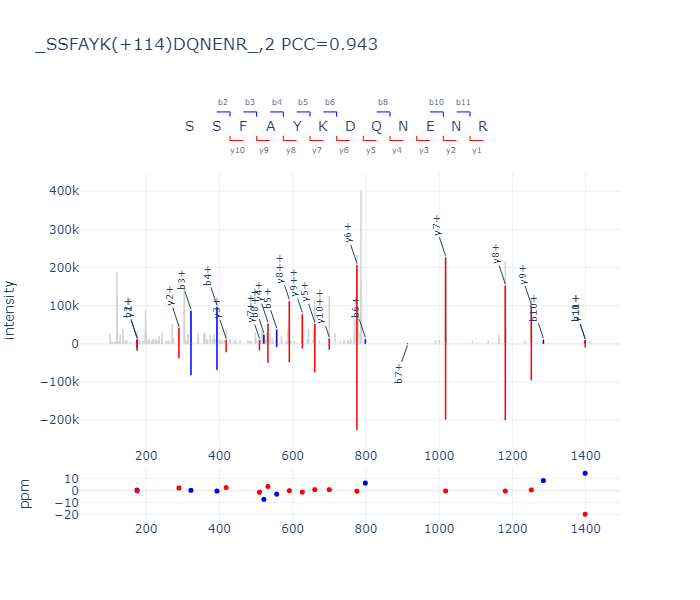

Supplement: Supplementary file 6 — Supplementary Data 3 [file 41467_2022_34904_MOESM6_ESM.zip › mirror-ms2-21ptm/Kmod_Glutaryl/_SSFAYK(+114)DQNENR_charge=2_nce=30_transfer_pcc=0.94.png]

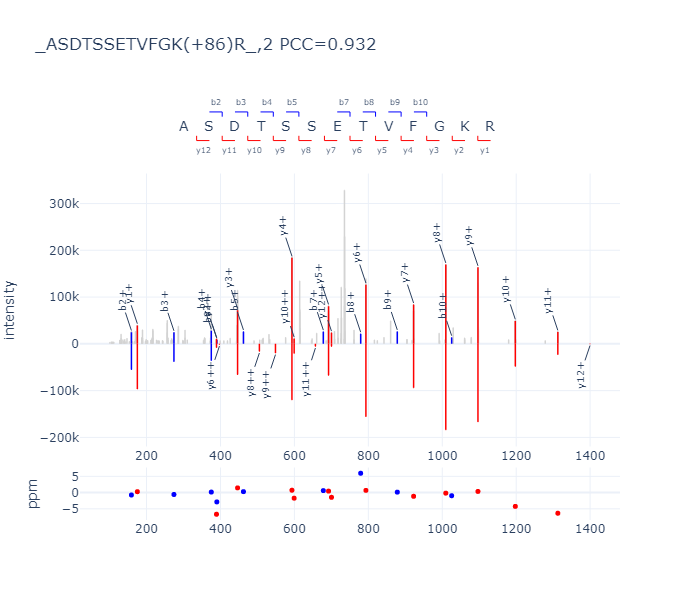

Supplement: Supplementary file 6 — Supplementary Data 3 [file 41467_2022_34904_MOESM6_ESM.zip › mirror-ms2-21ptm/Kmod_Hydroxy/_ASDTSSETVFGK(+86)R_charge=2_nce=35_pretrain_pcc=0.93.png]

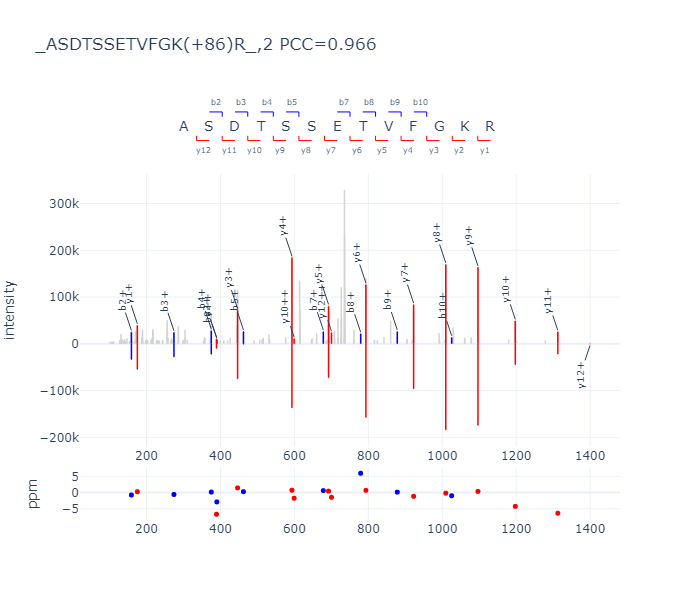

Supplement: Supplementary file 6 — Supplementary Data 3 [file 41467_2022_34904_MOESM6_ESM.zip › mirror-ms2-21ptm/Kmod_Hydroxy/_ASDTSSETVFGK(+86)R_charge=2_nce=35_transfer_pcc=0.97.png]

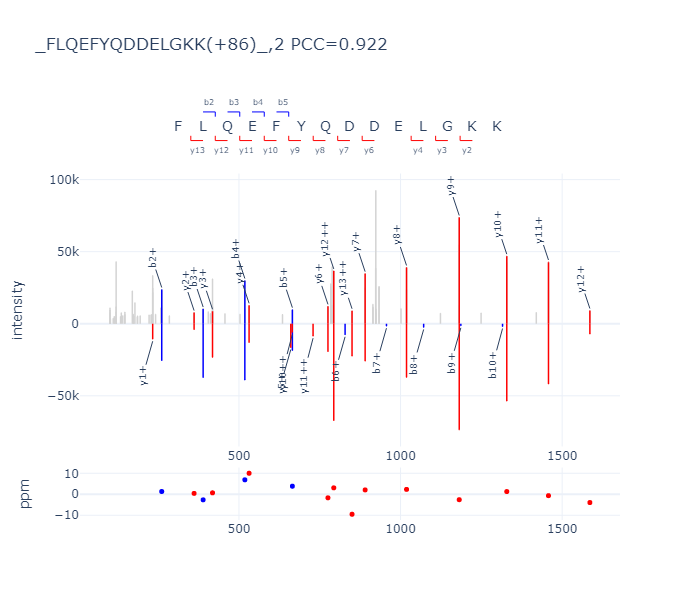

Supplement: Supplementary file 6 — Supplementary Data 3 [file 41467_2022_34904_MOESM6_ESM.zip › mirror-ms2-21ptm/Kmod_Hydroxy/_FLQEFYQDDELGKK(+86)_charge=2_nce=25_pretrain_pcc=0.92.png]

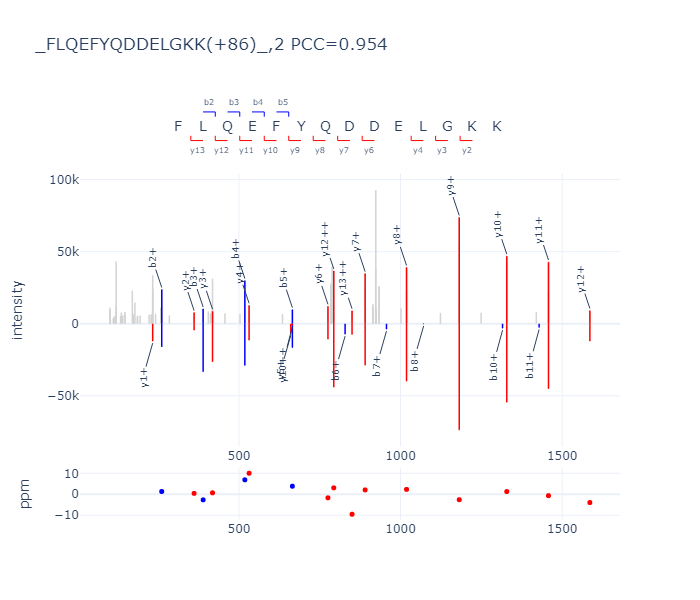

Supplement: Supplementary file 6 — Supplementary Data 3 [file 41467_2022_34904_MOESM6_ESM.zip › mirror-ms2-21ptm/Kmod_Hydroxy/_FLQEFYQDDELGKK(+86)_charge=2_nce=25_transfer_pcc=0.95.png]

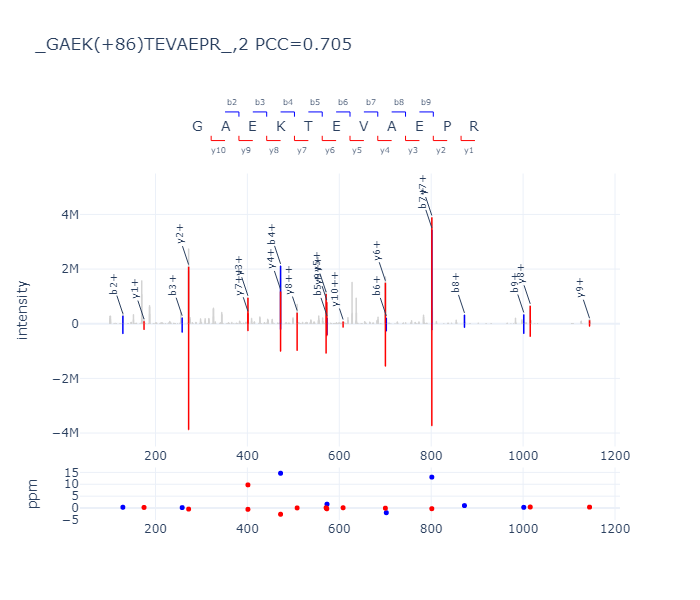

Supplement: Supplementary file 6 — Supplementary Data 3 [file 41467_2022_34904_MOESM6_ESM.zip › mirror-ms2-21ptm/Kmod_Hydroxy/_GAEK(+86)TEVAEPR_charge=2_nce=30_pretrain_pcc=0.70.png]

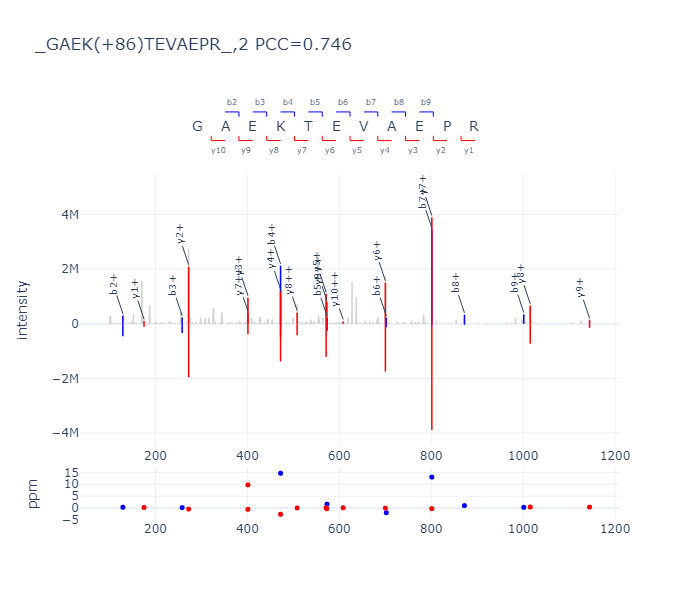

Supplement: Supplementary file 6 — Supplementary Data 3 [file 41467_2022_34904_MOESM6_ESM.zip › mirror-ms2-21ptm/Kmod_Hydroxy/_GAEK(+86)TEVAEPR_charge=2_nce=30_transfer_pcc=0.75.png]

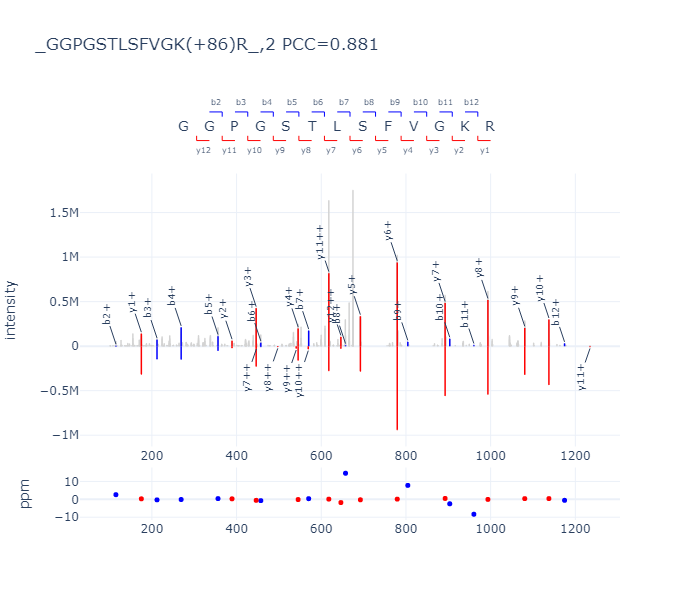

Supplement: Supplementary file 6 — Supplementary Data 3 [file 41467_2022_34904_MOESM6_ESM.zip › mirror-ms2-21ptm/Kmod_Hydroxy/_GGPGSTLSFVGK(+86)R_charge=2_nce=35_pretrain_pcc=0.88.png]

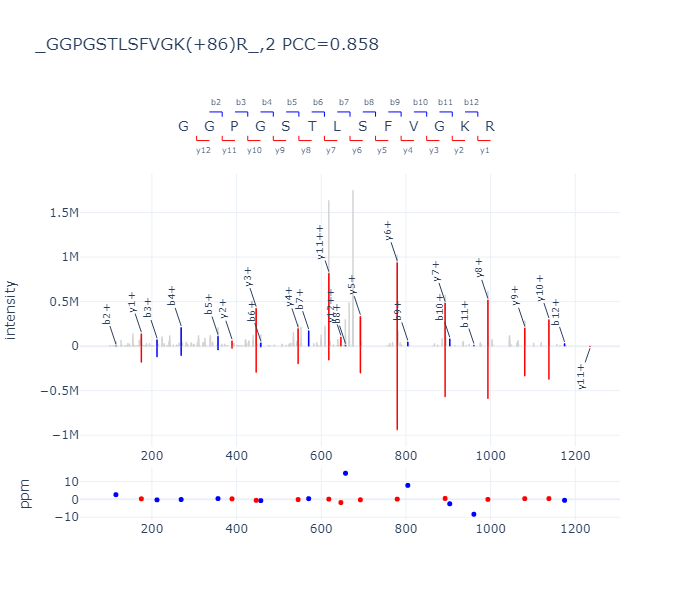

Supplement: Supplementary file 6 — Supplementary Data 3 [file 41467_2022_34904_MOESM6_ESM.zip › mirror-ms2-21ptm/Kmod_Hydroxy/_GGPGSTLSFVGK(+86)R_charge=2_nce=35_transfer_pcc=0.86.png]

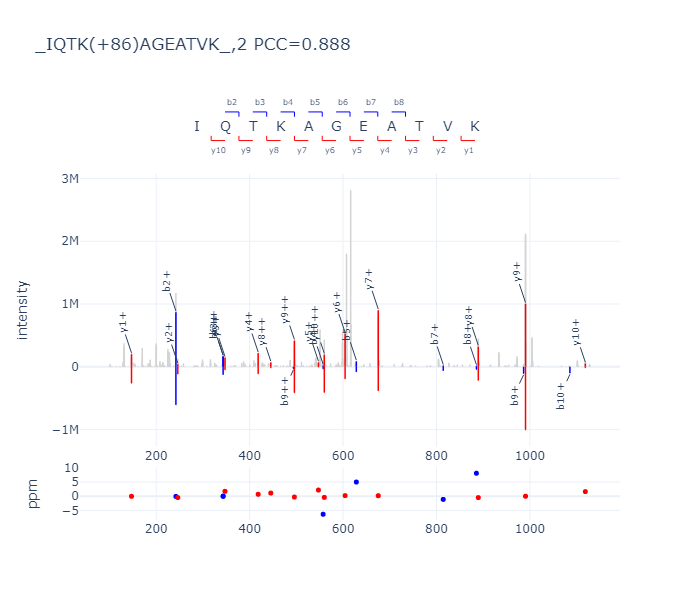

Supplement: Supplementary file 6 — Supplementary Data 3 [file 41467_2022_34904_MOESM6_ESM.zip › mirror-ms2-21ptm/Kmod_Hydroxy/_IQTK(+86)AGEATVK_charge=2_nce=25_pretrain_pcc=0.89.png]

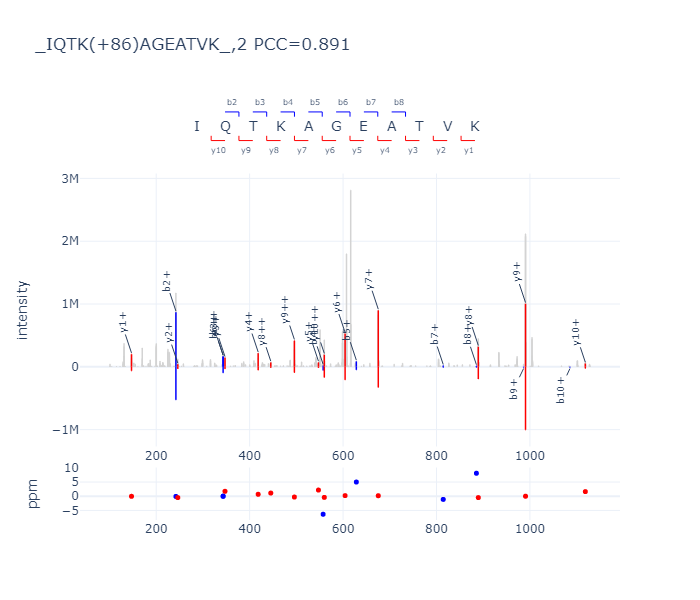

Supplement: Supplementary file 6 — Supplementary Data 3 [file 41467_2022_34904_MOESM6_ESM.zip › mirror-ms2-21ptm/Kmod_Hydroxy/_IQTK(+86)AGEATVK_charge=2_nce=25_transfer_pcc=0.89.png]

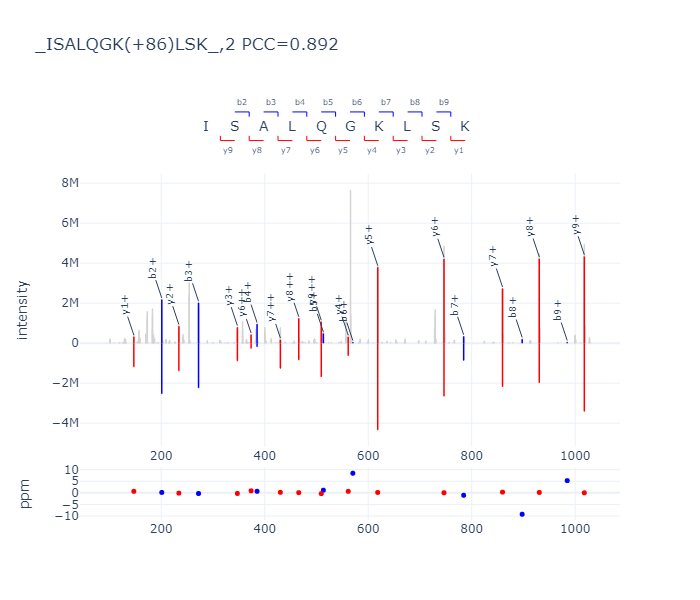

Supplement: Supplementary file 6 — Supplementary Data 3 [file 41467_2022_34904_MOESM6_ESM.zip › mirror-ms2-21ptm/Kmod_Hydroxy/_ISALQGK(+86)LSK_charge=2_nce=35_pretrain_pcc=0.89.png]

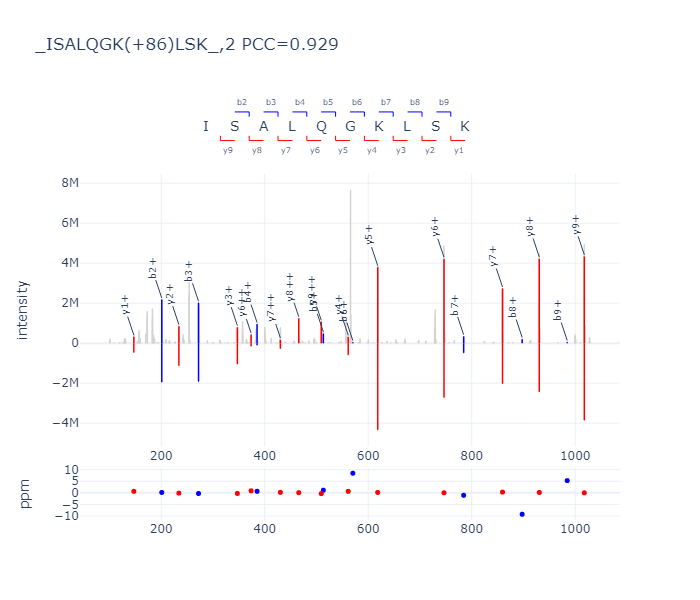

Supplement: Supplementary file 6 — Supplementary Data 3 [file 41467_2022_34904_MOESM6_ESM.zip › mirror-ms2-21ptm/Kmod_Hydroxy/_ISALQGK(+86)LSK_charge=2_nce=35_transfer_pcc=0.93.png]

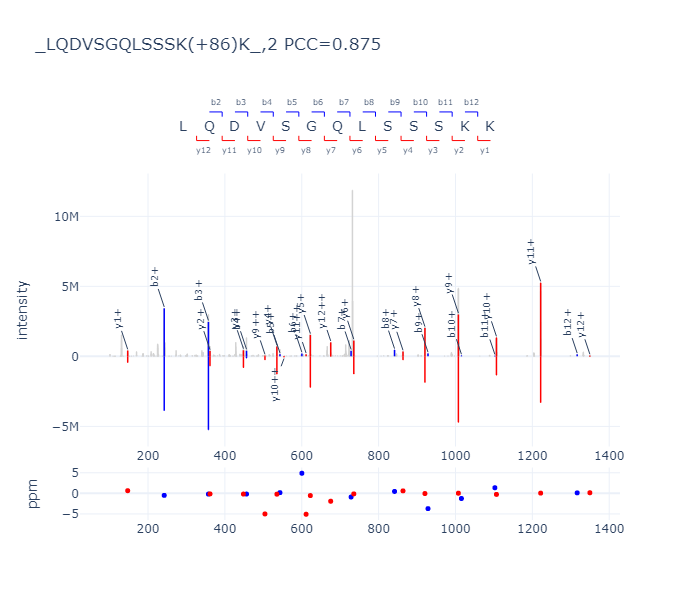

Supplement: Supplementary file 6 — Supplementary Data 3 [file 41467_2022_34904_MOESM6_ESM.zip › mirror-ms2-21ptm/Kmod_Hydroxy/_LQDVSGQLSSSK(+86)K_charge=2_nce=35_pretrain_pcc=0.88.png]

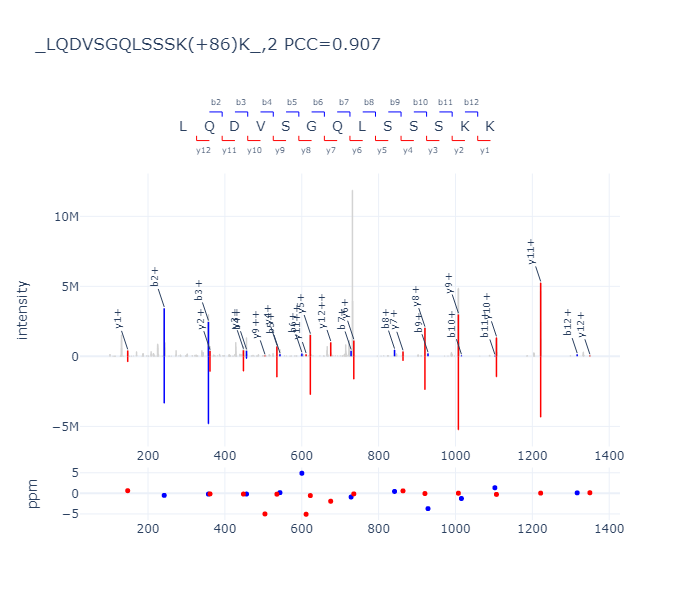

Supplement: Supplementary file 6 — Supplementary Data 3 [file 41467_2022_34904_MOESM6_ESM.zip › mirror-ms2-21ptm/Kmod_Hydroxy/_LQDVSGQLSSSK(+86)K_charge=2_nce=35_transfer_pcc=0.91.png]

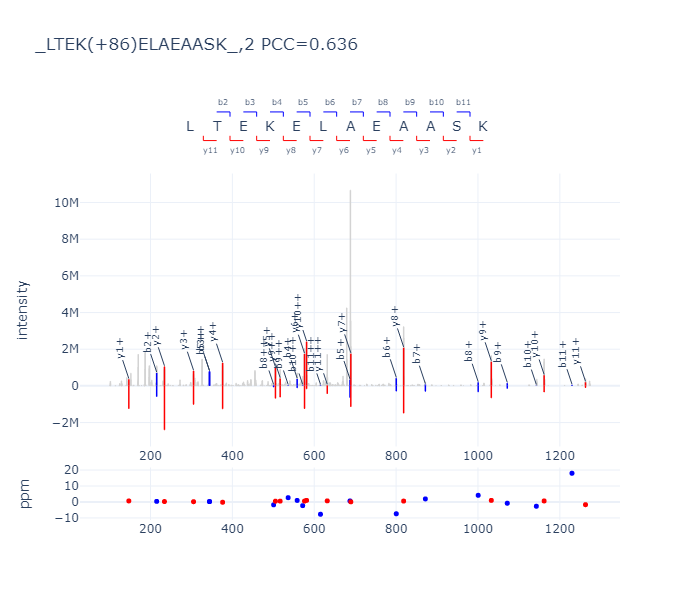

Supplement: Supplementary file 6 — Supplementary Data 3 [file 41467_2022_34904_MOESM6_ESM.zip › mirror-ms2-21ptm/Kmod_Hydroxy/_LTEK(+86)ELAEAASK_charge=2_nce=35_pretrain_pcc=0.64.png]

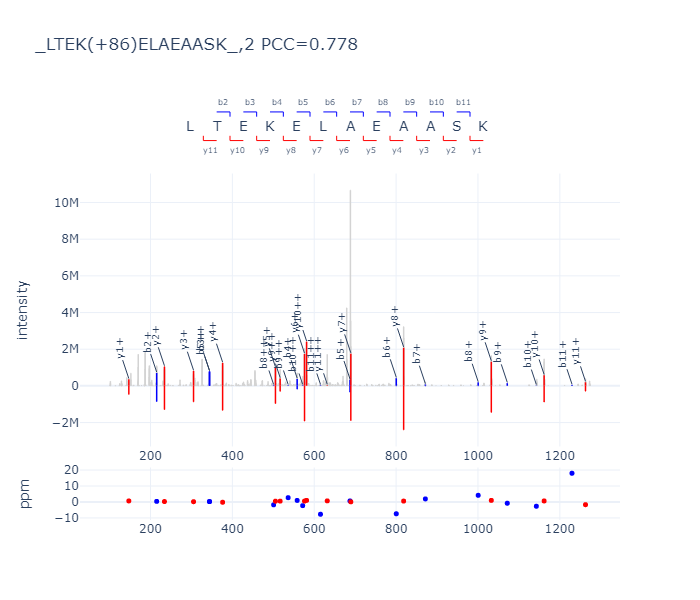

Supplement: Supplementary file 6 — Supplementary Data 3 [file 41467_2022_34904_MOESM6_ESM.zip › mirror-ms2-21ptm/Kmod_Hydroxy/_LTEK(+86)ELAEAASK_charge=2_nce=35_transfer_pcc=0.78.png]

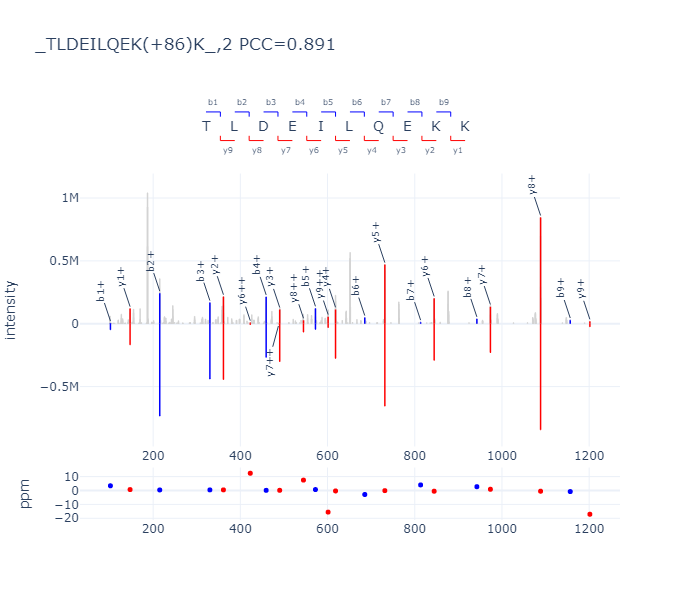

Supplement: Supplementary file 6 — Supplementary Data 3 [file 41467_2022_34904_MOESM6_ESM.zip › mirror-ms2-21ptm/Kmod_Hydroxy/_TLDEILQEK(+86)K_charge=2_nce=35_pretrain_pcc=0.89.png]

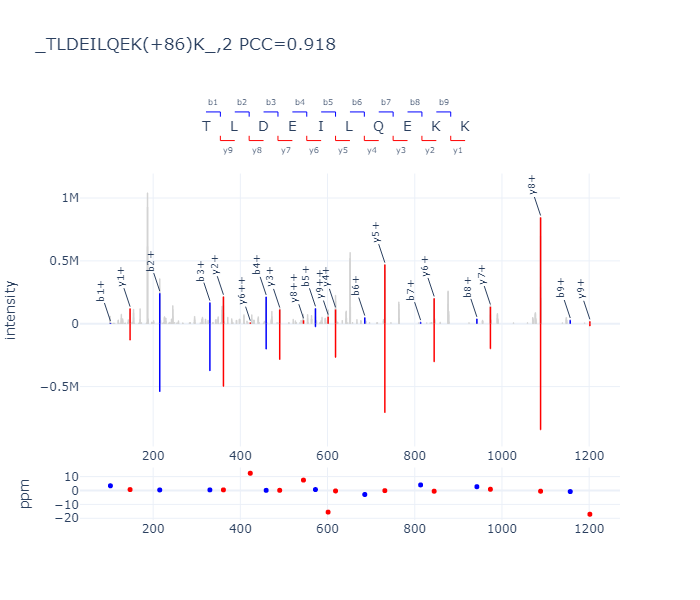

Supplement: Supplementary file 6 — Supplementary Data 3 [file 41467_2022_34904_MOESM6_ESM.zip › mirror-ms2-21ptm/Kmod_Hydroxy/_TLDEILQEK(+86)K_charge=2_nce=35_transfer_pcc=0.92.png]

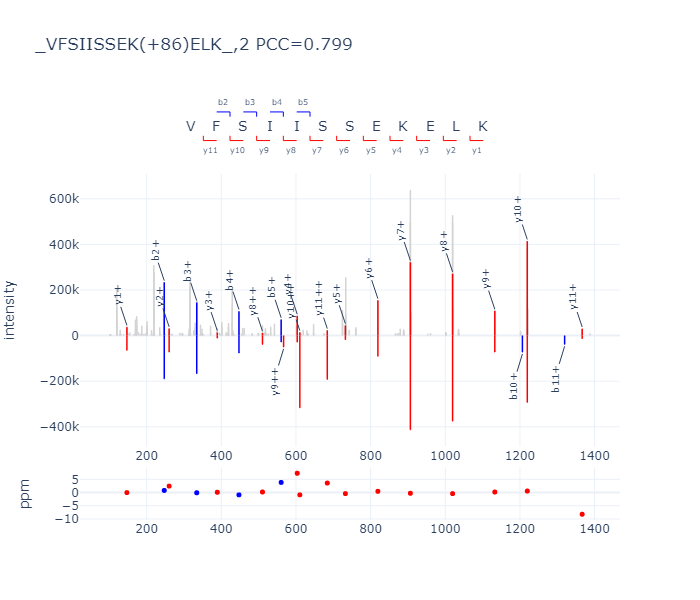

Supplement: Supplementary file 6 — Supplementary Data 3 [file 41467_2022_34904_MOESM6_ESM.zip › mirror-ms2-21ptm/Kmod_Hydroxy/_VFSIISSEK(+86)ELK_charge=2_nce=25_pretrain_pcc=0.80.png]

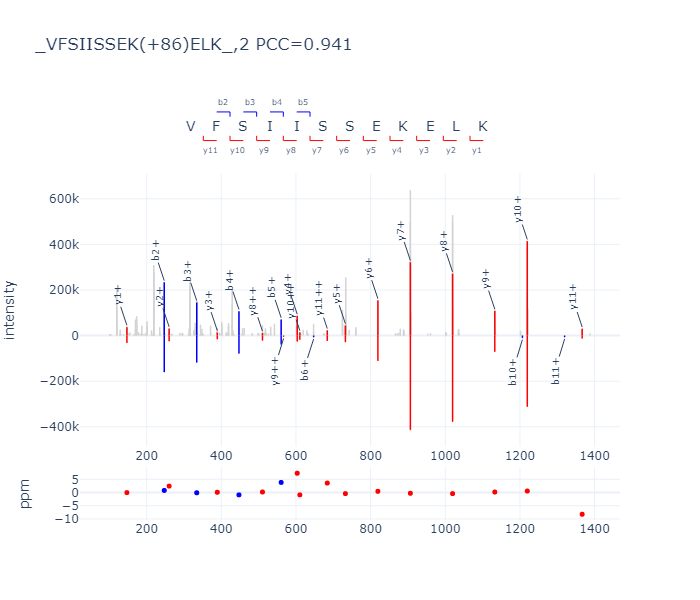

Supplement: Supplementary file 6 — Supplementary Data 3 [file 41467_2022_34904_MOESM6_ESM.zip › mirror-ms2-21ptm/Kmod_Hydroxy/_VFSIISSEK(+86)ELK_charge=2_nce=25_transfer_pcc=0.94.png]

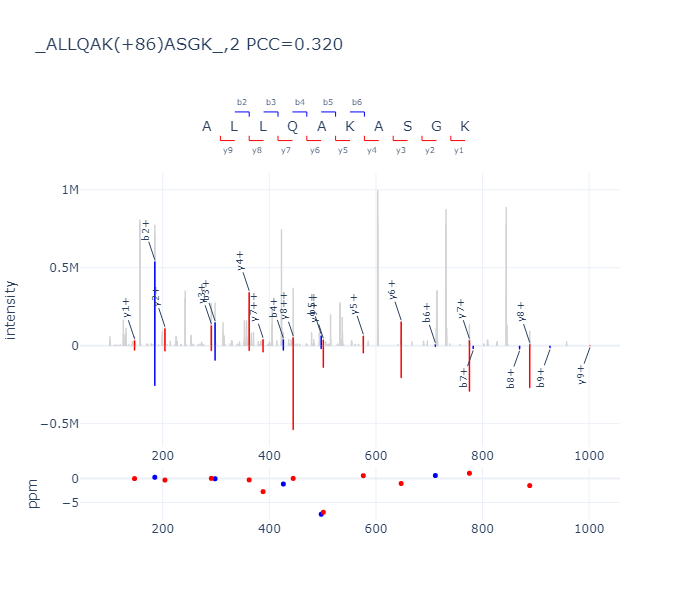

Supplement: Supplementary file 6 — Supplementary Data 3 [file 41467_2022_34904_MOESM6_ESM.zip › mirror-ms2-21ptm/Kmod_Malonyl/_ALLQAK(+86)ASGK_charge=2_nce=25_pretrain_pcc=0.32.png]

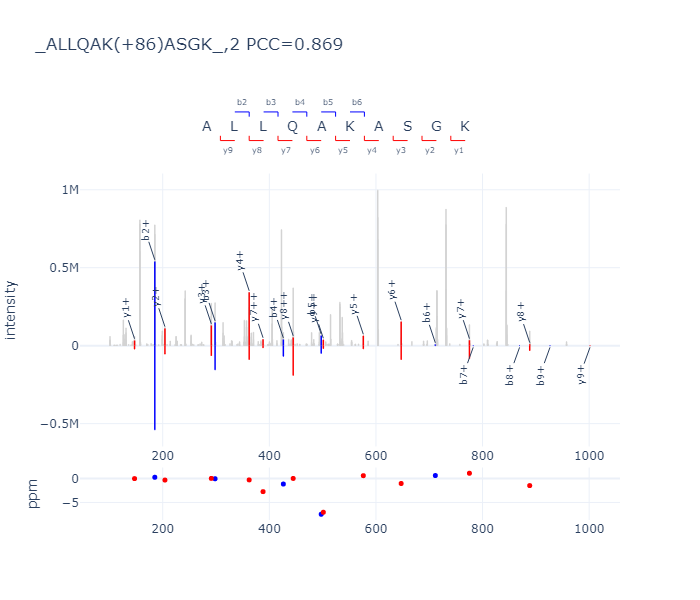

Supplement: Supplementary file 6 — Supplementary Data 3 [file 41467_2022_34904_MOESM6_ESM.zip › mirror-ms2-21ptm/Kmod_Malonyl/_ALLQAK(+86)ASGK_charge=2_nce=25_transfer_pcc=0.87.png]

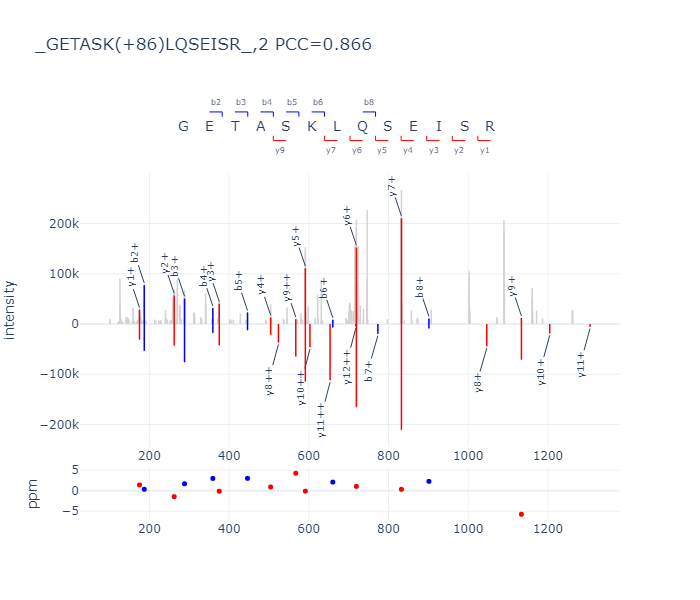

Supplement: Supplementary file 6 — Supplementary Data 3 [file 41467_2022_34904_MOESM6_ESM.zip › mirror-ms2-21ptm/Kmod_Malonyl/_GETASK(+86)LQSEISR_charge=2_nce=30_pretrain_pcc=0.87.png]

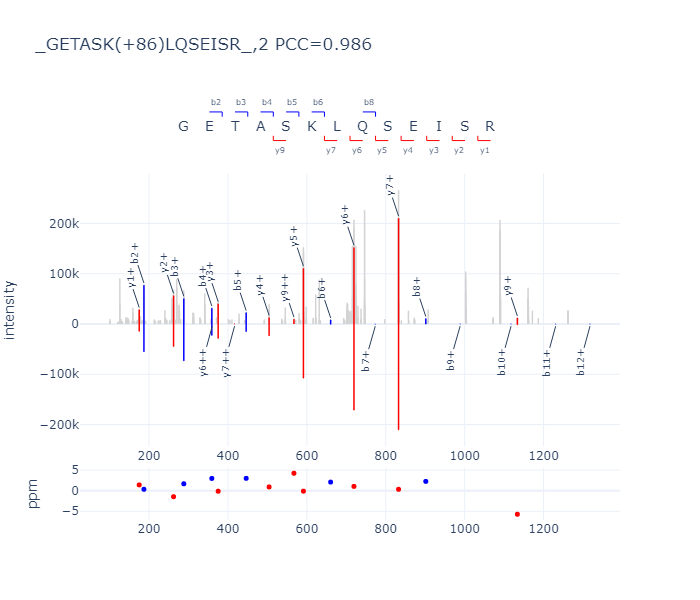

Supplement: Supplementary file 6 — Supplementary Data 3 [file 41467_2022_34904_MOESM6_ESM.zip › mirror-ms2-21ptm/Kmod_Malonyl/_GETASK(+86)LQSEISR_charge=2_nce=30_transfer_pcc=0.99.png]

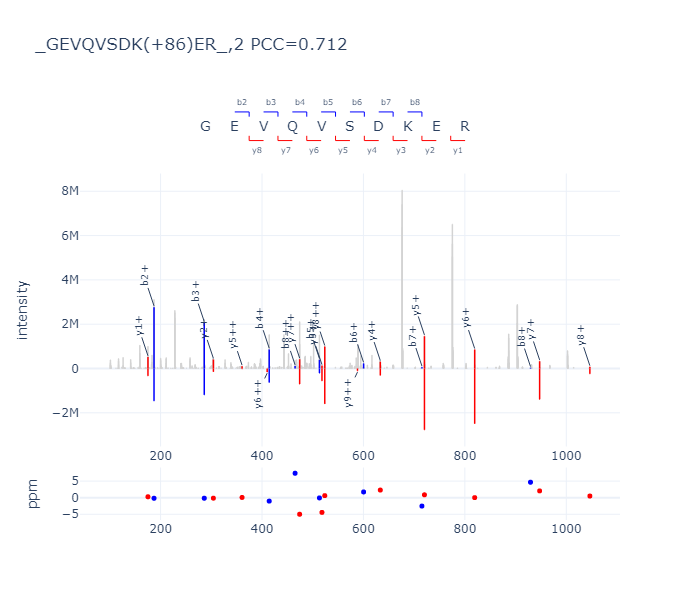

Supplement: Supplementary file 6 — Supplementary Data 3 [file 41467_2022_34904_MOESM6_ESM.zip › mirror-ms2-21ptm/Kmod_Malonyl/_GEVQVSDK(+86)ER_charge=2_nce=30_pretrain_pcc=0.71.png]

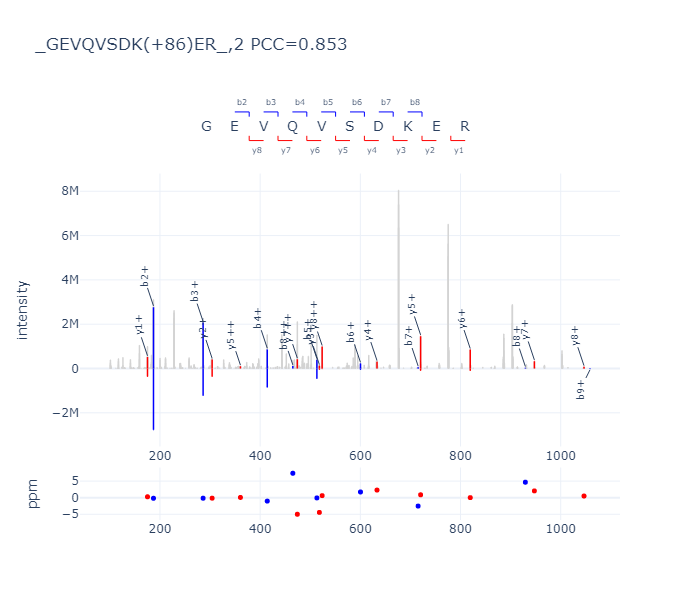

Supplement: Supplementary file 6 — Supplementary Data 3 [file 41467_2022_34904_MOESM6_ESM.zip › mirror-ms2-21ptm/Kmod_Malonyl/_GEVQVSDK(+86)ER_charge=2_nce=30_transfer_pcc=0.85.png]

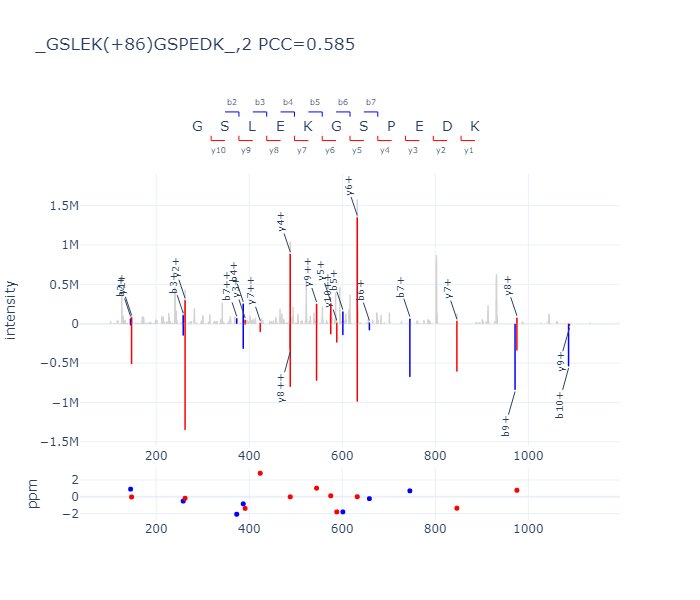

Supplement: Supplementary file 6 — Supplementary Data 3 [file 41467_2022_34904_MOESM6_ESM.zip › mirror-ms2-21ptm/Kmod_Malonyl/_GSLEK(+86)GSPEDK_charge=2_nce=25_pretrain_pcc=0.59.png]

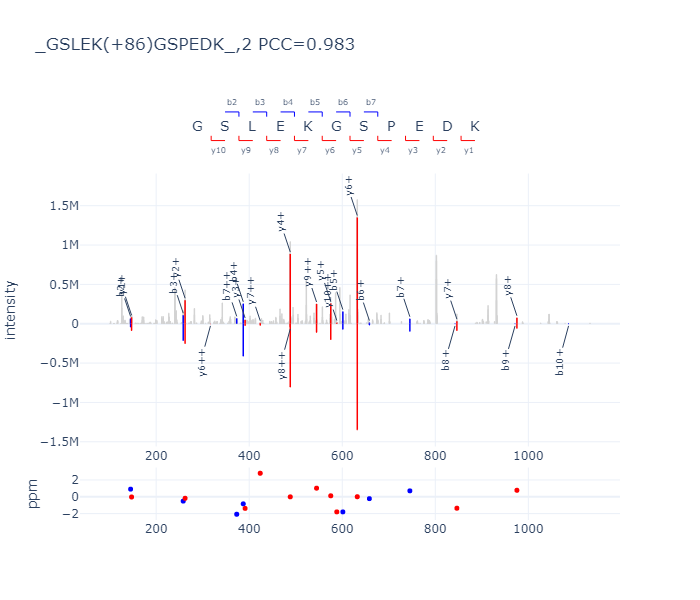

Supplement: Supplementary file 6 — Supplementary Data 3 [file 41467_2022_34904_MOESM6_ESM.zip › mirror-ms2-21ptm/Kmod_Malonyl/_GSLEK(+86)GSPEDK_charge=2_nce=25_transfer_pcc=0.98.png]

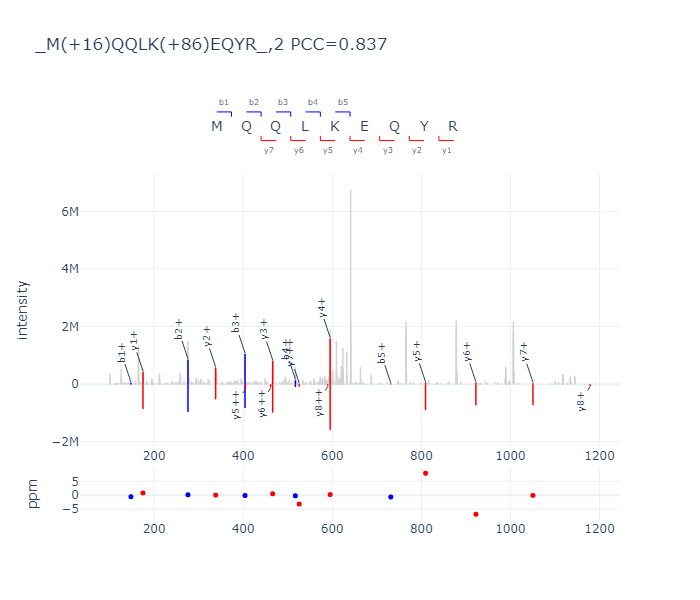

Supplement: Supplementary file 6 — Supplementary Data 3 [file 41467_2022_34904_MOESM6_ESM.zip › mirror-ms2-21ptm/Kmod_Malonyl/_M(+16)QQLK(+86)EQYR_charge=2_nce=35_pretrain_pcc=0.84.png]

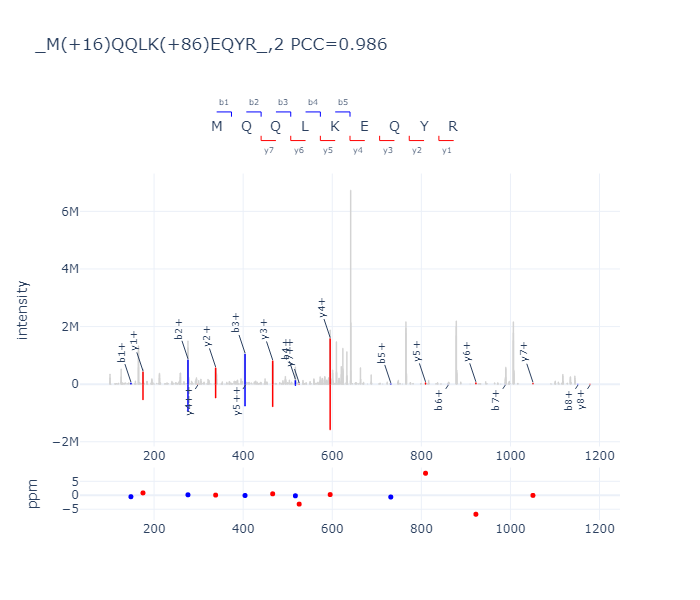

Supplement: Supplementary file 6 — Supplementary Data 3 [file 41467_2022_34904_MOESM6_ESM.zip › mirror-ms2-21ptm/Kmod_Malonyl/_M(+16)QQLK(+86)EQYR_charge=2_nce=35_transfer_pcc=0.99.png]

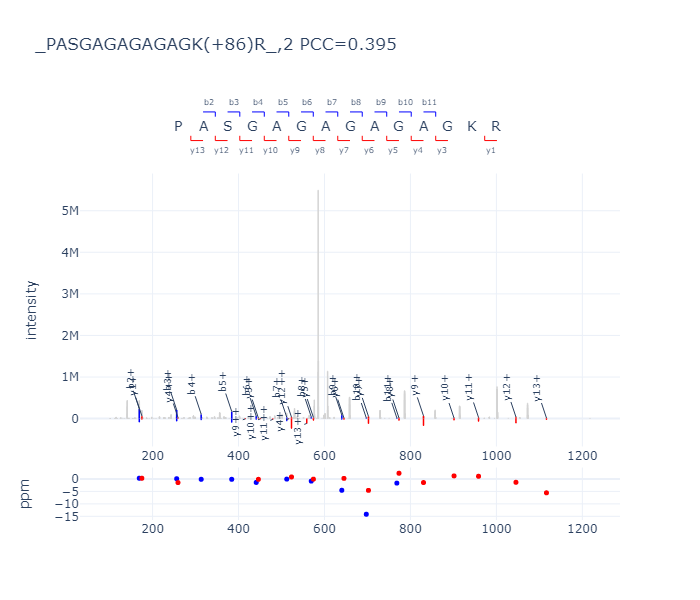

Supplement: Supplementary file 6 — Supplementary Data 3 [file 41467_2022_34904_MOESM6_ESM.zip › mirror-ms2-21ptm/Kmod_Malonyl/_PASGAGAGAGAGK(+86)R_charge=2_nce=25_pretrain_pcc=0.39.png]

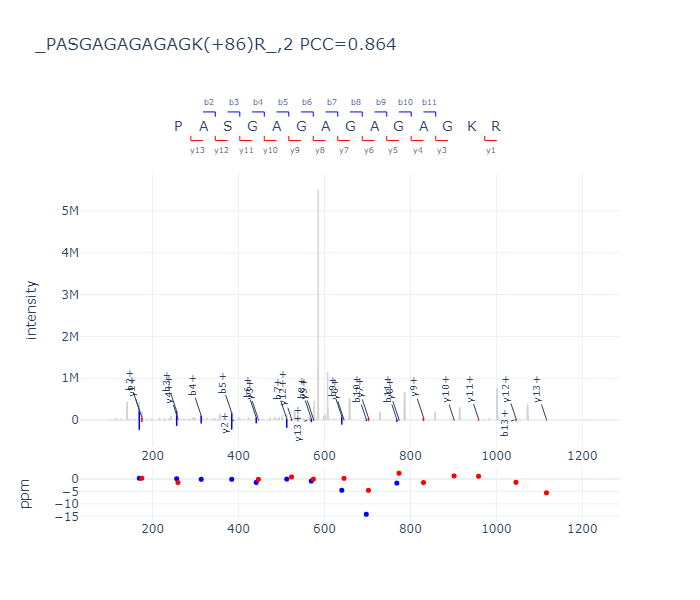

Supplement: Supplementary file 6 — Supplementary Data 3 [file 41467_2022_34904_MOESM6_ESM.zip › mirror-ms2-21ptm/Kmod_Malonyl/_PASGAGAGAGAGK(+86)R_charge=2_nce=25_transfer_pcc=0.86.png]

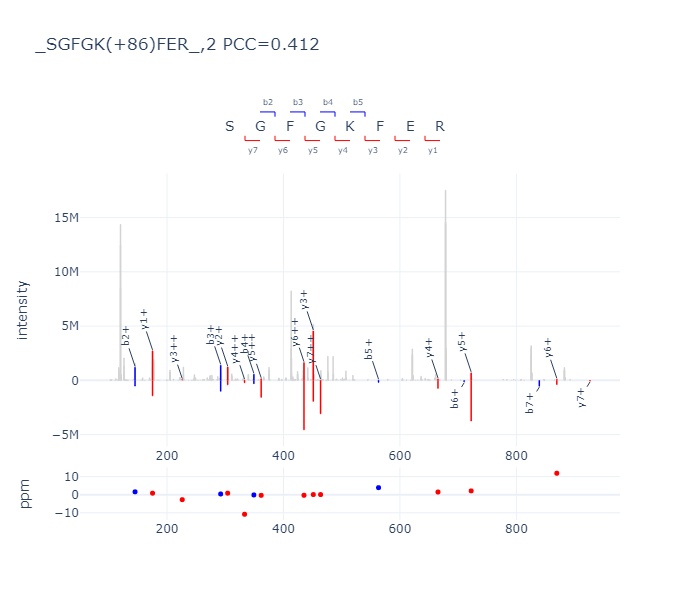

Supplement: Supplementary file 6 — Supplementary Data 3 [file 41467_2022_34904_MOESM6_ESM.zip › mirror-ms2-21ptm/Kmod_Malonyl/_SGFGK(+86)FER_charge=2_nce=25_pretrain_pcc=0.41.png]

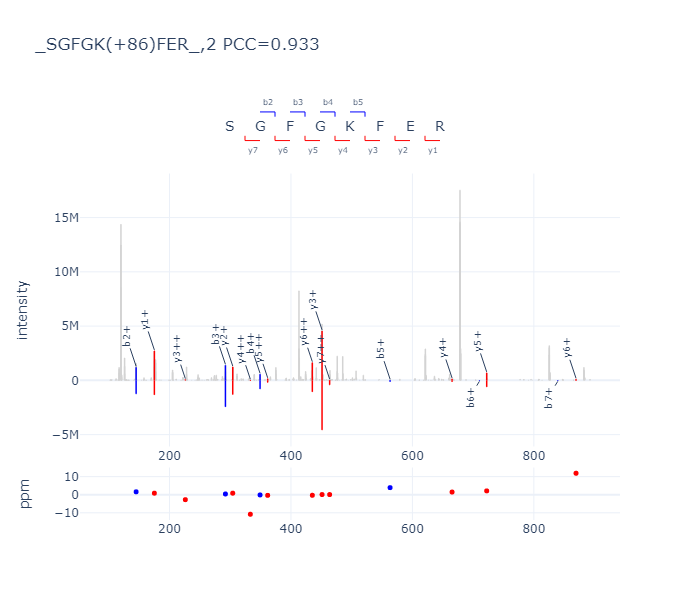

Supplement: Supplementary file 6 — Supplementary Data 3 [file 41467_2022_34904_MOESM6_ESM.zip › mirror-ms2-21ptm/Kmod_Malonyl/_SGFGK(+86)FER_charge=2_nce=25_transfer_pcc=0.93.png]

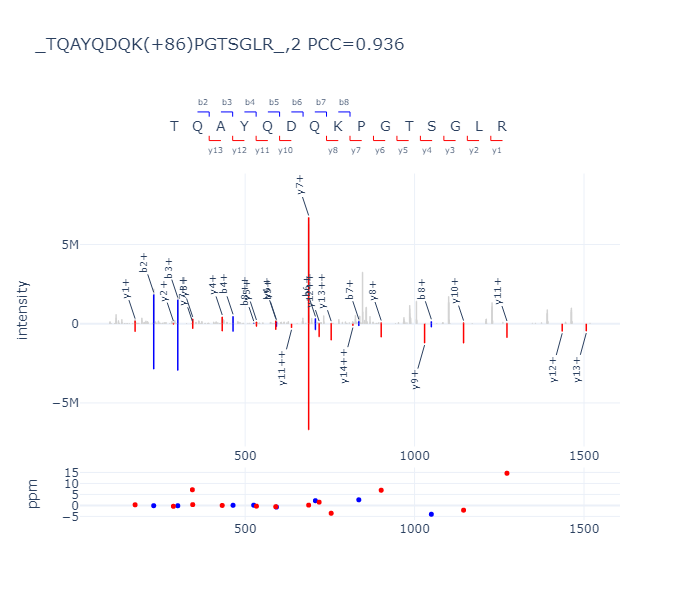

Supplement: Supplementary file 6 — Supplementary Data 3 [file 41467_2022_34904_MOESM6_ESM.zip › mirror-ms2-21ptm/Kmod_Malonyl/_TQAYQDQK(+86)PGTSGLR_charge=2_nce=30_pretrain_pcc=0.94.png]

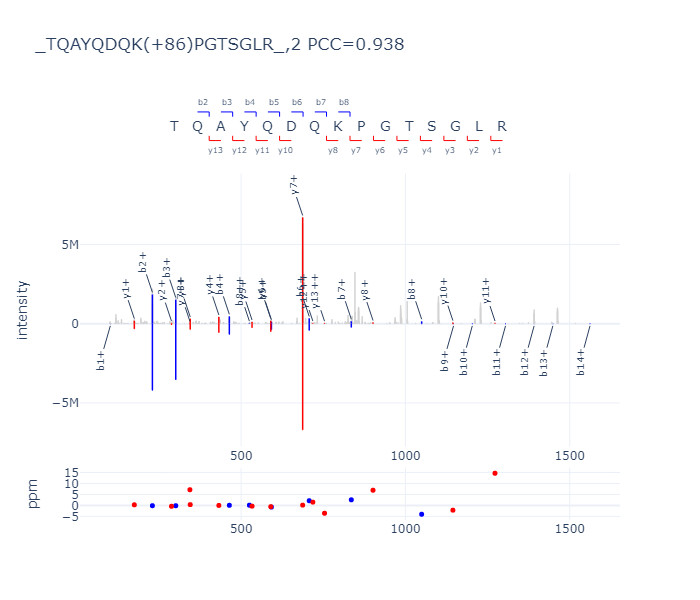

Supplement: Supplementary file 6 — Supplementary Data 3 [file 41467_2022_34904_MOESM6_ESM.zip › mirror-ms2-21ptm/Kmod_Malonyl/_TQAYQDQK(+86)PGTSGLR_charge=2_nce=30_transfer_pcc=0.94.png]

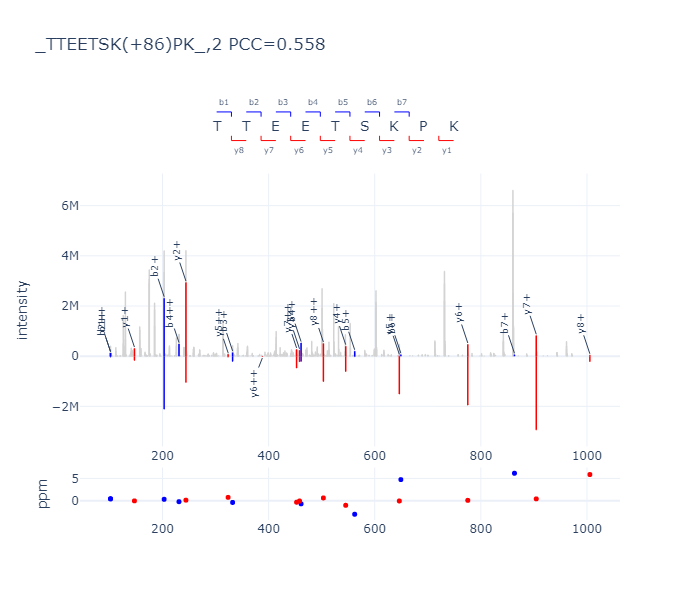

Supplement: Supplementary file 6 — Supplementary Data 3 [file 41467_2022_34904_MOESM6_ESM.zip › mirror-ms2-21ptm/Kmod_Malonyl/_TTEETSK(+86)PK_charge=2_nce=30_pretrain_pcc=0.56.png]

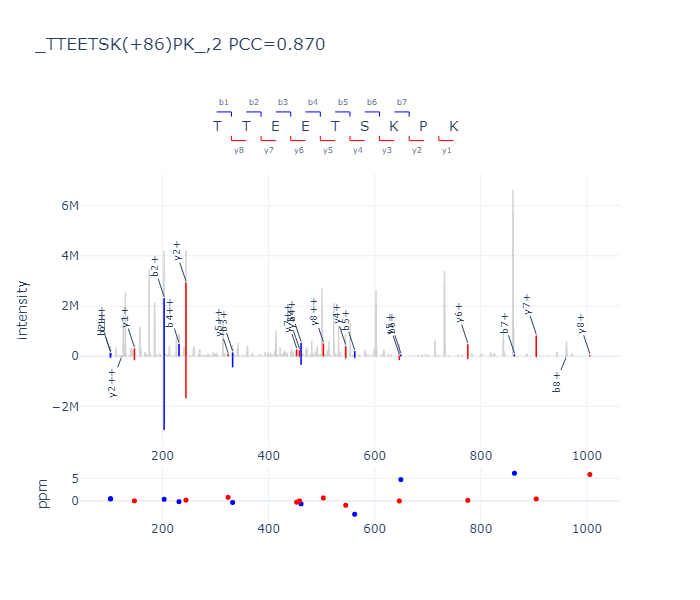

Supplement: Supplementary file 6 — Supplementary Data 3 [file 41467_2022_34904_MOESM6_ESM.zip › mirror-ms2-21ptm/Kmod_Malonyl/_TTEETSK(+86)PK_charge=2_nce=30_transfer_pcc=0.87.png]

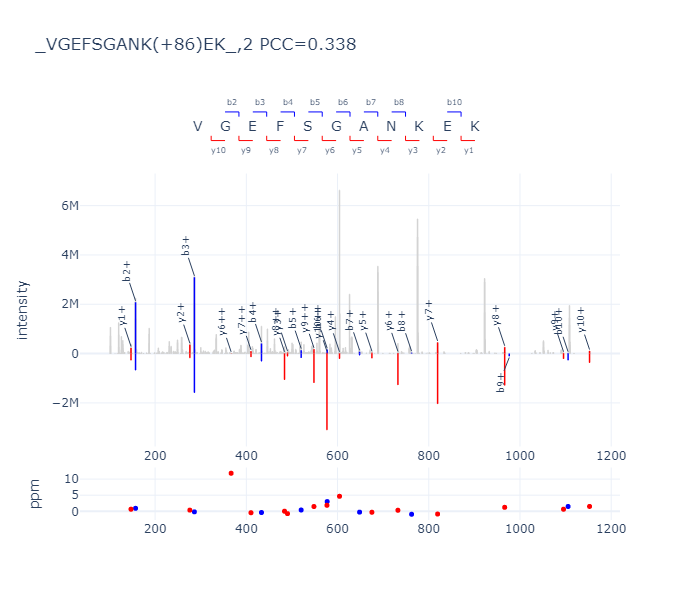

Supplement: Supplementary file 6 — Supplementary Data 3 [file 41467_2022_34904_MOESM6_ESM.zip › mirror-ms2-21ptm/Kmod_Malonyl/_VGEFSGANK(+86)EK_charge=2_nce=25_pretrain_pcc=0.34.png]

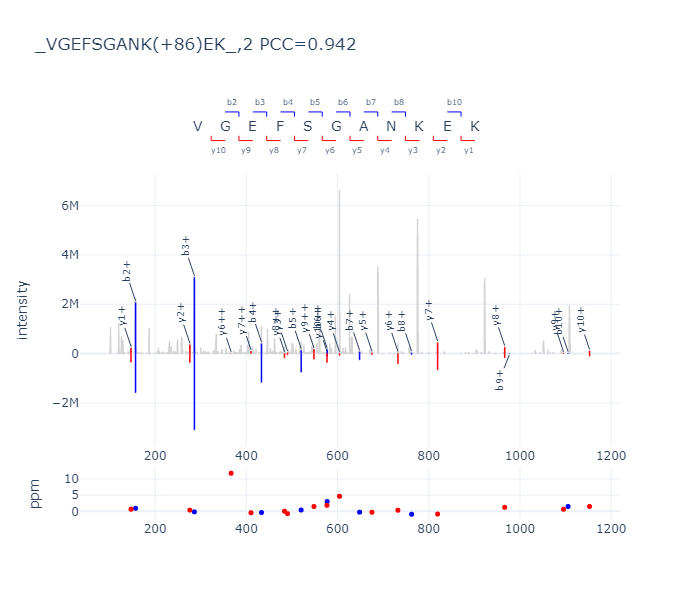

Supplement: Supplementary file 6 — Supplementary Data 3 [file 41467_2022_34904_MOESM6_ESM.zip › mirror-ms2-21ptm/Kmod_Malonyl/_VGEFSGANK(+86)EK_charge=2_nce=25_transfer_pcc=0.94.png]

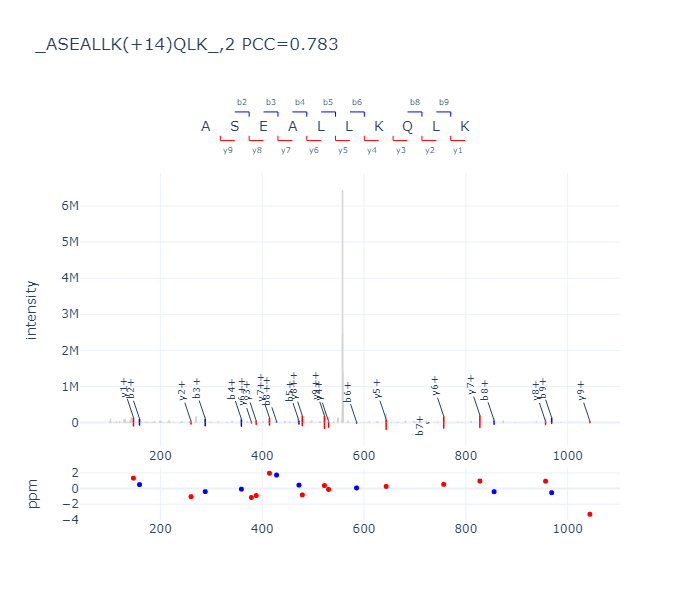

Supplement: Supplementary file 6 — Supplementary Data 3 [file 41467_2022_34904_MOESM6_ESM.zip › mirror-ms2-21ptm/Kmod_Methyl/_ASEALLK(+14)QLK_charge=2_nce=30_pretrain_pcc=0.78.png]

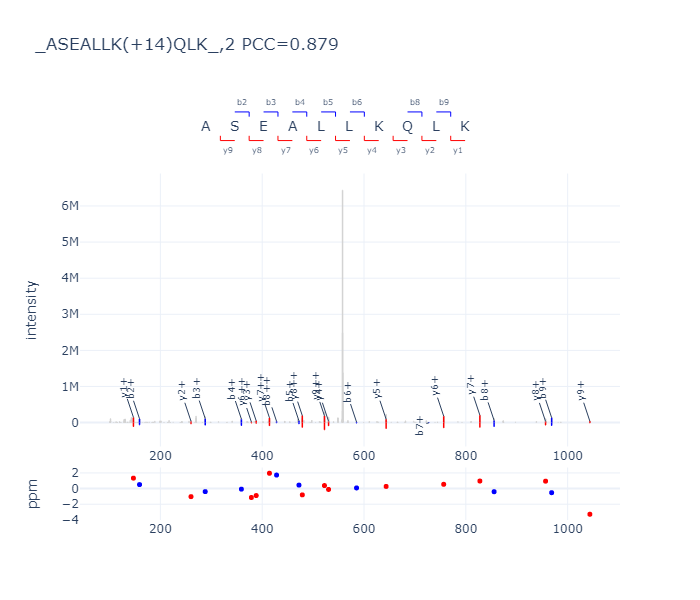

Supplement: Supplementary file 6 — Supplementary Data 3 [file 41467_2022_34904_MOESM6_ESM.zip › mirror-ms2-21ptm/Kmod_Methyl/_ASEALLK(+14)QLK_charge=2_nce=30_transfer_pcc=0.88.png]

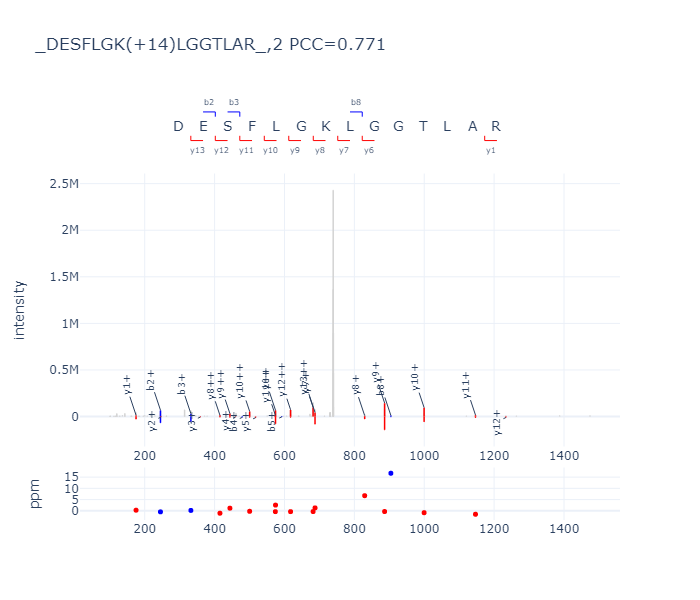

Supplement: Supplementary file 6 — Supplementary Data 3 [file 41467_2022_34904_MOESM6_ESM.zip › mirror-ms2-21ptm/Kmod_Methyl/_DESFLGK(+14)LGGTLAR_charge=2_nce=35_pretrain_pcc=0.77.png]

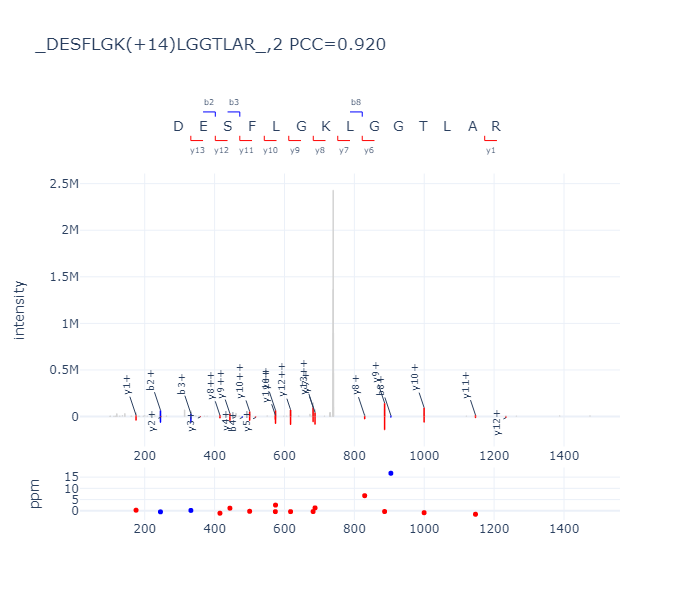

Supplement: Supplementary file 6 — Supplementary Data 3 [file 41467_2022_34904_MOESM6_ESM.zip › mirror-ms2-21ptm/Kmod_Methyl/_DESFLGK(+14)LGGTLAR_charge=2_nce=35_transfer_pcc=0.92.png]

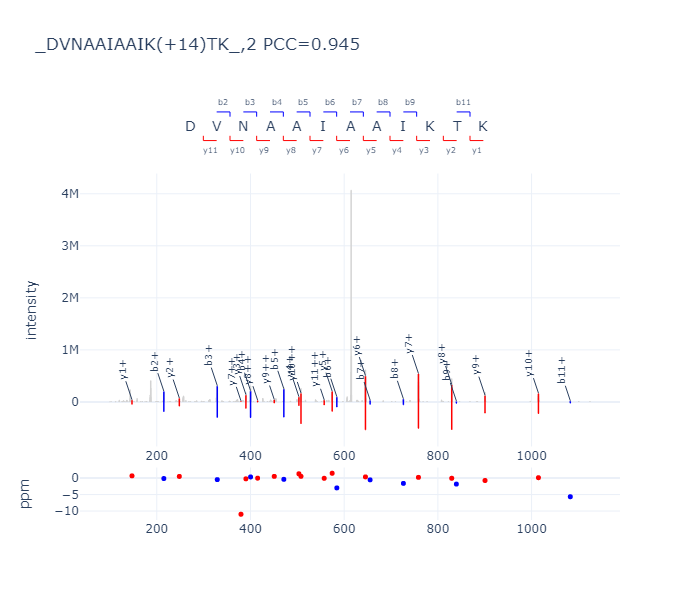

Supplement: Supplementary file 6 — Supplementary Data 3 [file 41467_2022_34904_MOESM6_ESM.zip › mirror-ms2-21ptm/Kmod_Methyl/_DVNAAIAAIK(+14)TK_charge=2_nce=25_pretrain_pcc=0.94.png]

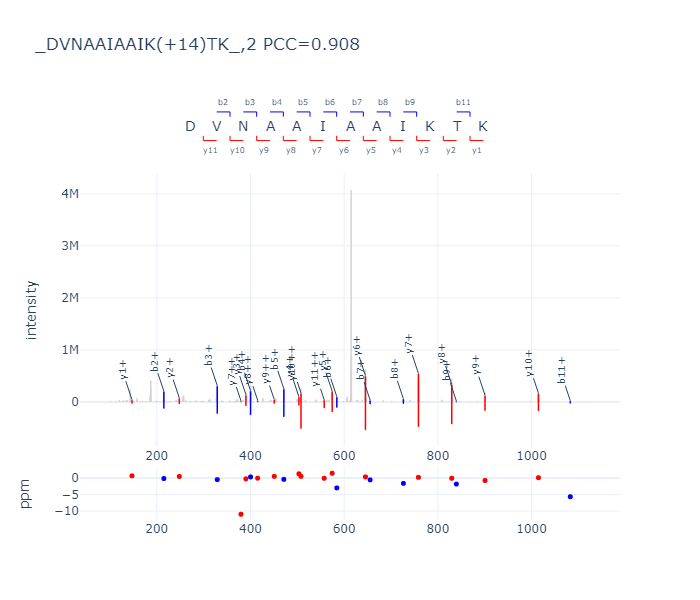

Supplement: Supplementary file 6 — Supplementary Data 3 [file 41467_2022_34904_MOESM6_ESM.zip › mirror-ms2-21ptm/Kmod_Methyl/_DVNAAIAAIK(+14)TK_charge=2_nce=25_transfer_pcc=0.91.png]

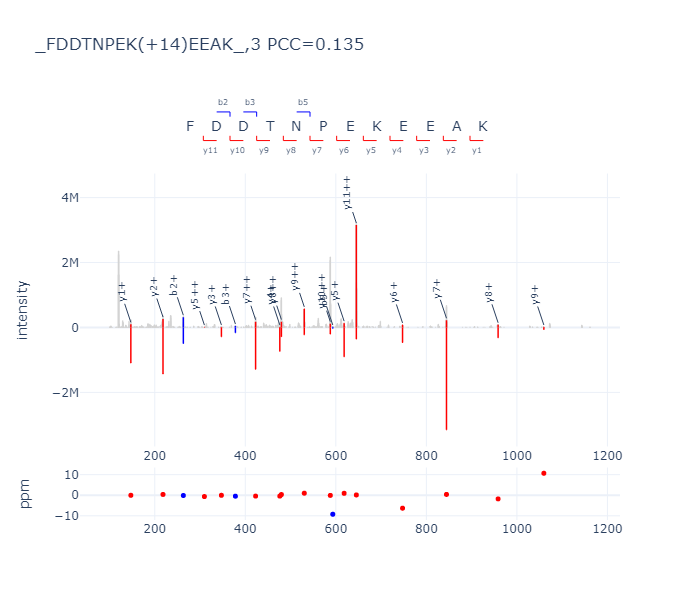

Supplement: Supplementary file 6 — Supplementary Data 3 [file 41467_2022_34904_MOESM6_ESM.zip › mirror-ms2-21ptm/Kmod_Methyl/_FDDTNPEK(+14)EEAK_charge=3_nce=35_pretrain_pcc=0.13.png]

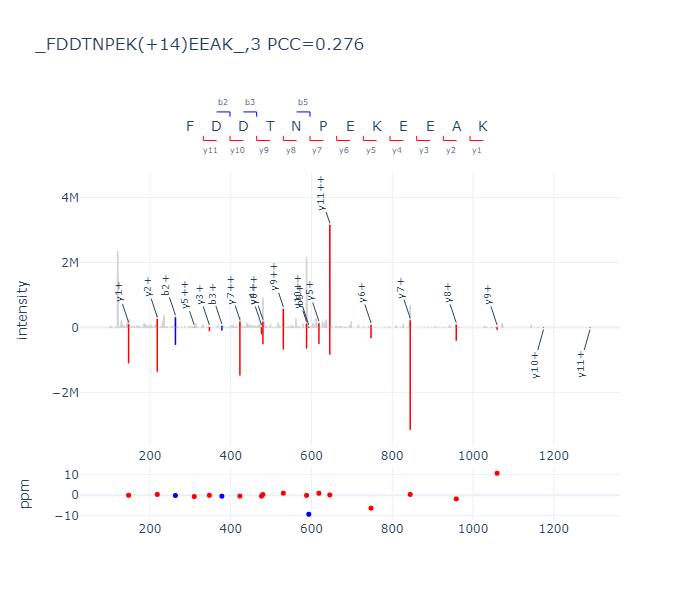

Supplement: Supplementary file 6 — Supplementary Data 3 [file 41467_2022_34904_MOESM6_ESM.zip › mirror-ms2-21ptm/Kmod_Methyl/_FDDTNPEK(+14)EEAK_charge=3_nce=35_transfer_pcc=0.28.png]

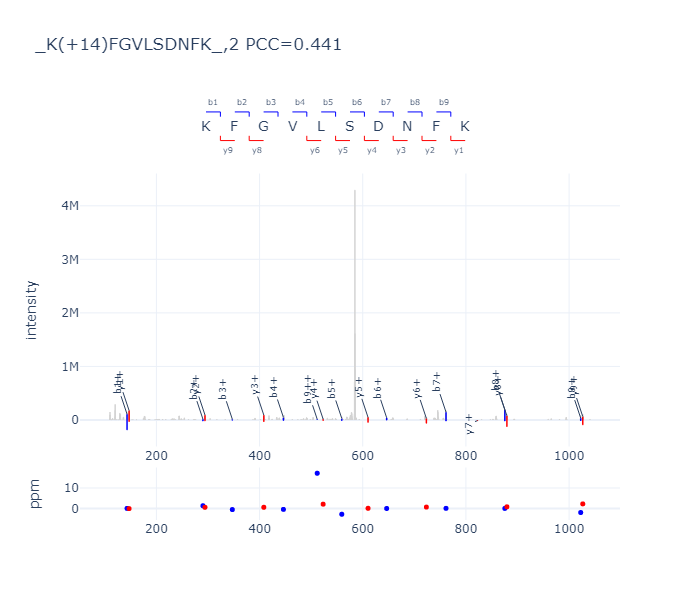

Supplement: Supplementary file 6 — Supplementary Data 3 [file 41467_2022_34904_MOESM6_ESM.zip › mirror-ms2-21ptm/Kmod_Methyl/_K(+14)FGVLSDNFK_charge=2_nce=30_pretrain_pcc=0.44.png]

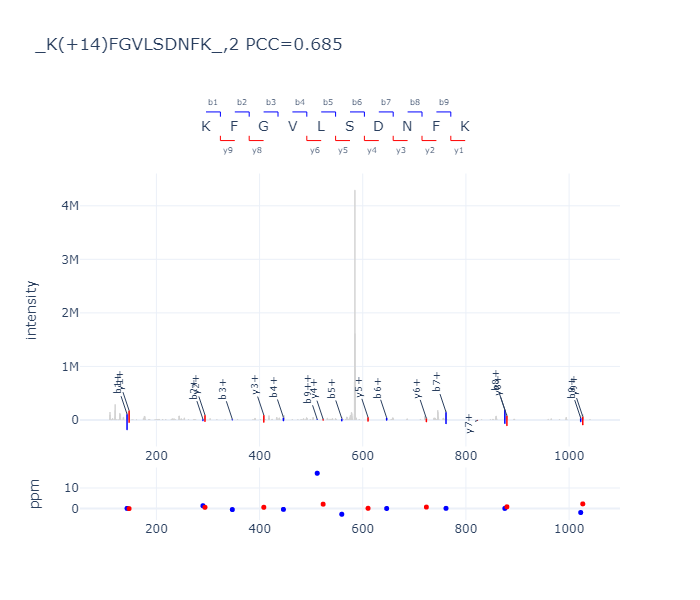

Supplement: Supplementary file 6 — Supplementary Data 3 [file 41467_2022_34904_MOESM6_ESM.zip › mirror-ms2-21ptm/Kmod_Methyl/_K(+14)FGVLSDNFK_charge=2_nce=30_transfer_pcc=0.69.png]

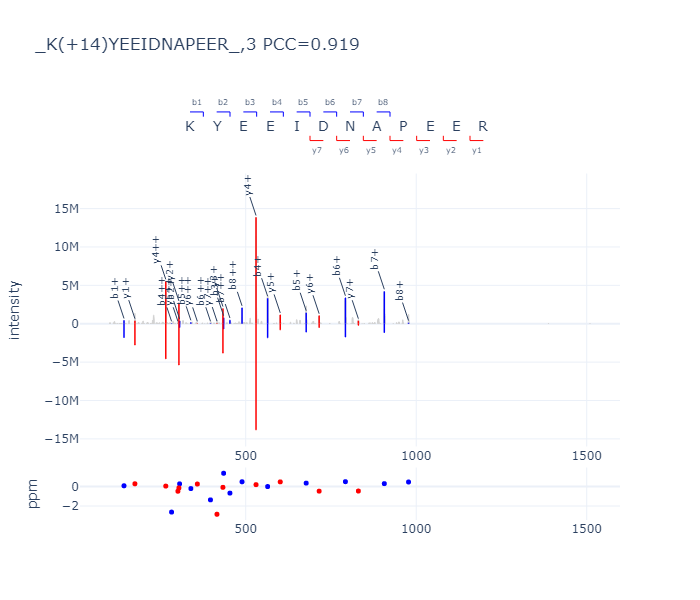

Supplement: Supplementary file 6 — Supplementary Data 3 [file 41467_2022_34904_MOESM6_ESM.zip › mirror-ms2-21ptm/Kmod_Methyl/_K(+14)YEEIDNAPEER_charge=3_nce=35_pretrain_pcc=0.92.png]

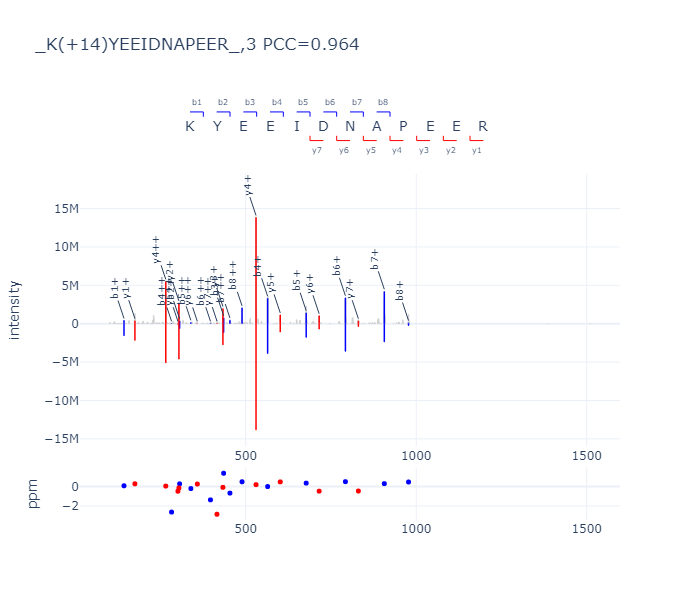

Supplement: Supplementary file 6 — Supplementary Data 3 [file 41467_2022_34904_MOESM6_ESM.zip › mirror-ms2-21ptm/Kmod_Methyl/_K(+14)YEEIDNAPEER_charge=3_nce=35_transfer_pcc=0.96.png]

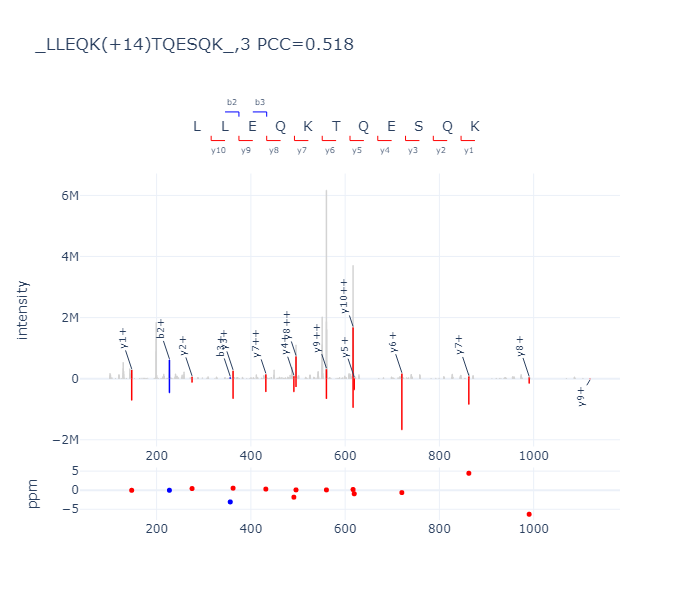

Supplement: Supplementary file 6 — Supplementary Data 3 [file 41467_2022_34904_MOESM6_ESM.zip › mirror-ms2-21ptm/Kmod_Methyl/_LLEQK(+14)TQESQK_charge=3_nce=30_pretrain_pcc=0.52.png]

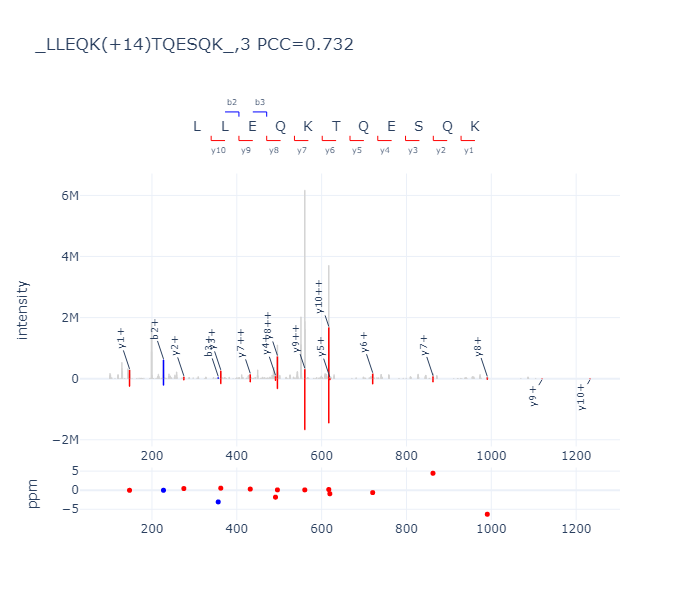

Supplement: Supplementary file 6 — Supplementary Data 3 [file 41467_2022_34904_MOESM6_ESM.zip › mirror-ms2-21ptm/Kmod_Methyl/_LLEQK(+14)TQESQK_charge=3_nce=30_transfer_pcc=0.73.png]

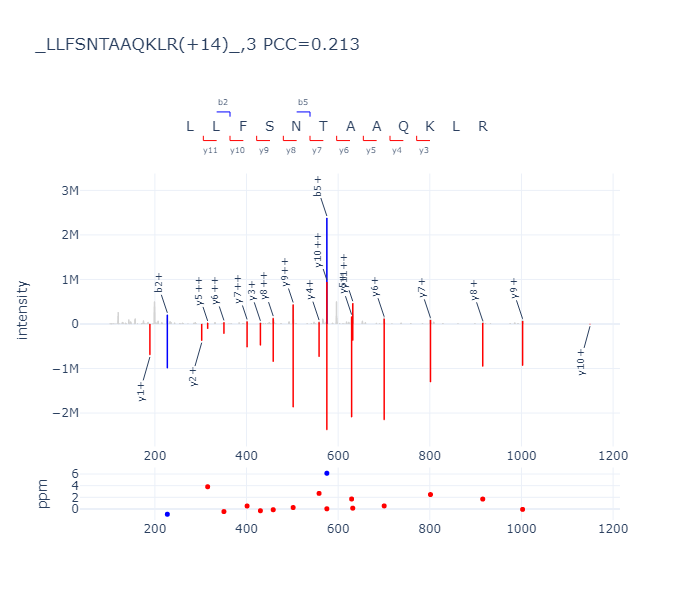

Supplement: Supplementary file 6 — Supplementary Data 3 [file 41467_2022_34904_MOESM6_ESM.zip › mirror-ms2-21ptm/Kmod_Methyl/_LLFSNTAAQKLR(+14)_charge=3_nce=30_pretrain_pcc=0.21.png]

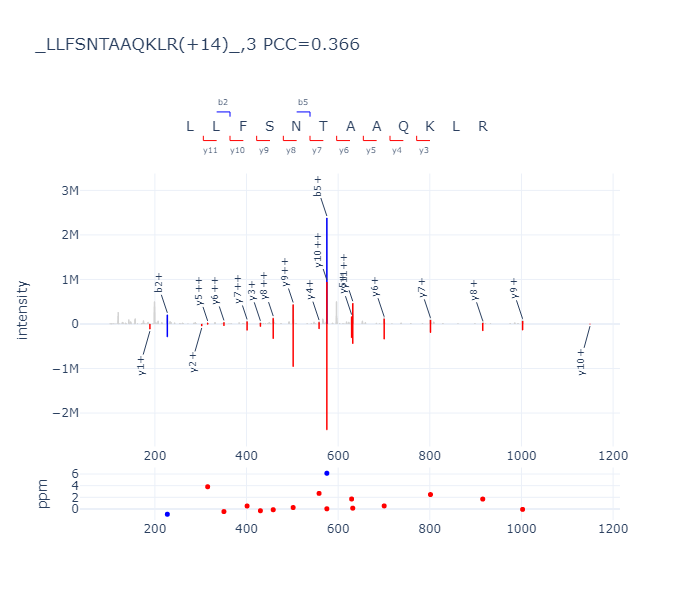

Supplement: Supplementary file 6 — Supplementary Data 3 [file 41467_2022_34904_MOESM6_ESM.zip › mirror-ms2-21ptm/Kmod_Methyl/_LLFSNTAAQKLR(+14)_charge=3_nce=30_transfer_pcc=0.37.png]

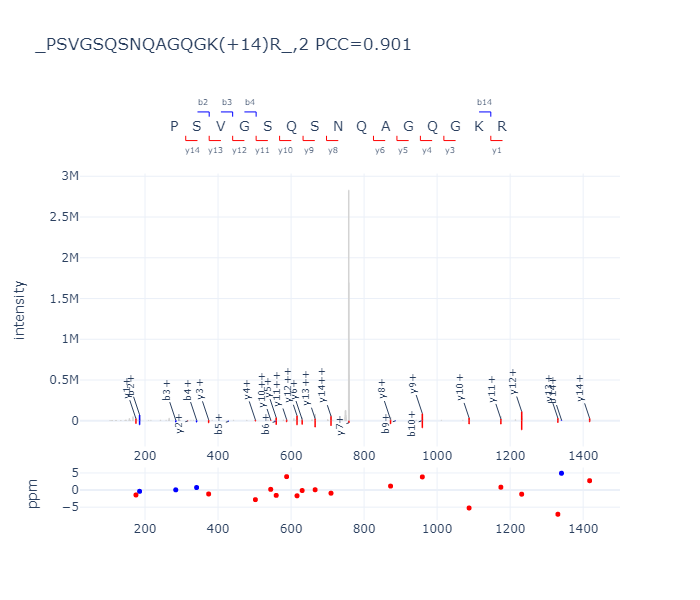

Supplement: Supplementary file 6 — Supplementary Data 3 [file 41467_2022_34904_MOESM6_ESM.zip › mirror-ms2-21ptm/Kmod_Methyl/_PSVGSQSNQAGQGK(+14)R_charge=2_nce=30_pretrain_pcc=0.90.png]

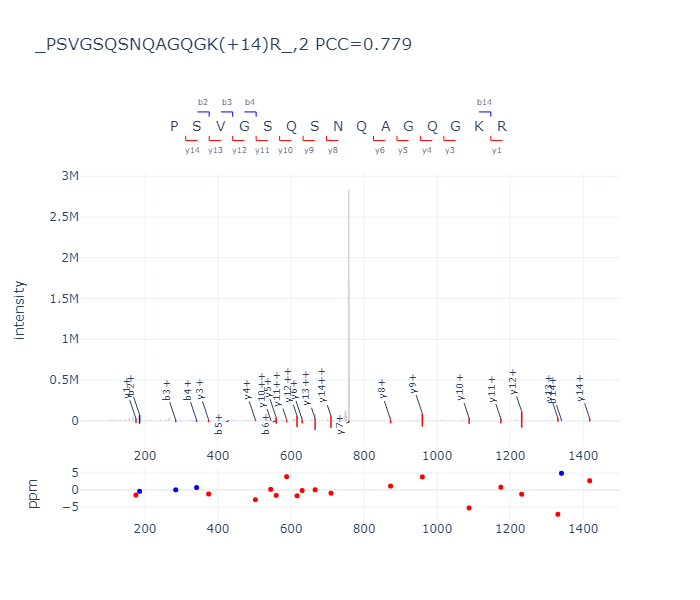

Supplement: Supplementary file 6 — Supplementary Data 3 [file 41467_2022_34904_MOESM6_ESM.zip › mirror-ms2-21ptm/Kmod_Methyl/_PSVGSQSNQAGQGK(+14)R_charge=2_nce=30_transfer_pcc=0.78.png]

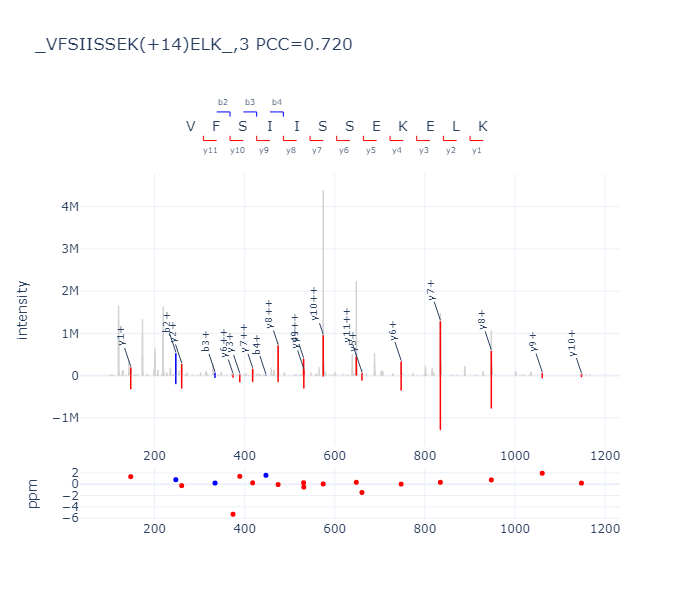

Supplement: Supplementary file 6 — Supplementary Data 3 [file 41467_2022_34904_MOESM6_ESM.zip › mirror-ms2-21ptm/Kmod_Methyl/_VFSIISSEK(+14)ELK_charge=3_nce=35_pretrain_pcc=0.72.png]

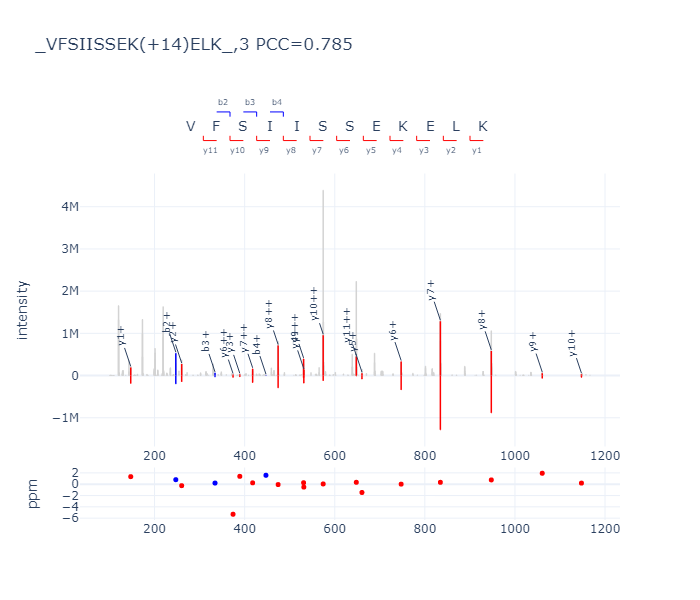

Supplement: Supplementary file 6 — Supplementary Data 3 [file 41467_2022_34904_MOESM6_ESM.zip › mirror-ms2-21ptm/Kmod_Methyl/_VFSIISSEK(+14)ELK_charge=3_nce=35_transfer_pcc=0.79.png]

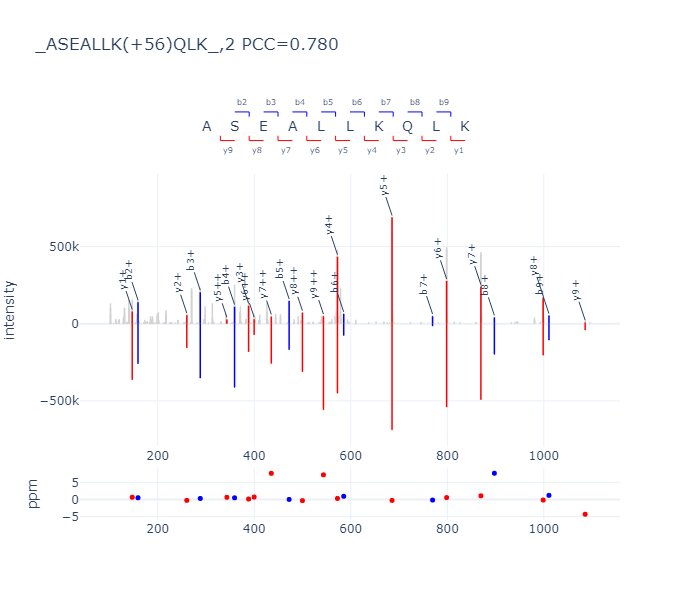

Supplement: Supplementary file 6 — Supplementary Data 3 [file 41467_2022_34904_MOESM6_ESM.zip › mirror-ms2-21ptm/Kmod_Propion/_ASEALLK(+56)QLK_charge=2_nce=30_pretrain_pcc=0.78.png]
