# Supplementary figures and images for: AlphaPeptDeep: a modular deep learning framework to predict peptide properties for proteomics (part 1 of 2)
Source: Nat Commun. 2022 Nov 24;13:7238. doi: 10.1038/s41467-022-34904-3 (PMC9700817; doi:10.1038/s41467-022-34904-3)

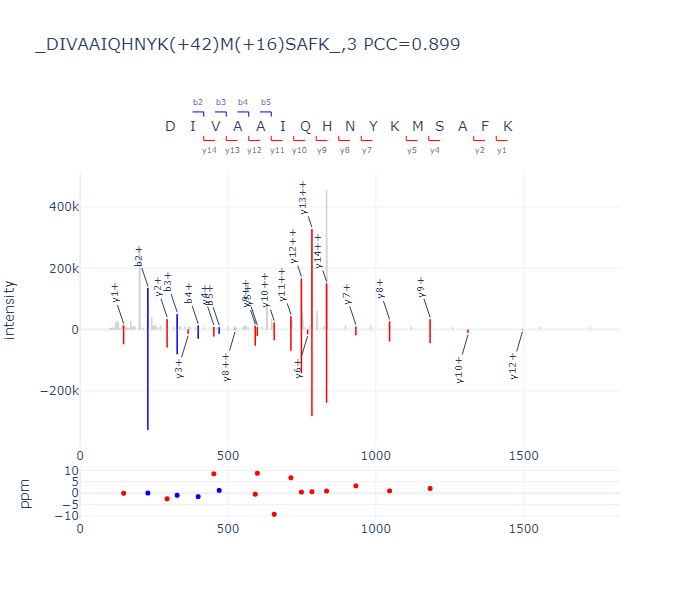

Supplement: Supplementary file 6 — Supplementary Data 3 [file 41467_2022_34904_MOESM6_ESM.zip › mirror-ms2-21ptm/Kmod_Acetyl/_DIVAAIQHNYK(+42)M(+16)SAFK_charge=3_nce=30_pretrain_pcc=0.90.png]

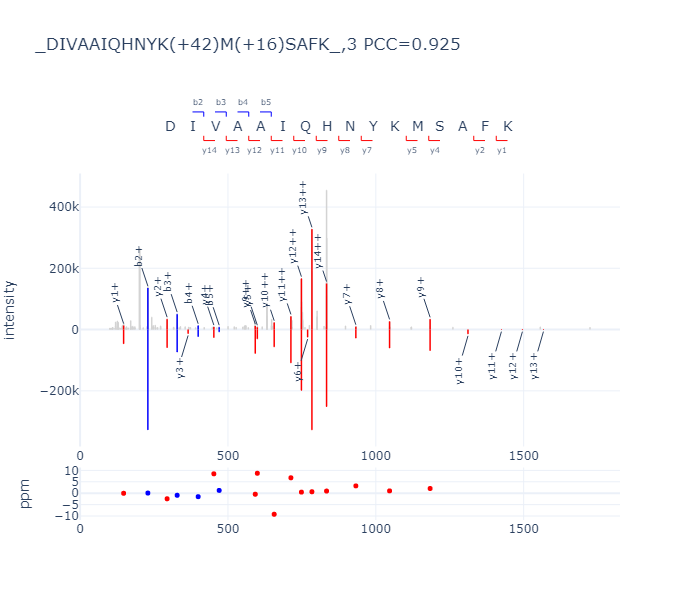

Supplement: Supplementary file 6 — Supplementary Data 3 [file 41467_2022_34904_MOESM6_ESM.zip › mirror-ms2-21ptm/Kmod_Acetyl/_DIVAAIQHNYK(+42)M(+16)SAFK_charge=3_nce=30_transfer_pcc=0.93.png]

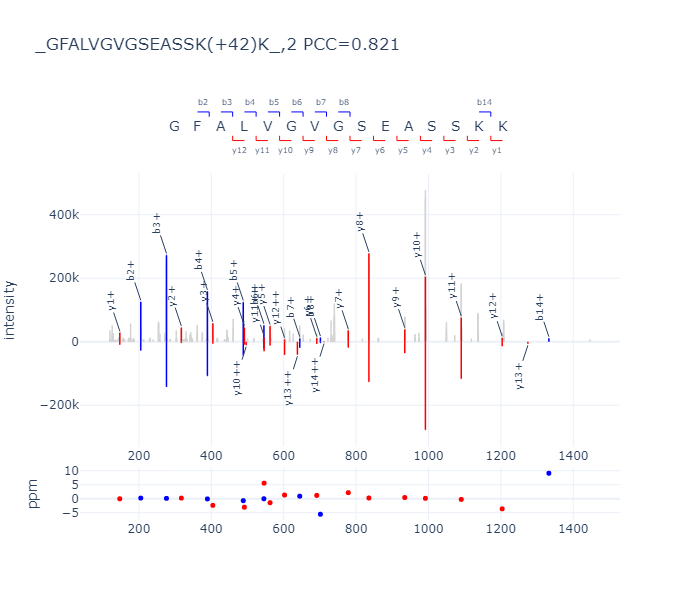

Supplement: Supplementary file 6 — Supplementary Data 3 [file 41467_2022_34904_MOESM6_ESM.zip › mirror-ms2-21ptm/Kmod_Acetyl/_GFALVGVGSEASSK(+42)K_charge=2_nce=25_pretrain_pcc=0.82.png]

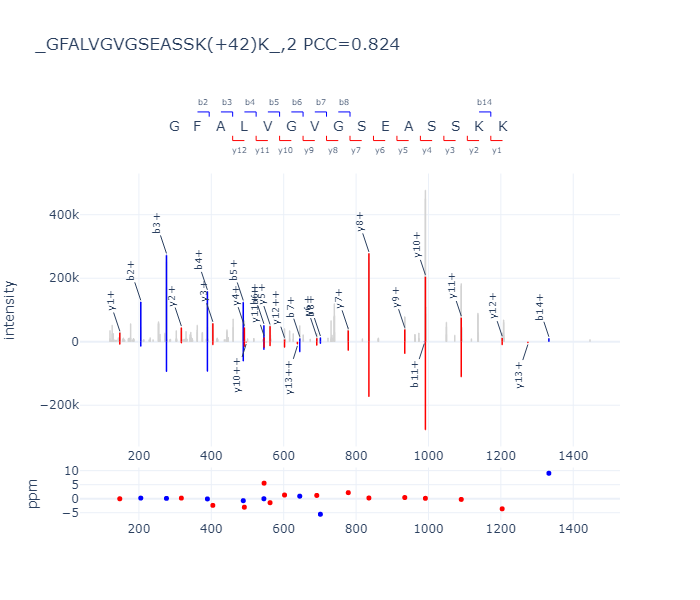

Supplement: Supplementary file 6 — Supplementary Data 3 [file 41467_2022_34904_MOESM6_ESM.zip › mirror-ms2-21ptm/Kmod_Acetyl/_GFALVGVGSEASSK(+42)K_charge=2_nce=25_transfer_pcc=0.82.png]

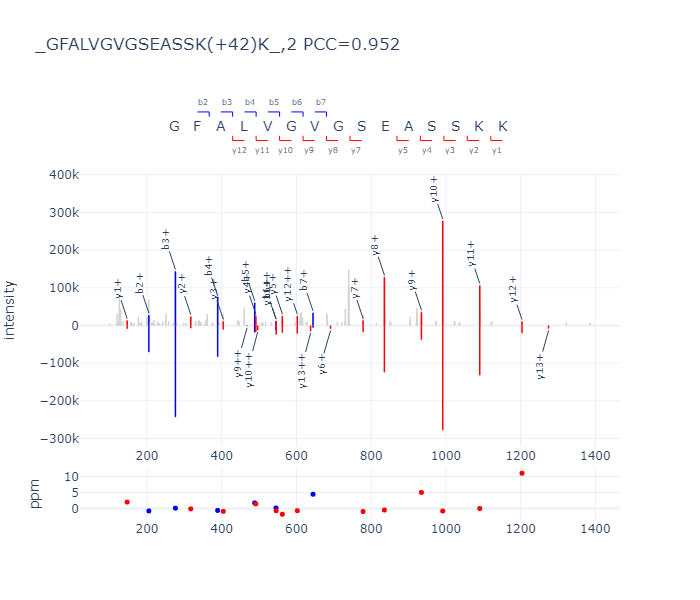

Supplement: Supplementary file 6 — Supplementary Data 3 [file 41467_2022_34904_MOESM6_ESM.zip › mirror-ms2-21ptm/Kmod_Acetyl/_GFALVGVGSEASSK(+42)K_charge=2_nce=30_pretrain_pcc=0.95.png]

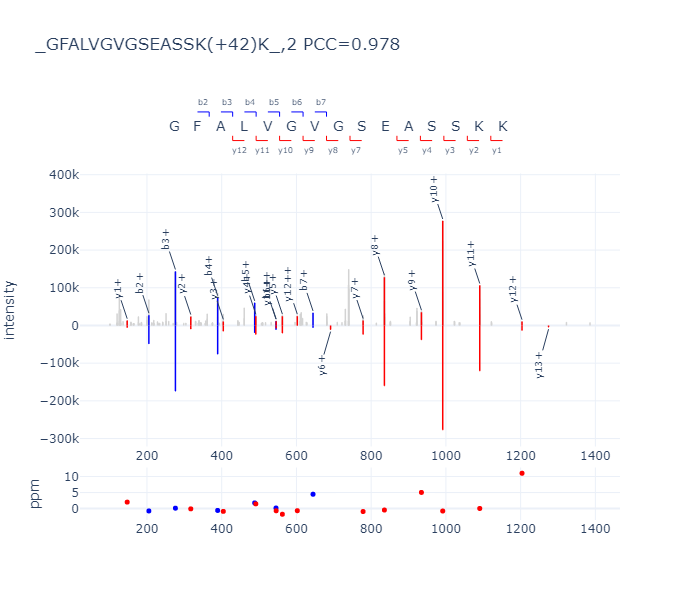

Supplement: Supplementary file 6 — Supplementary Data 3 [file 41467_2022_34904_MOESM6_ESM.zip › mirror-ms2-21ptm/Kmod_Acetyl/_GFALVGVGSEASSK(+42)K_charge=2_nce=30_transfer_pcc=0.98.png]

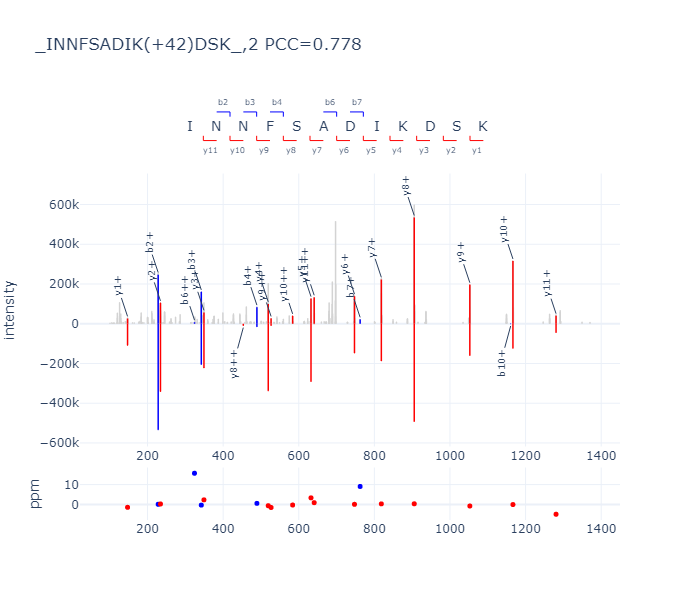

Supplement: Supplementary file 6 — Supplementary Data 3 [file 41467_2022_34904_MOESM6_ESM.zip › mirror-ms2-21ptm/Kmod_Acetyl/_INNFSADIK(+42)DSK_charge=2_nce=35_pretrain_pcc=0.78.png]

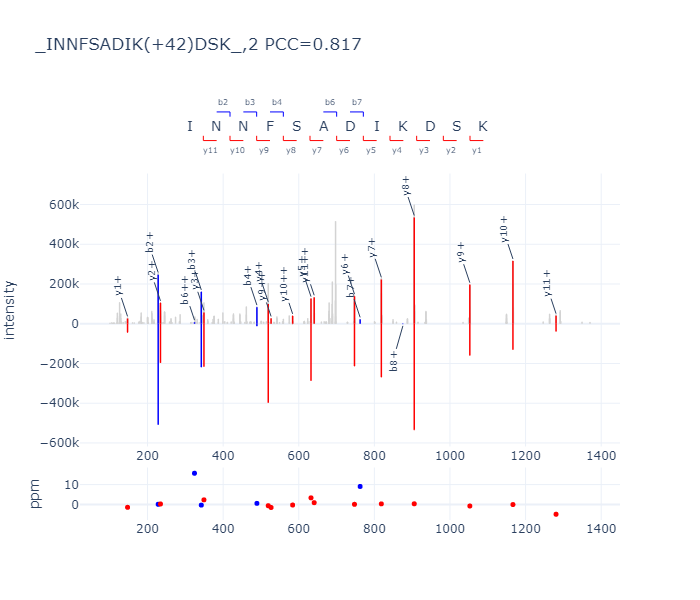

Supplement: Supplementary file 6 — Supplementary Data 3 [file 41467_2022_34904_MOESM6_ESM.zip › mirror-ms2-21ptm/Kmod_Acetyl/_INNFSADIK(+42)DSK_charge=2_nce=35_transfer_pcc=0.82.png]

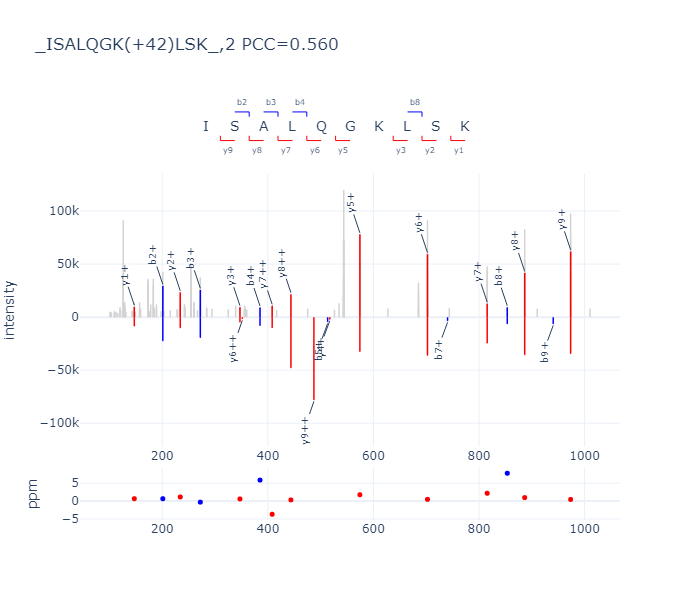

Supplement: Supplementary file 6 — Supplementary Data 3 [file 41467_2022_34904_MOESM6_ESM.zip › mirror-ms2-21ptm/Kmod_Acetyl/_ISALQGK(+42)LSK_charge=2_nce=25_pretrain_pcc=0.56.png]

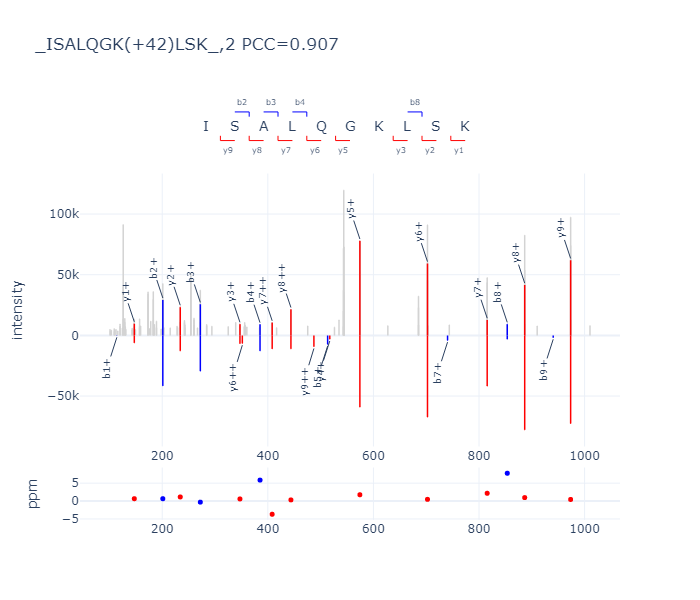

Supplement: Supplementary file 6 — Supplementary Data 3 [file 41467_2022_34904_MOESM6_ESM.zip › mirror-ms2-21ptm/Kmod_Acetyl/_ISALQGK(+42)LSK_charge=2_nce=25_transfer_pcc=0.91.png]

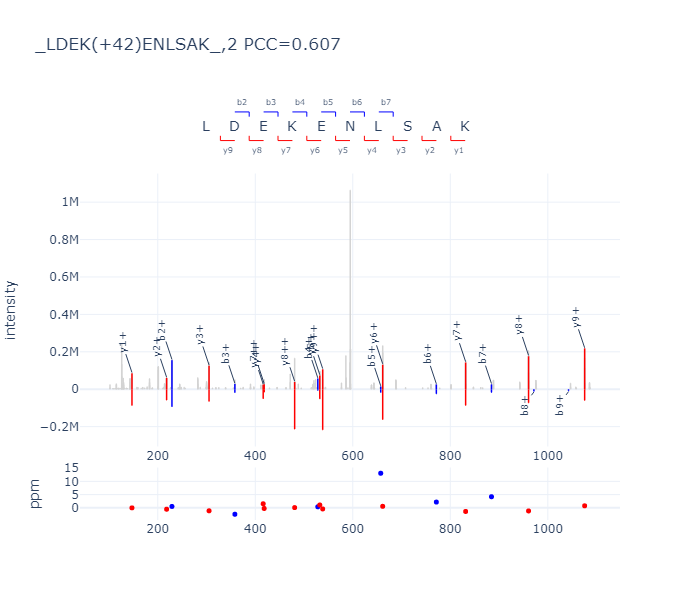

Supplement: Supplementary file 6 — Supplementary Data 3 [file 41467_2022_34904_MOESM6_ESM.zip › mirror-ms2-21ptm/Kmod_Acetyl/_LDEK(+42)ENLSAK_charge=2_nce=30_pretrain_pcc=0.61.png]

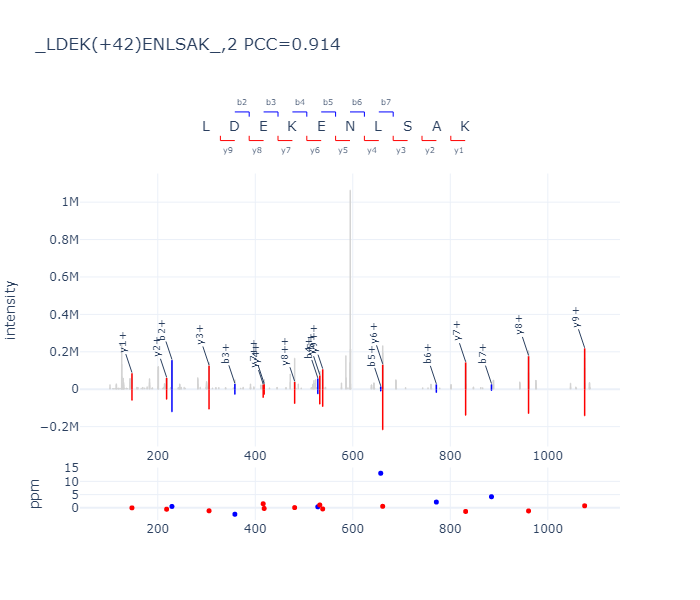

Supplement: Supplementary file 6 — Supplementary Data 3 [file 41467_2022_34904_MOESM6_ESM.zip › mirror-ms2-21ptm/Kmod_Acetyl/_LDEK(+42)ENLSAK_charge=2_nce=30_transfer_pcc=0.91.png]

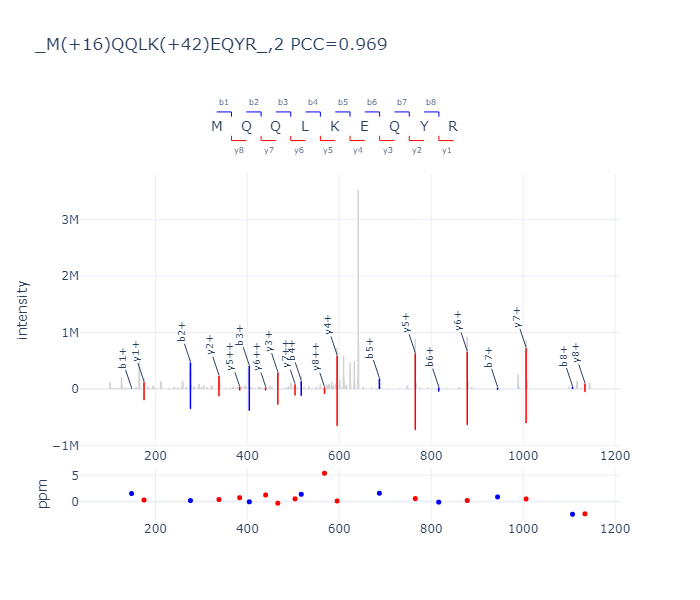

Supplement: Supplementary file 6 — Supplementary Data 3 [file 41467_2022_34904_MOESM6_ESM.zip › mirror-ms2-21ptm/Kmod_Acetyl/_M(+16)QQLK(+42)EQYR_charge=2_nce=30_pretrain_pcc=0.97.png]

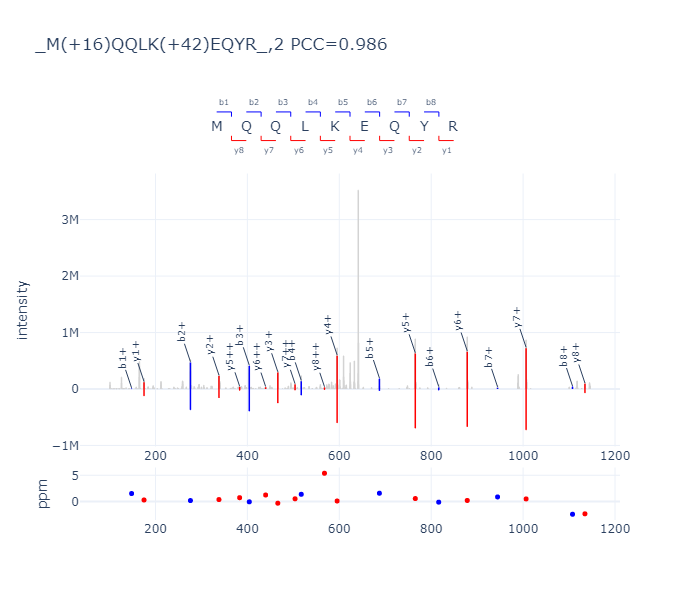

Supplement: Supplementary file 6 — Supplementary Data 3 [file 41467_2022_34904_MOESM6_ESM.zip › mirror-ms2-21ptm/Kmod_Acetyl/_M(+16)QQLK(+42)EQYR_charge=2_nce=30_transfer_pcc=0.99.png]

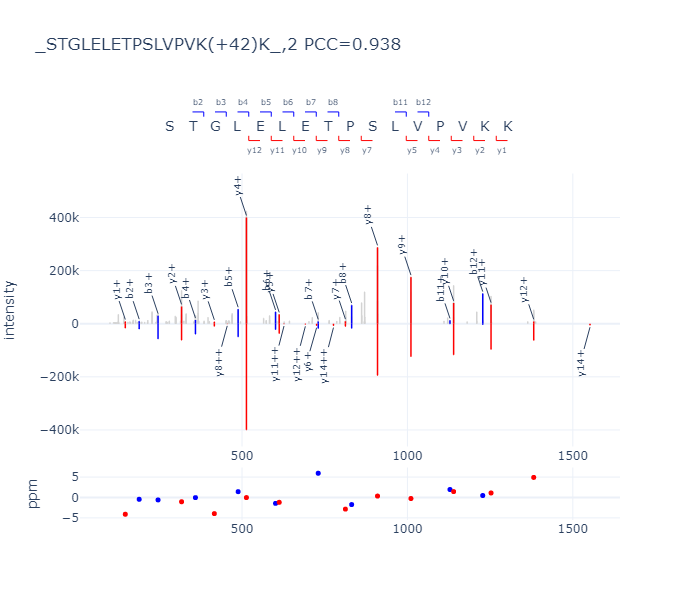

Supplement: Supplementary file 6 — Supplementary Data 3 [file 41467_2022_34904_MOESM6_ESM.zip › mirror-ms2-21ptm/Kmod_Acetyl/_STGLELETPSLVPVK(+42)K_charge=2_nce=30_pretrain_pcc=0.94.png]

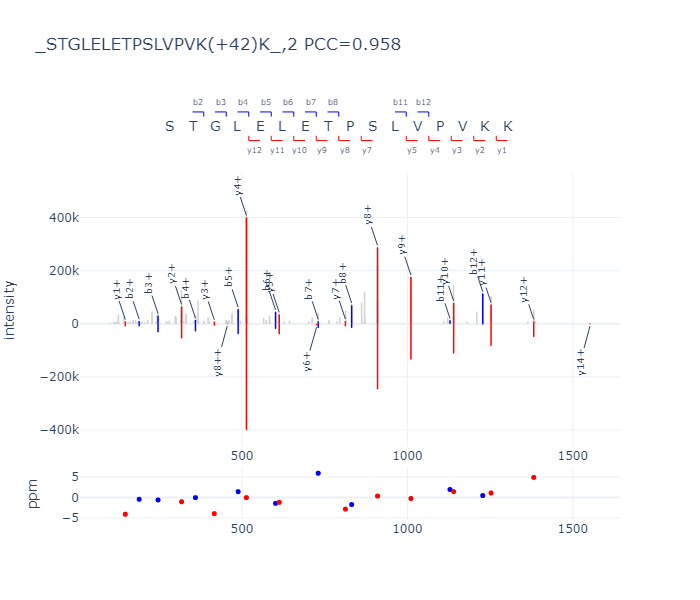

Supplement: Supplementary file 6 — Supplementary Data 3 [file 41467_2022_34904_MOESM6_ESM.zip › mirror-ms2-21ptm/Kmod_Acetyl/_STGLELETPSLVPVK(+42)K_charge=2_nce=30_transfer_pcc=0.96.png]

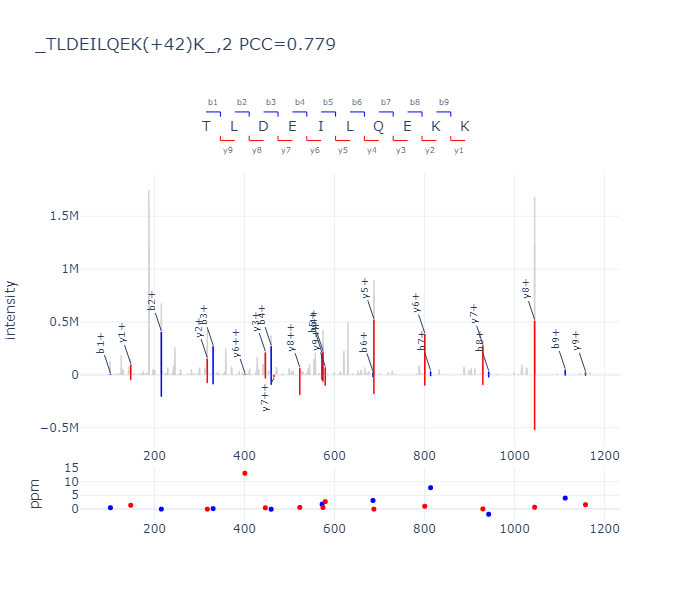

Supplement: Supplementary file 6 — Supplementary Data 3 [file 41467_2022_34904_MOESM6_ESM.zip › mirror-ms2-21ptm/Kmod_Acetyl/_TLDEILQEK(+42)K_charge=2_nce=25_pretrain_pcc=0.78.png]

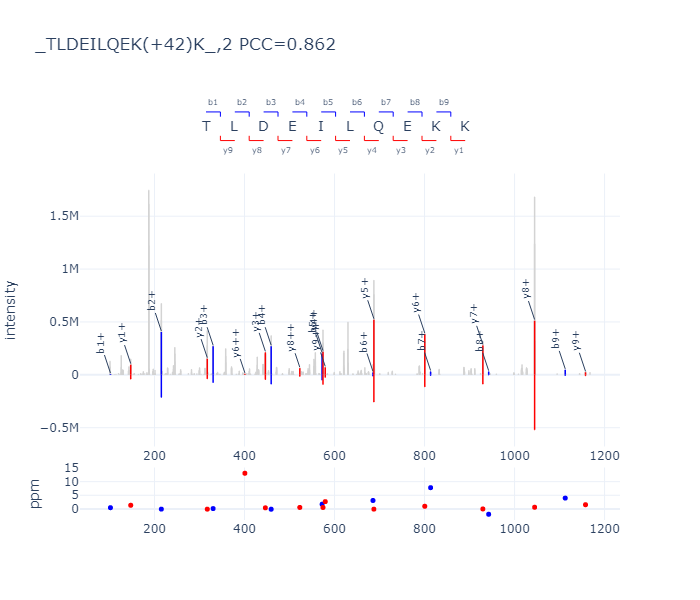

Supplement: Supplementary file 6 — Supplementary Data 3 [file 41467_2022_34904_MOESM6_ESM.zip › mirror-ms2-21ptm/Kmod_Acetyl/_TLDEILQEK(+42)K_charge=2_nce=25_transfer_pcc=0.86.png]

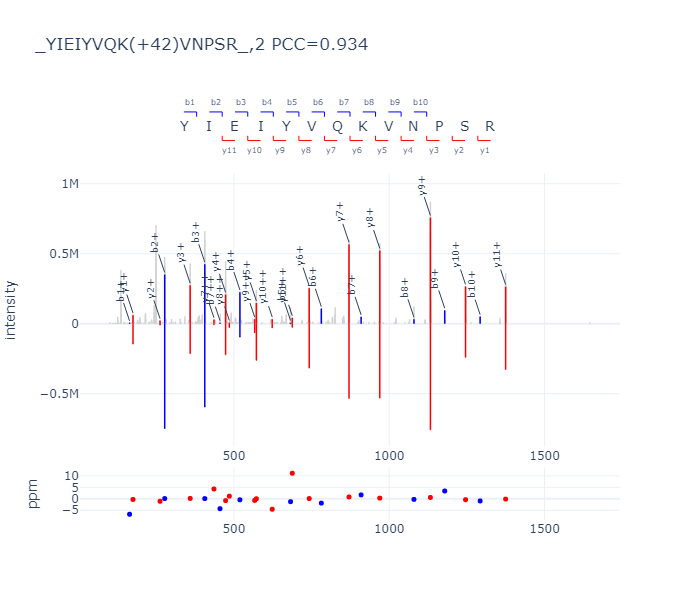

Supplement: Supplementary file 6 — Supplementary Data 3 [file 41467_2022_34904_MOESM6_ESM.zip › mirror-ms2-21ptm/Kmod_Acetyl/_YIEIYVQK(+42)VNPSR_charge=2_nce=35_pretrain_pcc=0.93.png]

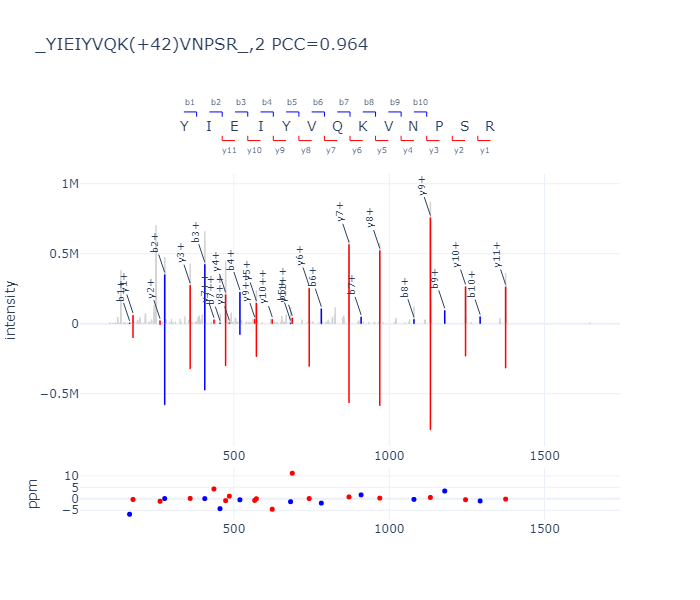

Supplement: Supplementary file 6 — Supplementary Data 3 [file 41467_2022_34904_MOESM6_ESM.zip › mirror-ms2-21ptm/Kmod_Acetyl/_YIEIYVQK(+42)VNPSR_charge=2_nce=35_transfer_pcc=0.96.png]

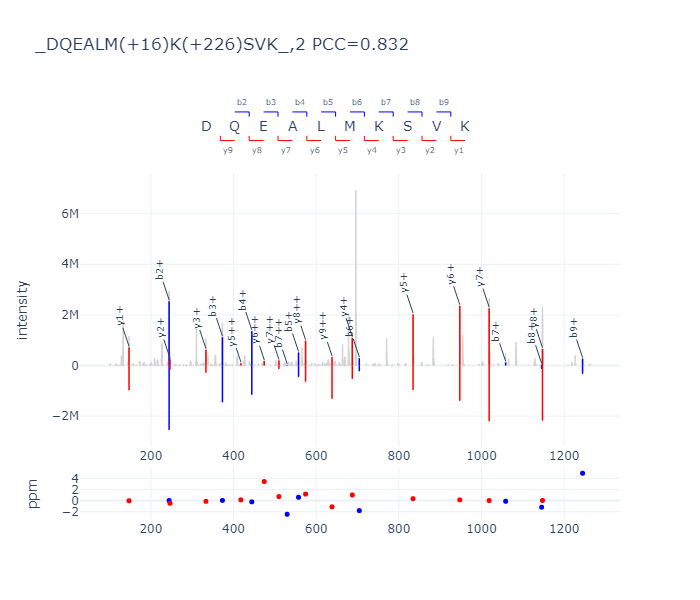

Supplement: Supplementary file 6 — Supplementary Data 3 [file 41467_2022_34904_MOESM6_ESM.zip › mirror-ms2-21ptm/Kmod_Biotin/_DQEALM(+16)K(+226)SVK_charge=2_nce=25_pretrain_pcc=0.83.png]

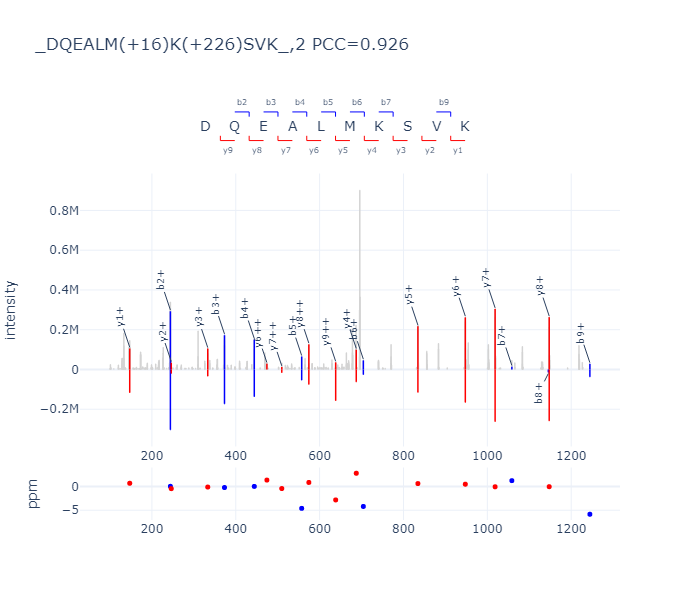

Supplement: Supplementary file 6 — Supplementary Data 3 [file 41467_2022_34904_MOESM6_ESM.zip › mirror-ms2-21ptm/Kmod_Biotin/_DQEALM(+16)K(+226)SVK_charge=2_nce=25_pretrain_pcc=0.93.png]

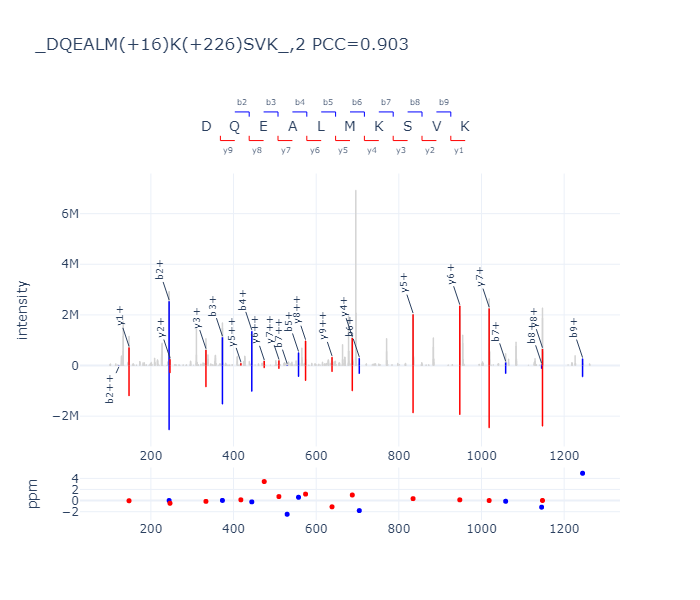

Supplement: Supplementary file 6 — Supplementary Data 3 [file 41467_2022_34904_MOESM6_ESM.zip › mirror-ms2-21ptm/Kmod_Biotin/_DQEALM(+16)K(+226)SVK_charge=2_nce=25_transfer_pcc=0.90.png]

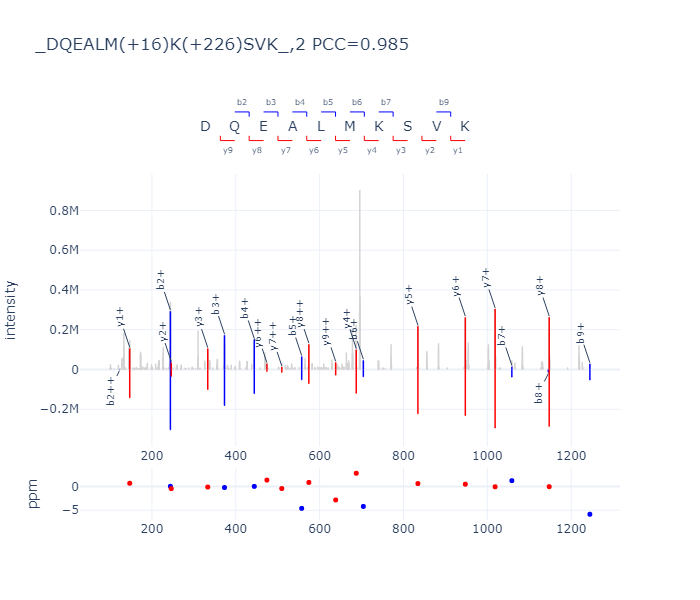

Supplement: Supplementary file 6 — Supplementary Data 3 [file 41467_2022_34904_MOESM6_ESM.zip › mirror-ms2-21ptm/Kmod_Biotin/_DQEALM(+16)K(+226)SVK_charge=2_nce=25_transfer_pcc=0.98.png]

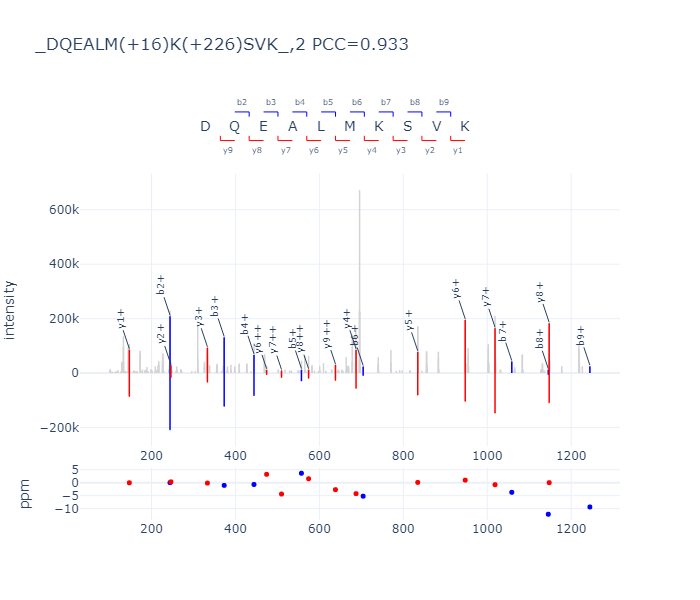

Supplement: Supplementary file 6 — Supplementary Data 3 [file 41467_2022_34904_MOESM6_ESM.zip › mirror-ms2-21ptm/Kmod_Biotin/_DQEALM(+16)K(+226)SVK_charge=2_nce=30_pretrain_pcc=0.93.png]

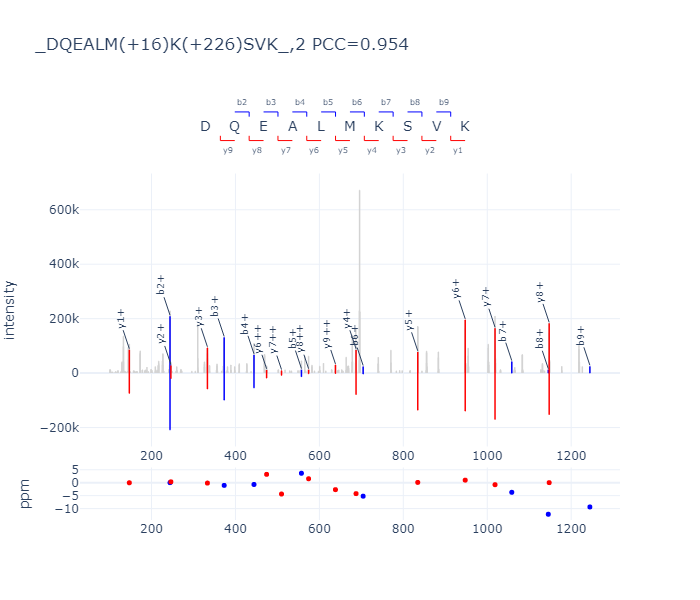

Supplement: Supplementary file 6 — Supplementary Data 3 [file 41467_2022_34904_MOESM6_ESM.zip › mirror-ms2-21ptm/Kmod_Biotin/_DQEALM(+16)K(+226)SVK_charge=2_nce=30_transfer_pcc=0.95.png]

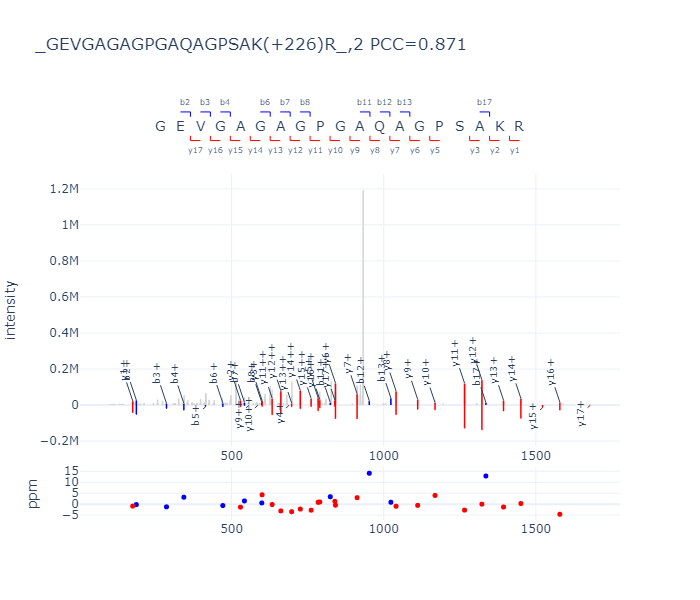

Supplement: Supplementary file 6 — Supplementary Data 3 [file 41467_2022_34904_MOESM6_ESM.zip › mirror-ms2-21ptm/Kmod_Biotin/_GEVGAGAGPGAQAGPSAK(+226)R_charge=2_nce=35_pretrain_pcc=0.87.png]

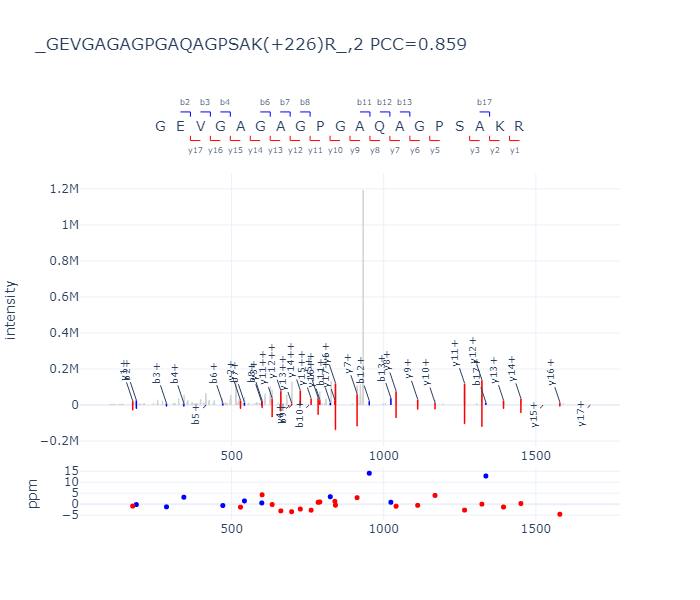

Supplement: Supplementary file 6 — Supplementary Data 3 [file 41467_2022_34904_MOESM6_ESM.zip › mirror-ms2-21ptm/Kmod_Biotin/_GEVGAGAGPGAQAGPSAK(+226)R_charge=2_nce=35_transfer_pcc=0.86.png]

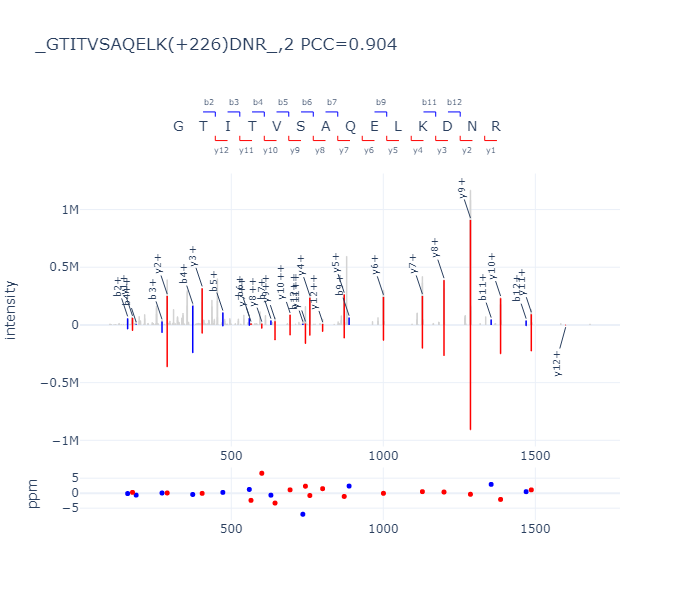

Supplement: Supplementary file 6 — Supplementary Data 3 [file 41467_2022_34904_MOESM6_ESM.zip › mirror-ms2-21ptm/Kmod_Biotin/_GTITVSAQELK(+226)DNR_charge=2_nce=30_pretrain_pcc=0.90.png]

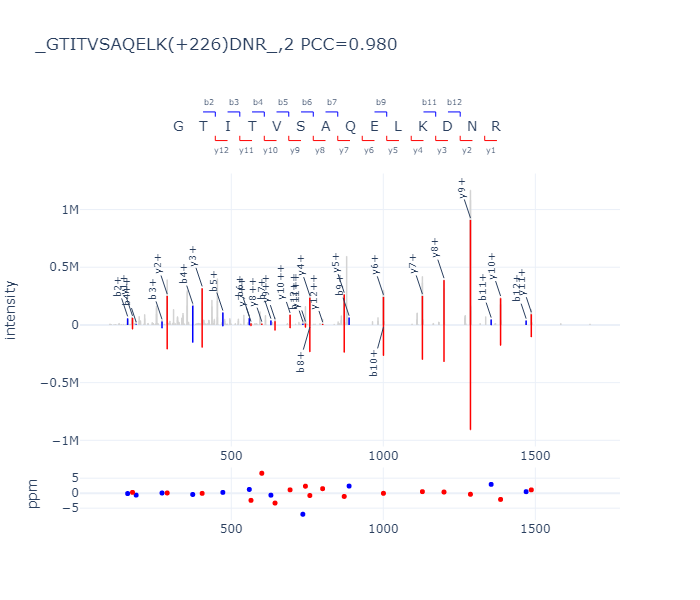

Supplement: Supplementary file 6 — Supplementary Data 3 [file 41467_2022_34904_MOESM6_ESM.zip › mirror-ms2-21ptm/Kmod_Biotin/_GTITVSAQELK(+226)DNR_charge=2_nce=30_transfer_pcc=0.98.png]

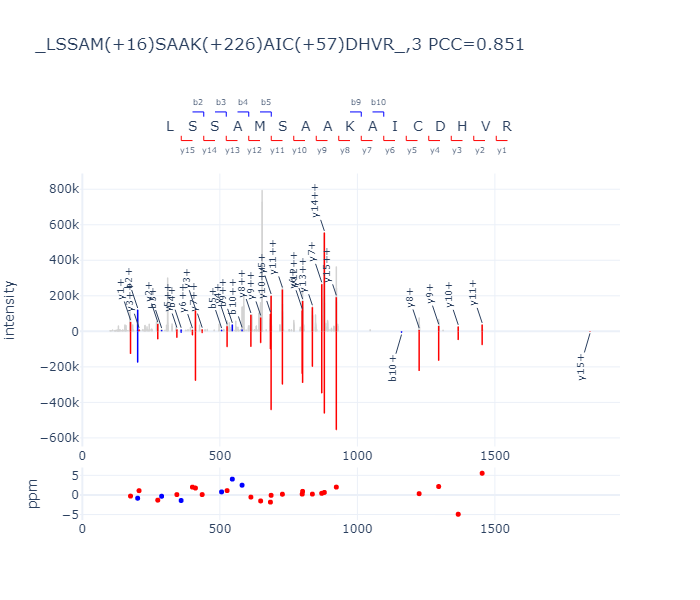

Supplement: Supplementary file 6 — Supplementary Data 3 [file 41467_2022_34904_MOESM6_ESM.zip › mirror-ms2-21ptm/Kmod_Biotin/_LSSAM(+16)SAAK(+226)AIC(+57)DHVR_charge=3_nce=30_pretrain_pcc=0.85.png]

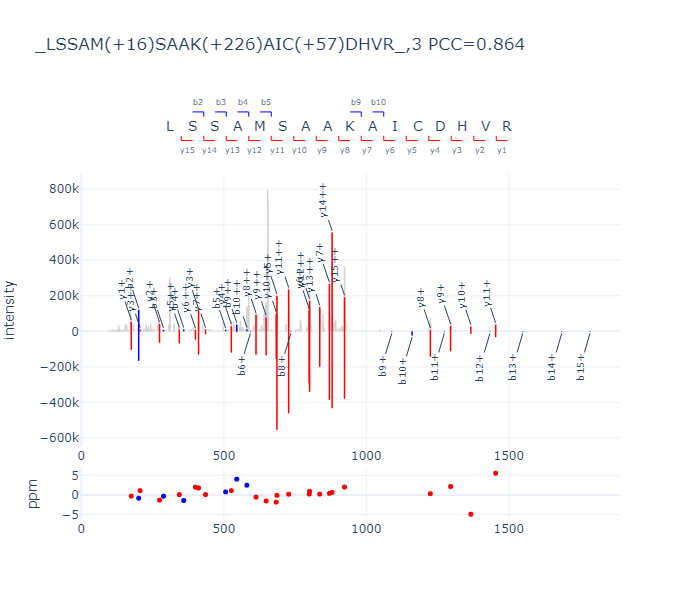

Supplement: Supplementary file 6 — Supplementary Data 3 [file 41467_2022_34904_MOESM6_ESM.zip › mirror-ms2-21ptm/Kmod_Biotin/_LSSAM(+16)SAAK(+226)AIC(+57)DHVR_charge=3_nce=30_transfer_pcc=0.86.png]

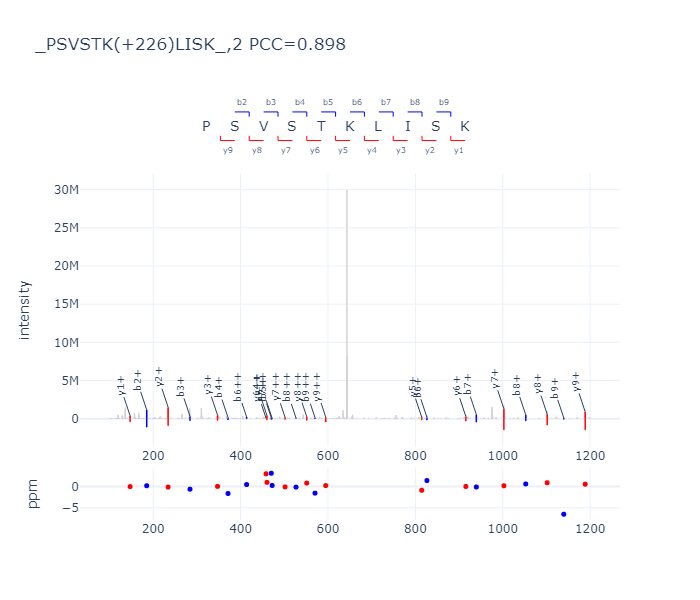

Supplement: Supplementary file 6 — Supplementary Data 3 [file 41467_2022_34904_MOESM6_ESM.zip › mirror-ms2-21ptm/Kmod_Biotin/_PSVSTK(+226)LISK_charge=2_nce=30_pretrain_pcc=0.90.png]

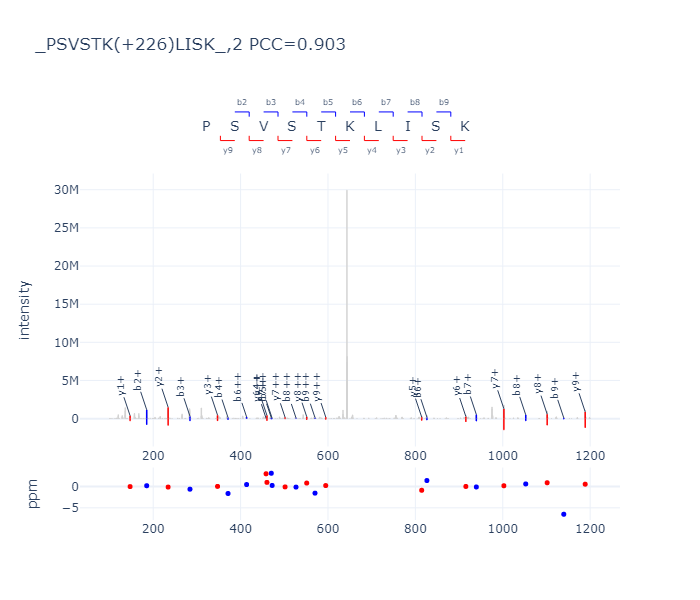

Supplement: Supplementary file 6 — Supplementary Data 3 [file 41467_2022_34904_MOESM6_ESM.zip › mirror-ms2-21ptm/Kmod_Biotin/_PSVSTK(+226)LISK_charge=2_nce=30_transfer_pcc=0.90.png]

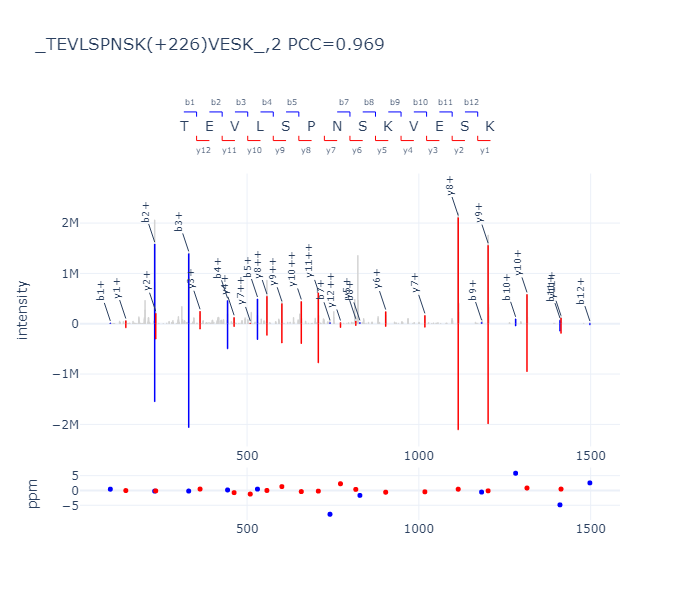

Supplement: Supplementary file 6 — Supplementary Data 3 [file 41467_2022_34904_MOESM6_ESM.zip › mirror-ms2-21ptm/Kmod_Biotin/_TEVLSPNSK(+226)VESK_charge=2_nce=25_pretrain_pcc=0.97.png]

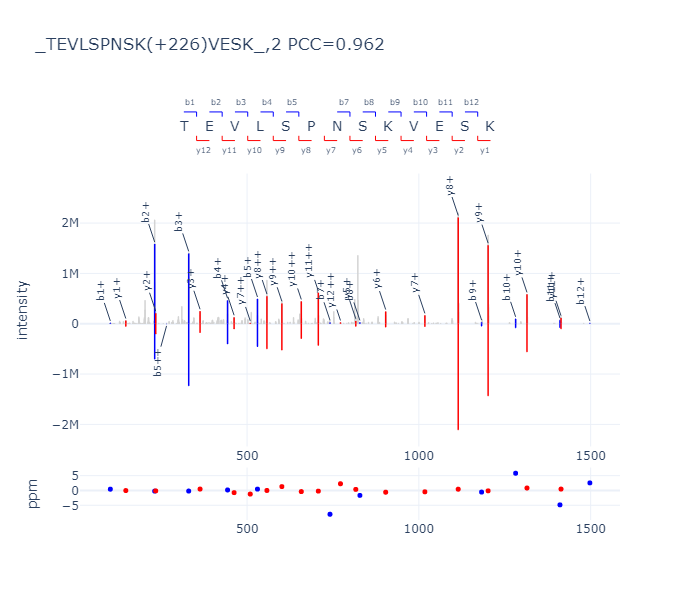

Supplement: Supplementary file 6 — Supplementary Data 3 [file 41467_2022_34904_MOESM6_ESM.zip › mirror-ms2-21ptm/Kmod_Biotin/_TEVLSPNSK(+226)VESK_charge=2_nce=25_transfer_pcc=0.96.png]

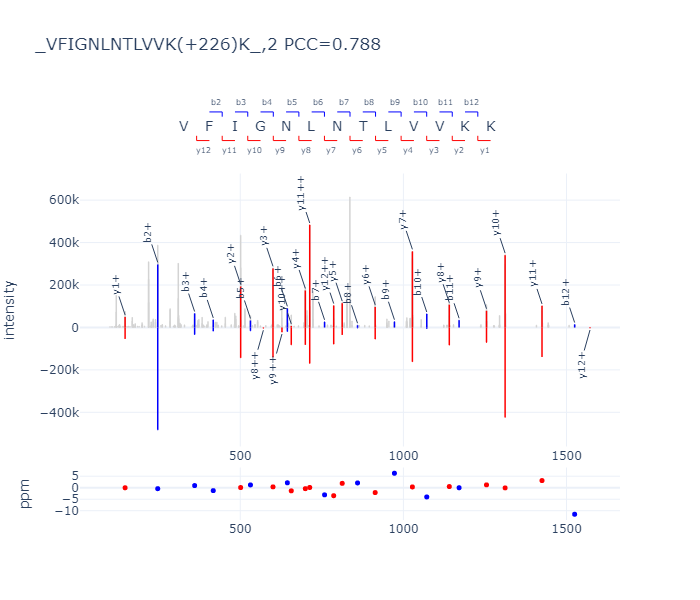

Supplement: Supplementary file 6 — Supplementary Data 3 [file 41467_2022_34904_MOESM6_ESM.zip › mirror-ms2-21ptm/Kmod_Biotin/_VFIGNLNTLVVK(+226)K_charge=2_nce=30_pretrain_pcc=0.79.png]

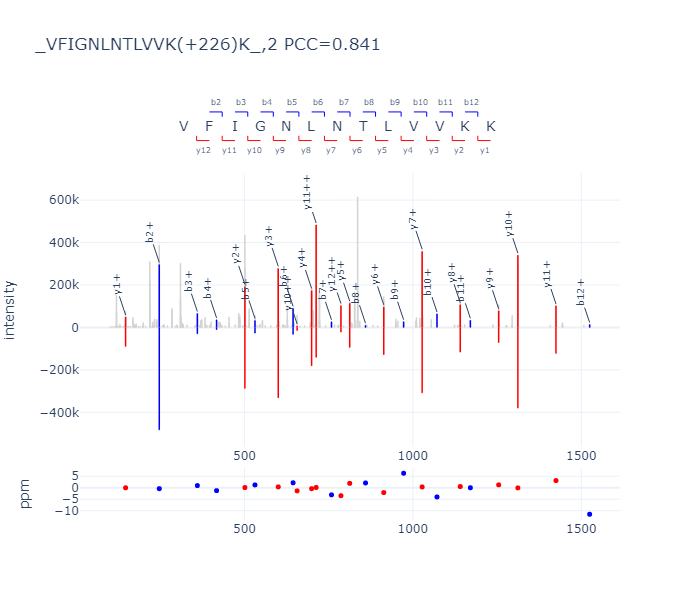

Supplement: Supplementary file 6 — Supplementary Data 3 [file 41467_2022_34904_MOESM6_ESM.zip › mirror-ms2-21ptm/Kmod_Biotin/_VFIGNLNTLVVK(+226)K_charge=2_nce=30_transfer_pcc=0.84.png]

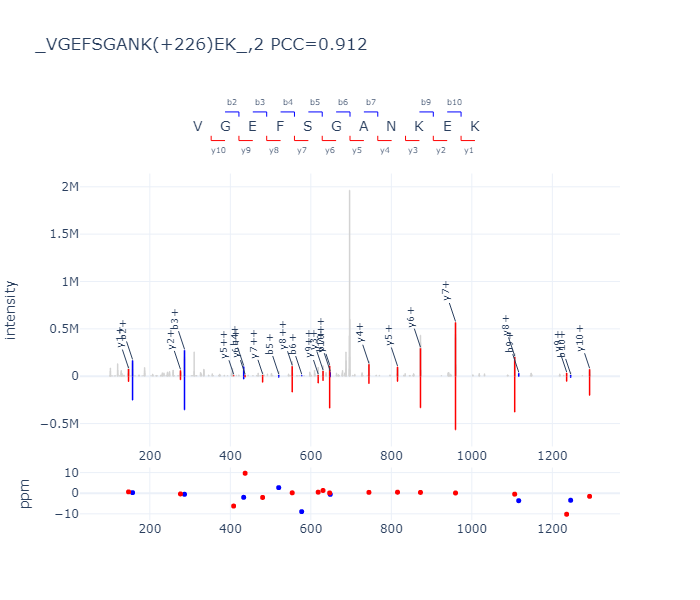

Supplement: Supplementary file 6 — Supplementary Data 3 [file 41467_2022_34904_MOESM6_ESM.zip › mirror-ms2-21ptm/Kmod_Biotin/_VGEFSGANK(+226)EK_charge=2_nce=30_pretrain_pcc=0.91.png]

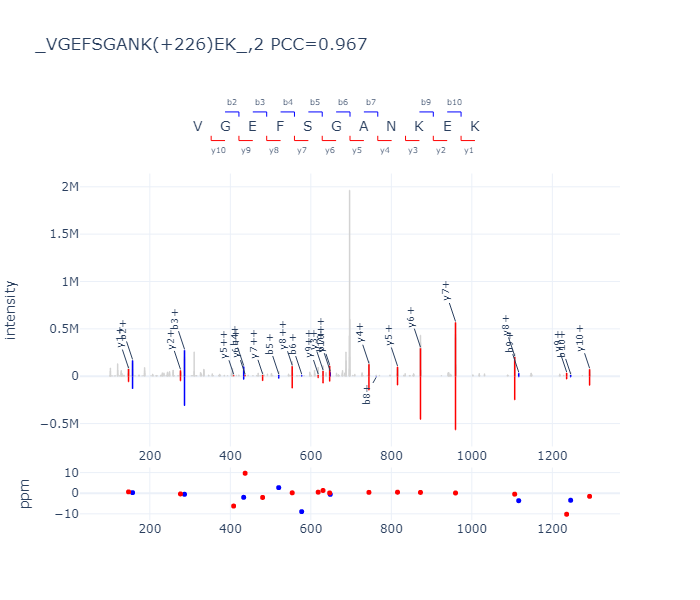

Supplement: Supplementary file 6 — Supplementary Data 3 [file 41467_2022_34904_MOESM6_ESM.zip › mirror-ms2-21ptm/Kmod_Biotin/_VGEFSGANK(+226)EK_charge=2_nce=30_transfer_pcc=0.97.png]

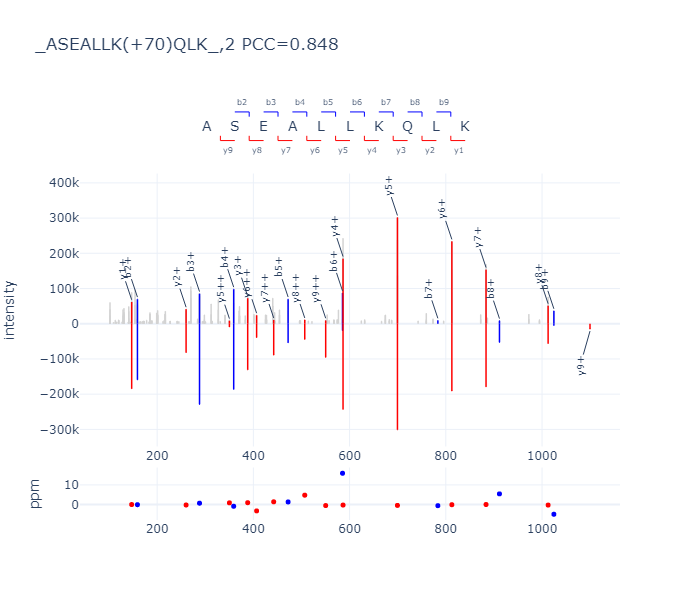

Supplement: Supplementary file 6 — Supplementary Data 3 [file 41467_2022_34904_MOESM6_ESM.zip › mirror-ms2-21ptm/Kmod_Butyryl/_ASEALLK(+70)QLK_charge=2_nce=35_pretrain_pcc=0.85.png]

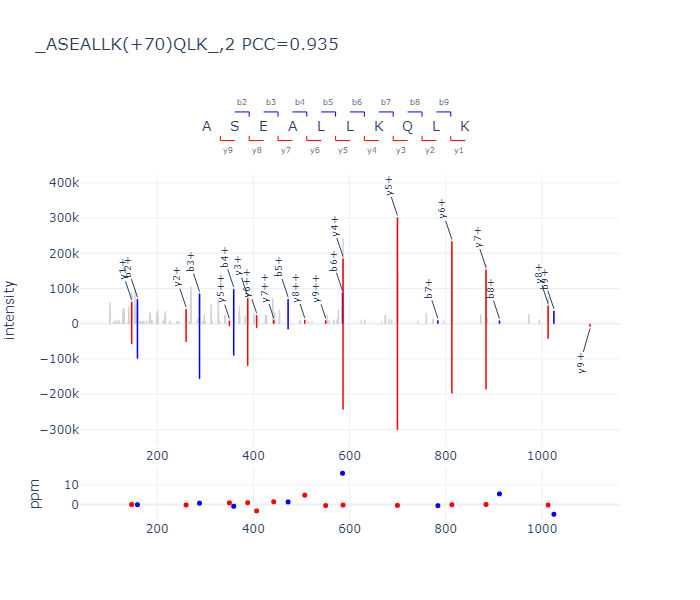

Supplement: Supplementary file 6 — Supplementary Data 3 [file 41467_2022_34904_MOESM6_ESM.zip › mirror-ms2-21ptm/Kmod_Butyryl/_ASEALLK(+70)QLK_charge=2_nce=35_transfer_pcc=0.93.png]

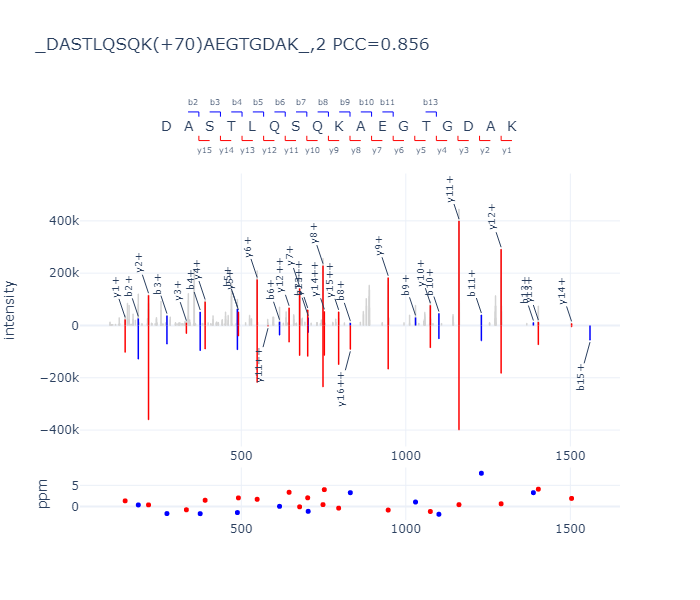

Supplement: Supplementary file 6 — Supplementary Data 3 [file 41467_2022_34904_MOESM6_ESM.zip › mirror-ms2-21ptm/Kmod_Butyryl/_DASTLQSQK(+70)AEGTGDAK_charge=2_nce=30_pretrain_pcc=0.86.png]

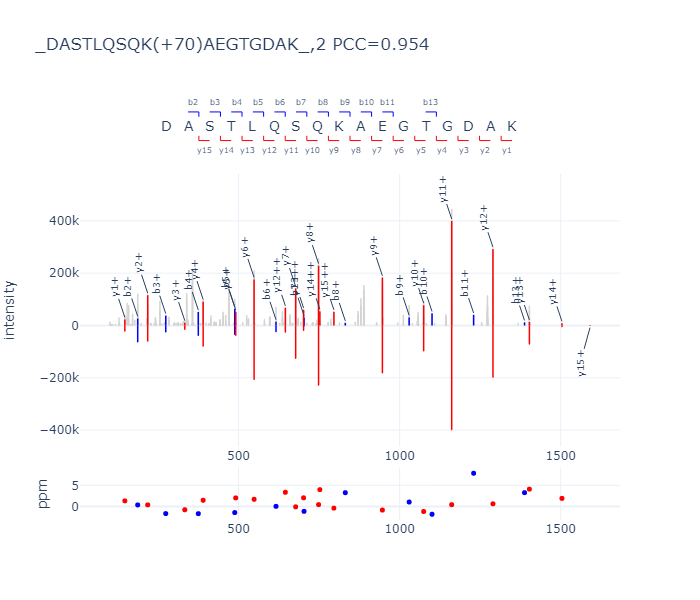

Supplement: Supplementary file 6 — Supplementary Data 3 [file 41467_2022_34904_MOESM6_ESM.zip › mirror-ms2-21ptm/Kmod_Butyryl/_DASTLQSQK(+70)AEGTGDAK_charge=2_nce=30_transfer_pcc=0.95.png]

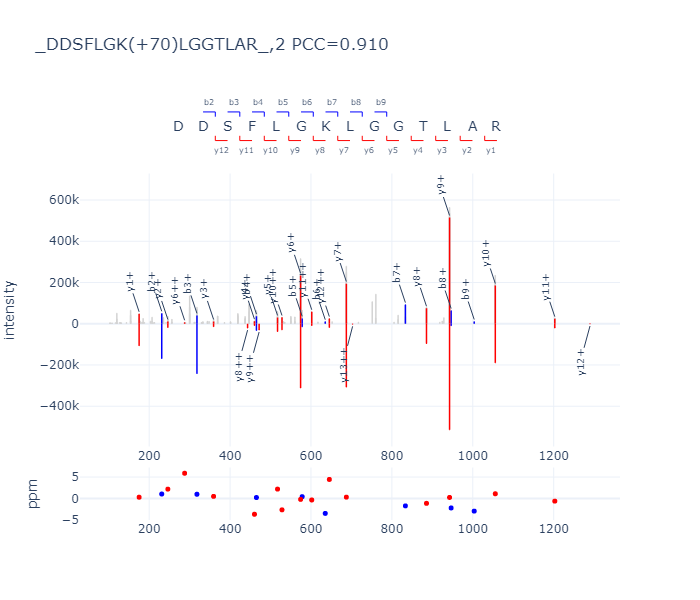

Supplement: Supplementary file 6 — Supplementary Data 3 [file 41467_2022_34904_MOESM6_ESM.zip › mirror-ms2-21ptm/Kmod_Butyryl/_DDSFLGK(+70)LGGTLAR_charge=2_nce=35_pretrain_pcc=0.91.png]

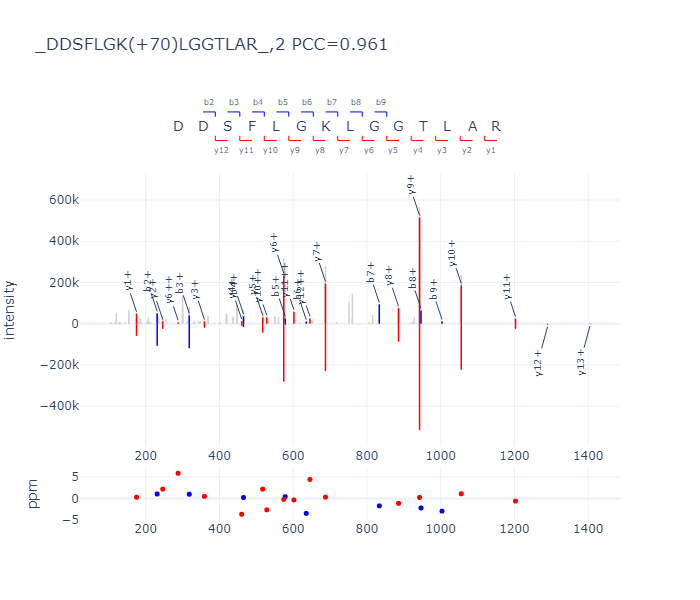

Supplement: Supplementary file 6 — Supplementary Data 3 [file 41467_2022_34904_MOESM6_ESM.zip › mirror-ms2-21ptm/Kmod_Butyryl/_DDSFLGK(+70)LGGTLAR_charge=2_nce=35_transfer_pcc=0.96.png]

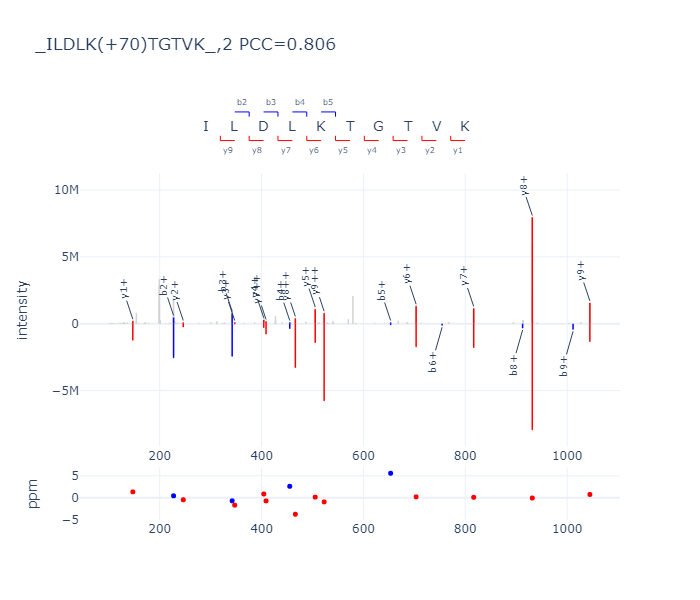

Supplement: Supplementary file 6 — Supplementary Data 3 [file 41467_2022_34904_MOESM6_ESM.zip › mirror-ms2-21ptm/Kmod_Butyryl/_ILDLK(+70)TGTVK_charge=2_nce=25_pretrain_pcc=0.81.png]

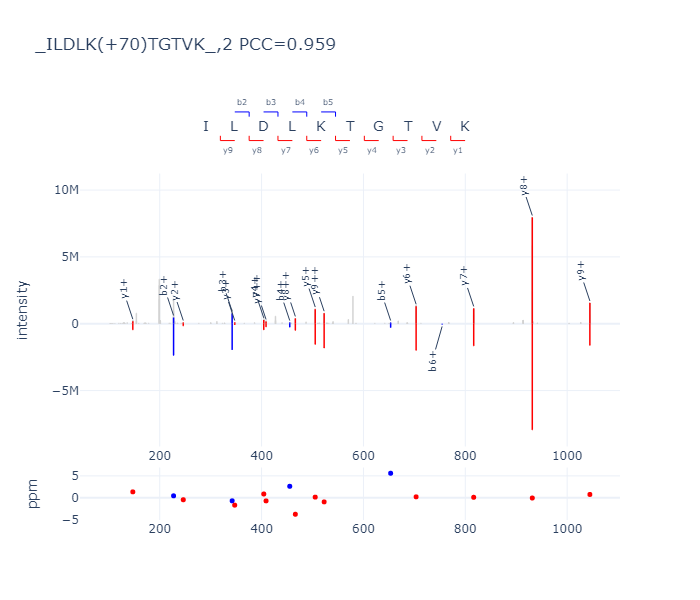

Supplement: Supplementary file 6 — Supplementary Data 3 [file 41467_2022_34904_MOESM6_ESM.zip › mirror-ms2-21ptm/Kmod_Butyryl/_ILDLK(+70)TGTVK_charge=2_nce=25_transfer_pcc=0.96.png]

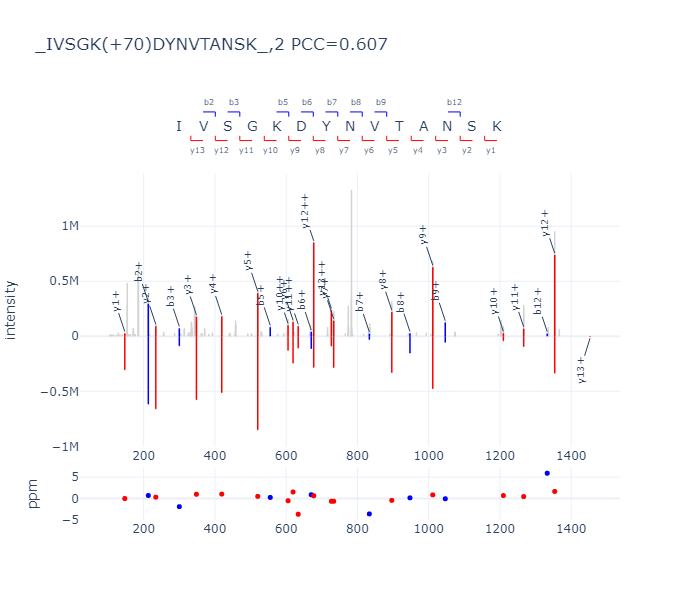

Supplement: Supplementary file 6 — Supplementary Data 3 [file 41467_2022_34904_MOESM6_ESM.zip › mirror-ms2-21ptm/Kmod_Butyryl/_IVSGK(+70)DYNVTANSK_charge=2_nce=35_pretrain_pcc=0.61.png]

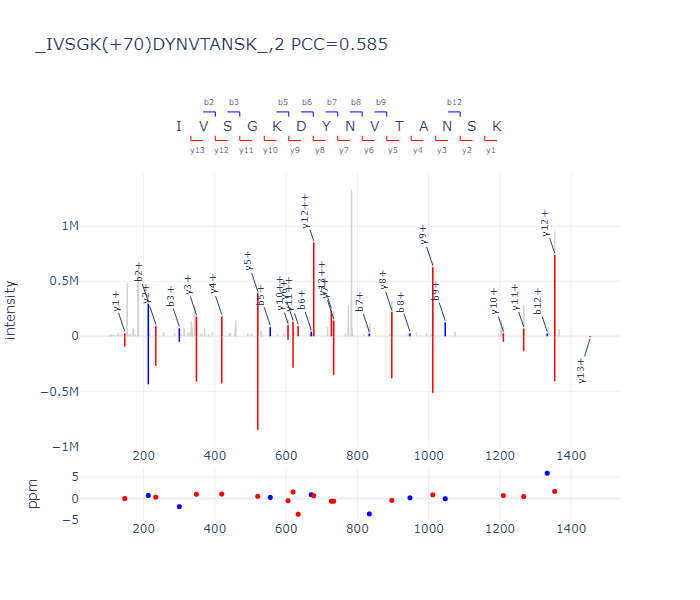

Supplement: Supplementary file 6 — Supplementary Data 3 [file 41467_2022_34904_MOESM6_ESM.zip › mirror-ms2-21ptm/Kmod_Butyryl/_IVSGK(+70)DYNVTANSK_charge=2_nce=35_transfer_pcc=0.59.png]

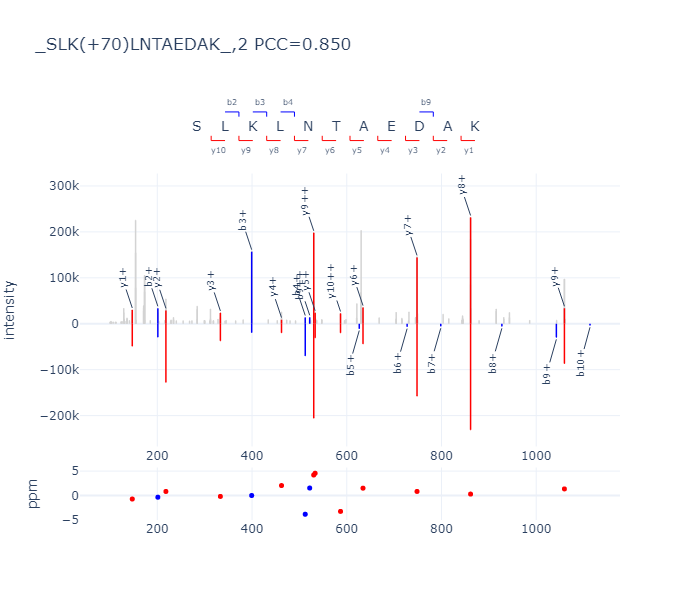

Supplement: Supplementary file 6 — Supplementary Data 3 [file 41467_2022_34904_MOESM6_ESM.zip › mirror-ms2-21ptm/Kmod_Butyryl/_SLK(+70)LNTAEDAK_charge=2_nce=30_pretrain_pcc=0.85.png]

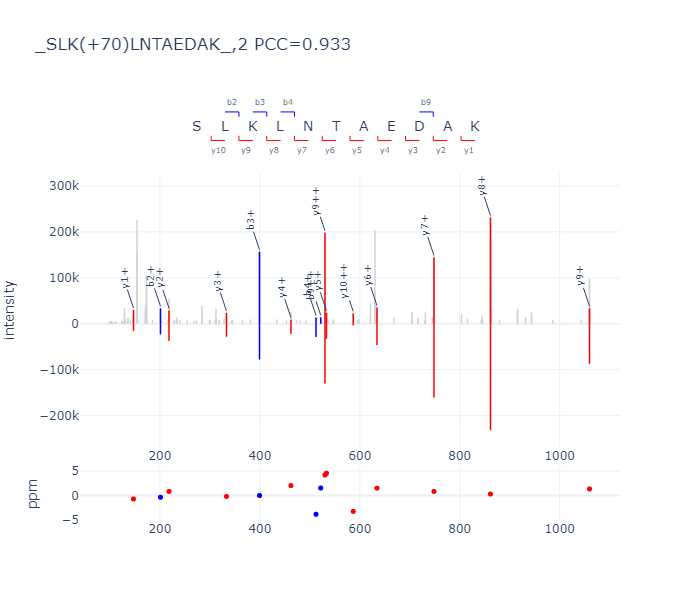

Supplement: Supplementary file 6 — Supplementary Data 3 [file 41467_2022_34904_MOESM6_ESM.zip › mirror-ms2-21ptm/Kmod_Butyryl/_SLK(+70)LNTAEDAK_charge=2_nce=30_transfer_pcc=0.93.png]

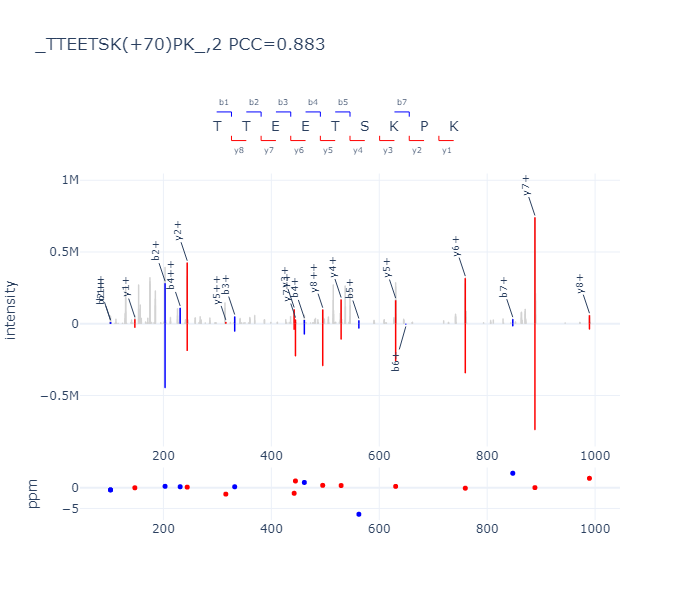

Supplement: Supplementary file 6 — Supplementary Data 3 [file 41467_2022_34904_MOESM6_ESM.zip › mirror-ms2-21ptm/Kmod_Butyryl/_TTEETSK(+70)PK_charge=2_nce=25_pretrain_pcc=0.88.png]

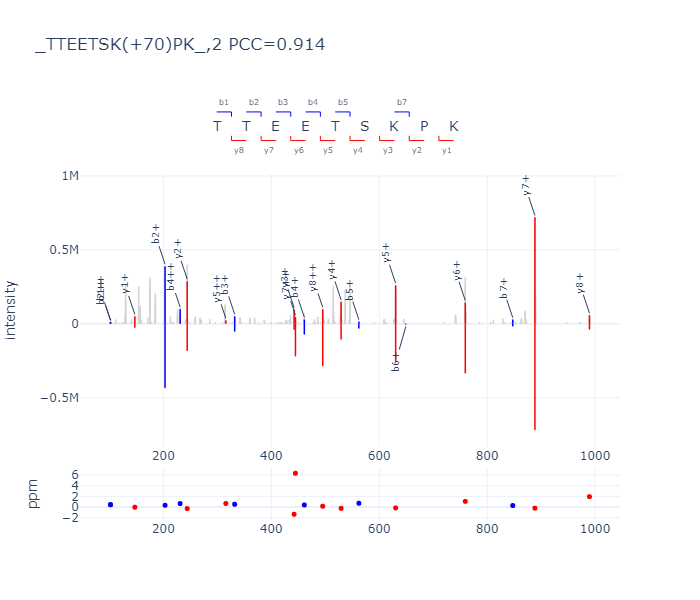

Supplement: Supplementary file 6 — Supplementary Data 3 [file 41467_2022_34904_MOESM6_ESM.zip › mirror-ms2-21ptm/Kmod_Butyryl/_TTEETSK(+70)PK_charge=2_nce=25_pretrain_pcc=0.91.png]

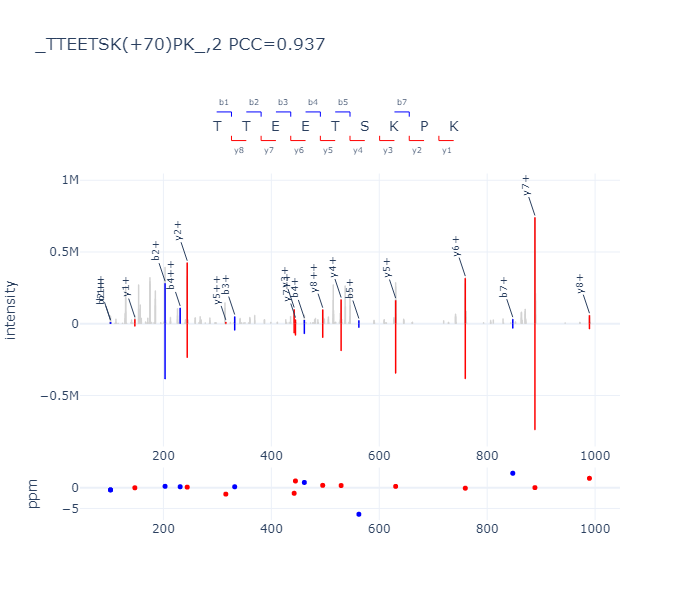

Supplement: Supplementary file 6 — Supplementary Data 3 [file 41467_2022_34904_MOESM6_ESM.zip › mirror-ms2-21ptm/Kmod_Butyryl/_TTEETSK(+70)PK_charge=2_nce=25_transfer_pcc=0.94.png]

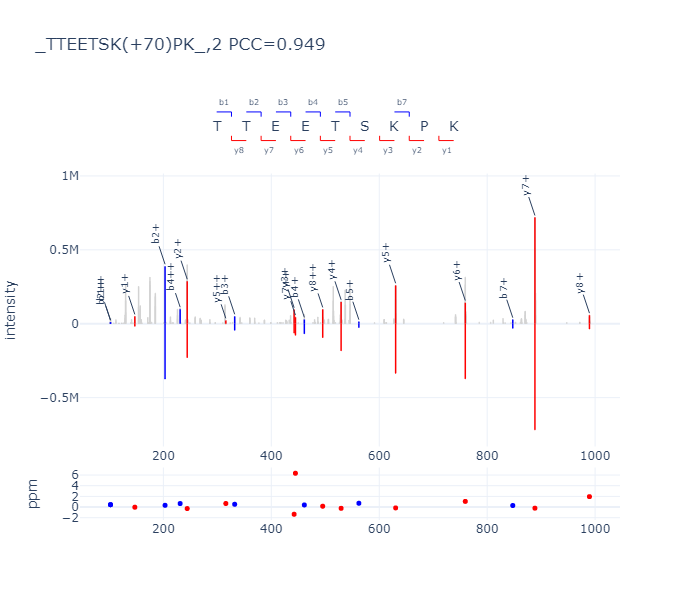

Supplement: Supplementary file 6 — Supplementary Data 3 [file 41467_2022_34904_MOESM6_ESM.zip › mirror-ms2-21ptm/Kmod_Butyryl/_TTEETSK(+70)PK_charge=2_nce=25_transfer_pcc=0.95.png]

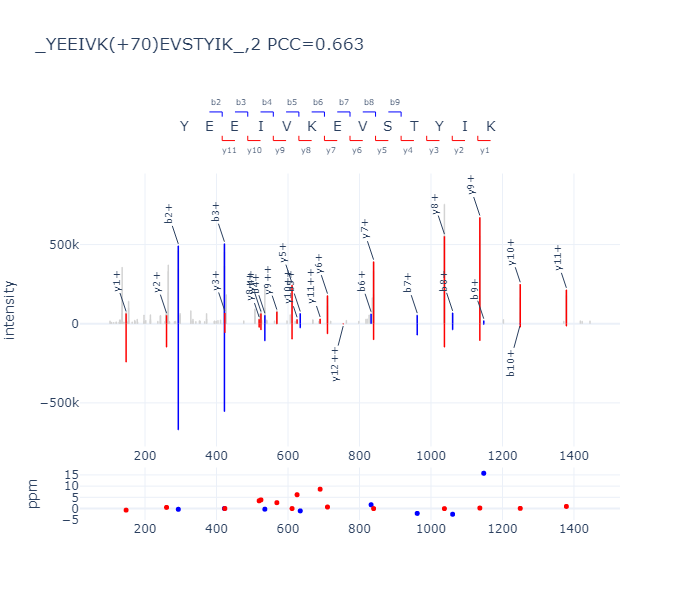

Supplement: Supplementary file 6 — Supplementary Data 3 [file 41467_2022_34904_MOESM6_ESM.zip › mirror-ms2-21ptm/Kmod_Butyryl/_YEEIVK(+70)EVSTYIK_charge=2_nce=35_pretrain_pcc=0.66.png]

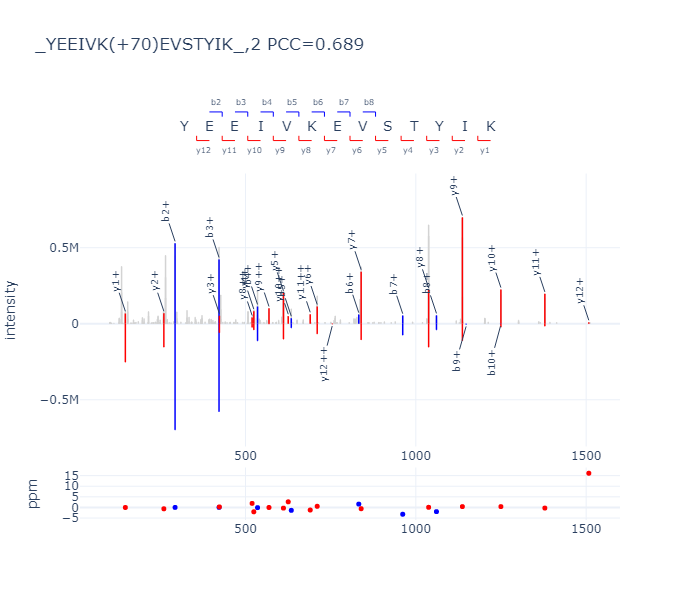

Supplement: Supplementary file 6 — Supplementary Data 3 [file 41467_2022_34904_MOESM6_ESM.zip › mirror-ms2-21ptm/Kmod_Butyryl/_YEEIVK(+70)EVSTYIK_charge=2_nce=35_pretrain_pcc=0.69.png]

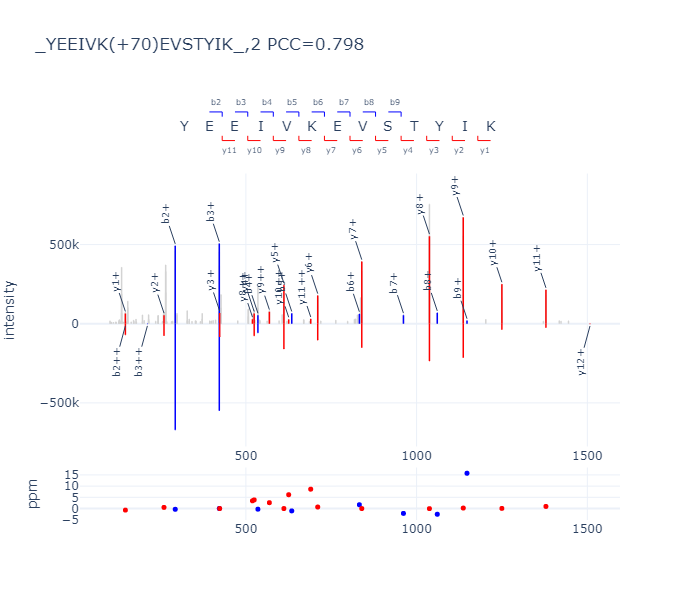

Supplement: Supplementary file 6 — Supplementary Data 3 [file 41467_2022_34904_MOESM6_ESM.zip › mirror-ms2-21ptm/Kmod_Butyryl/_YEEIVK(+70)EVSTYIK_charge=2_nce=35_transfer_pcc=0.80.png]

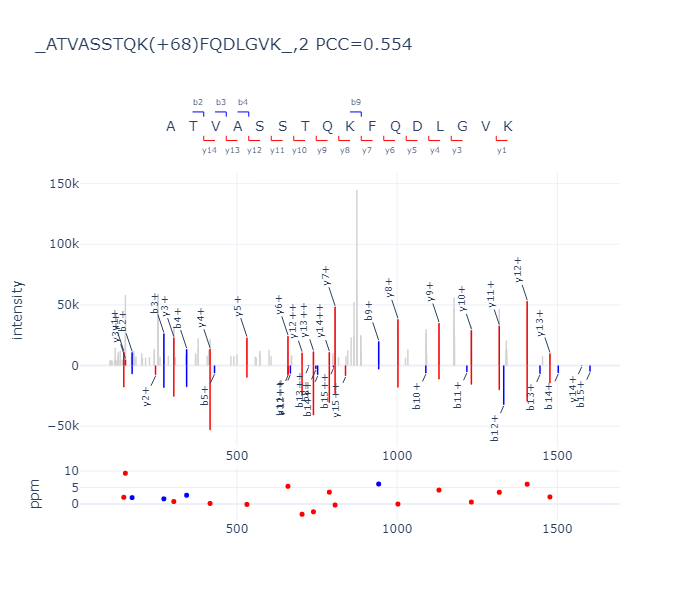

Supplement: Supplementary file 6 — Supplementary Data 3 [file 41467_2022_34904_MOESM6_ESM.zip › mirror-ms2-21ptm/Kmod_Crotonyl/_ATVASSTQK(+68)FQDLGVK_charge=2_nce=25_pretrain_pcc=0.55.png]

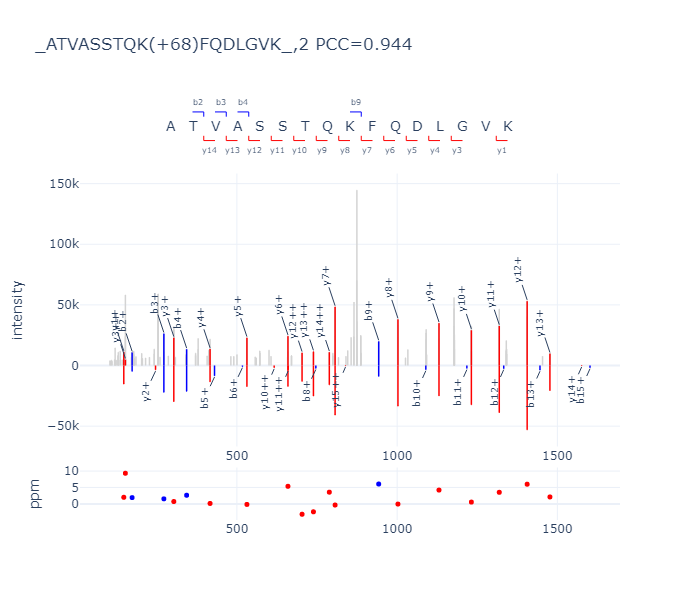

Supplement: Supplementary file 6 — Supplementary Data 3 [file 41467_2022_34904_MOESM6_ESM.zip › mirror-ms2-21ptm/Kmod_Crotonyl/_ATVASSTQK(+68)FQDLGVK_charge=2_nce=25_transfer_pcc=0.94.png]

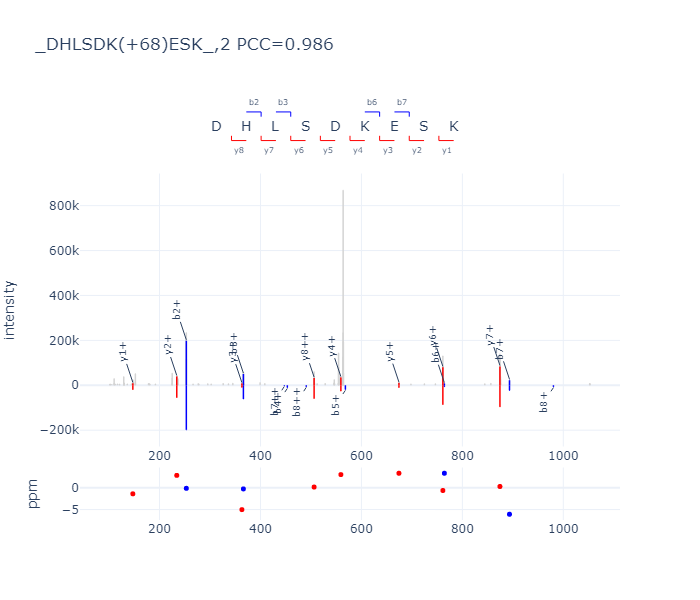

Supplement: Supplementary file 6 — Supplementary Data 3 [file 41467_2022_34904_MOESM6_ESM.zip › mirror-ms2-21ptm/Kmod_Crotonyl/_DHLSDK(+68)ESK_charge=2_nce=30_pretrain_pcc=0.99.png]

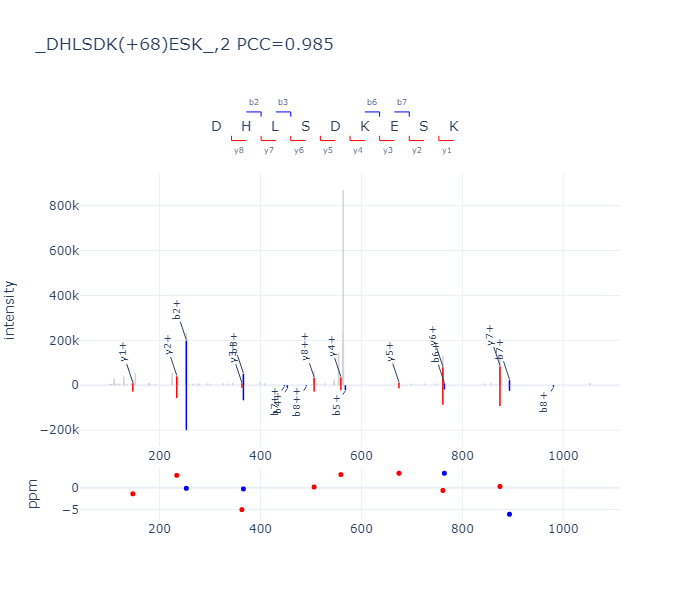

Supplement: Supplementary file 6 — Supplementary Data 3 [file 41467_2022_34904_MOESM6_ESM.zip › mirror-ms2-21ptm/Kmod_Crotonyl/_DHLSDK(+68)ESK_charge=2_nce=30_transfer_pcc=0.99.png]

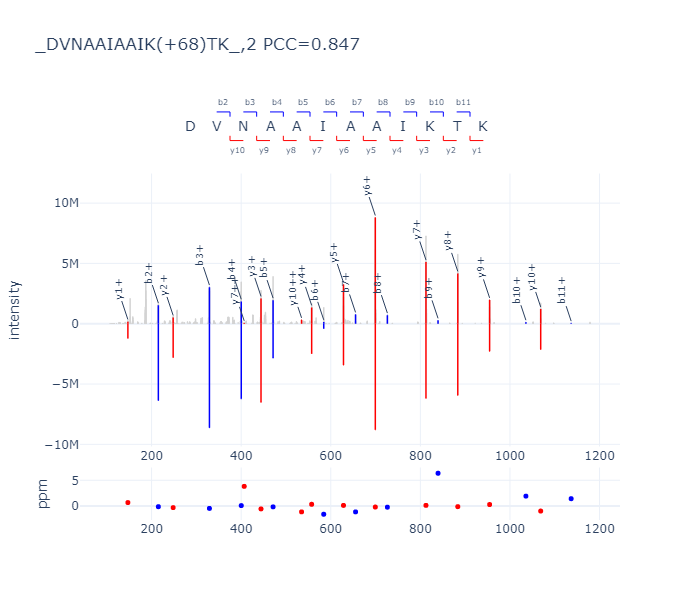

Supplement: Supplementary file 6 — Supplementary Data 3 [file 41467_2022_34904_MOESM6_ESM.zip › mirror-ms2-21ptm/Kmod_Crotonyl/_DVNAAIAAIK(+68)TK_charge=2_nce=35_pretrain_pcc=0.85.png]

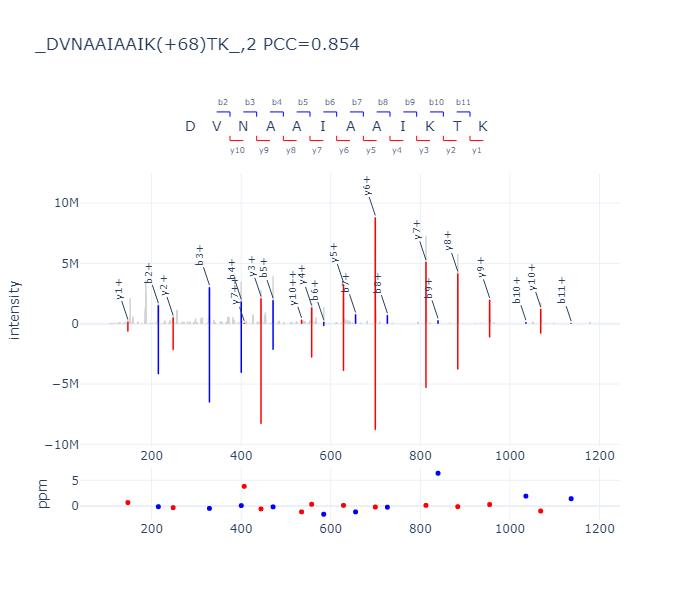

Supplement: Supplementary file 6 — Supplementary Data 3 [file 41467_2022_34904_MOESM6_ESM.zip › mirror-ms2-21ptm/Kmod_Crotonyl/_DVNAAIAAIK(+68)TK_charge=2_nce=35_transfer_pcc=0.85.png]

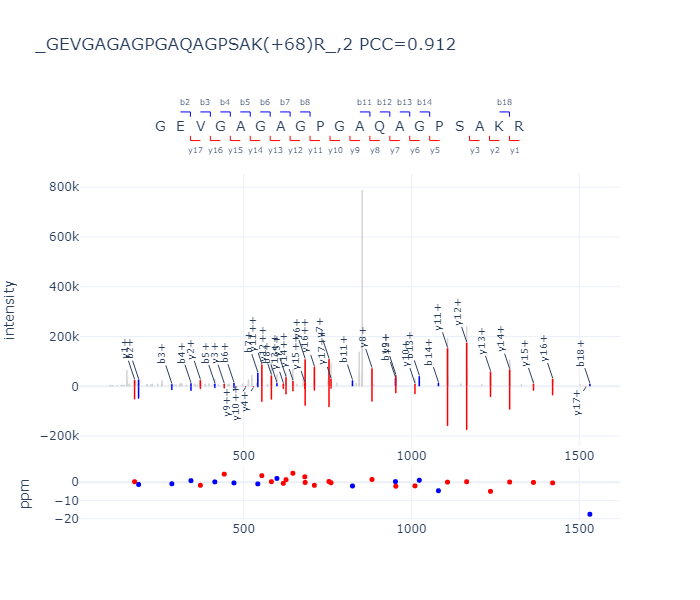

Supplement: Supplementary file 6 — Supplementary Data 3 [file 41467_2022_34904_MOESM6_ESM.zip › mirror-ms2-21ptm/Kmod_Crotonyl/_GEVGAGAGPGAQAGPSAK(+68)R_charge=2_nce=35_pretrain_pcc=0.91.png]

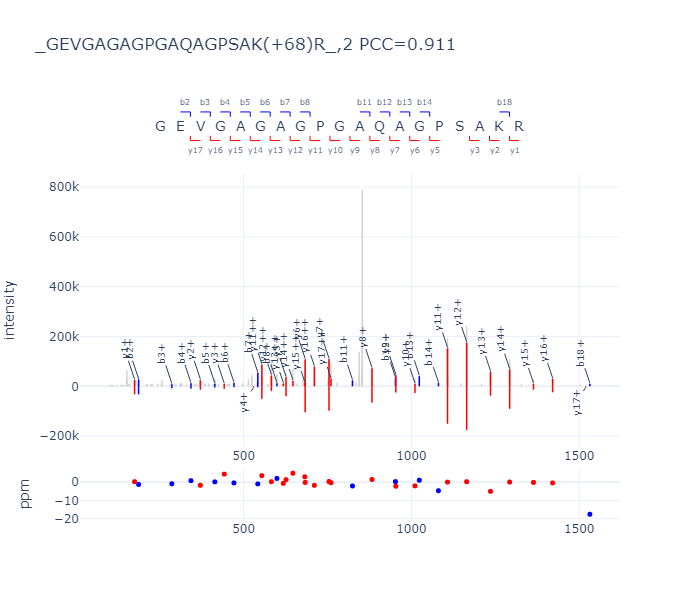

Supplement: Supplementary file 6 — Supplementary Data 3 [file 41467_2022_34904_MOESM6_ESM.zip › mirror-ms2-21ptm/Kmod_Crotonyl/_GEVGAGAGPGAQAGPSAK(+68)R_charge=2_nce=35_transfer_pcc=0.91.png]

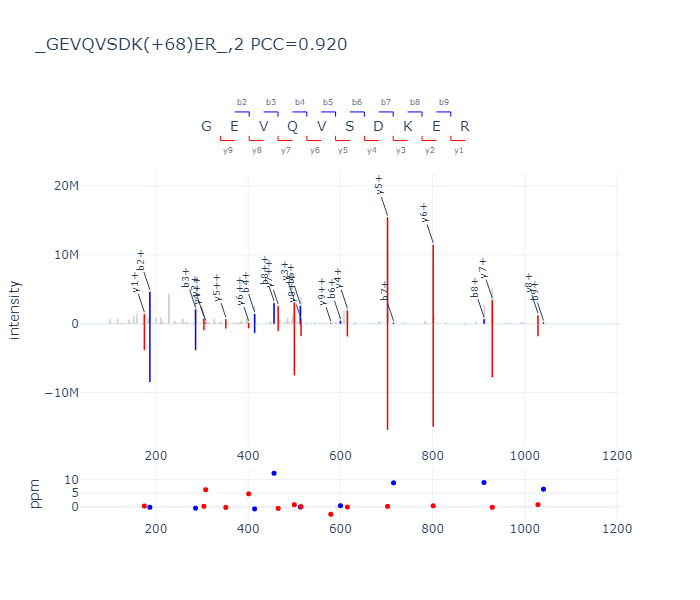

Supplement: Supplementary file 6 — Supplementary Data 3 [file 41467_2022_34904_MOESM6_ESM.zip › mirror-ms2-21ptm/Kmod_Crotonyl/_GEVQVSDK(+68)ER_charge=2_nce=35_pretrain_pcc=0.92.png]

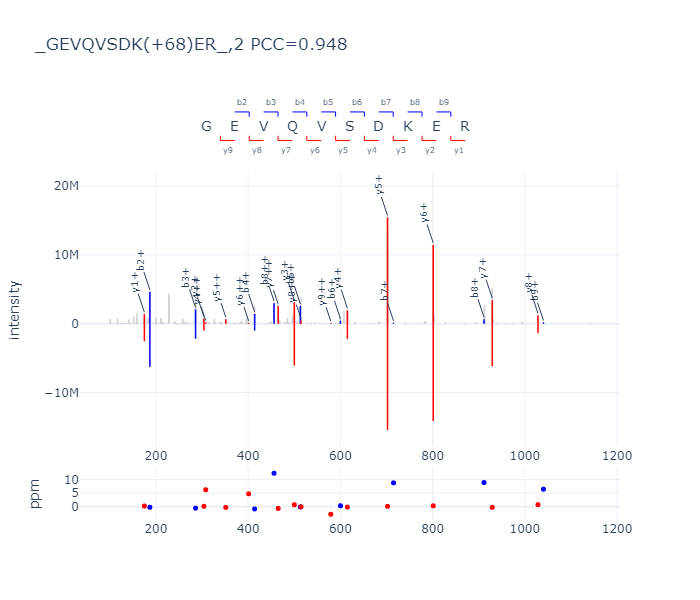

Supplement: Supplementary file 6 — Supplementary Data 3 [file 41467_2022_34904_MOESM6_ESM.zip › mirror-ms2-21ptm/Kmod_Crotonyl/_GEVQVSDK(+68)ER_charge=2_nce=35_transfer_pcc=0.95.png]

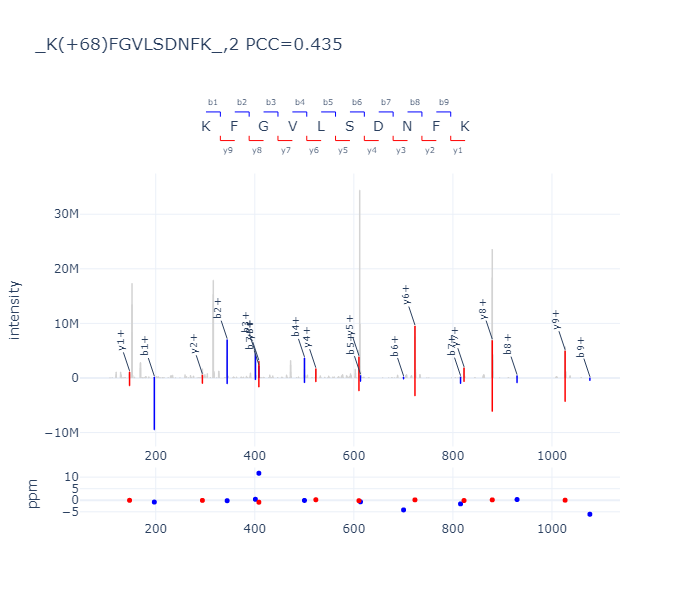

Supplement: Supplementary file 6 — Supplementary Data 3 [file 41467_2022_34904_MOESM6_ESM.zip › mirror-ms2-21ptm/Kmod_Crotonyl/_K(+68)FGVLSDNFK_charge=2_nce=30_pretrain_pcc=0.43.png]

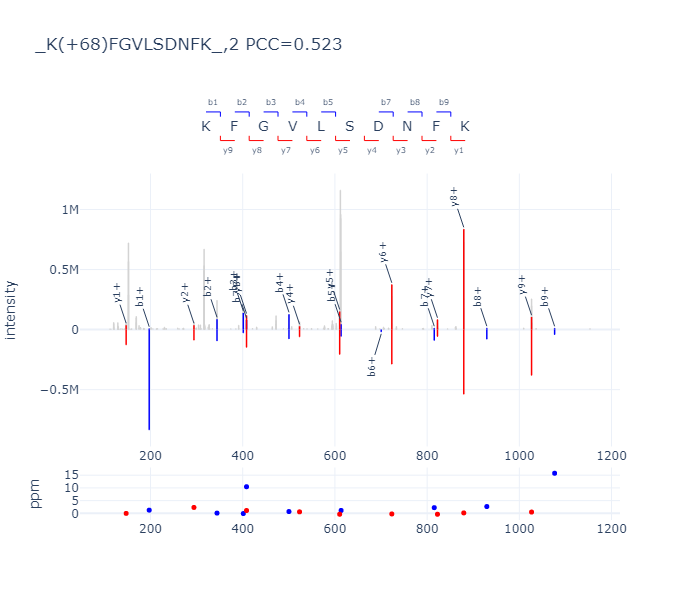

Supplement: Supplementary file 6 — Supplementary Data 3 [file 41467_2022_34904_MOESM6_ESM.zip › mirror-ms2-21ptm/Kmod_Crotonyl/_K(+68)FGVLSDNFK_charge=2_nce=30_pretrain_pcc=0.52.png]

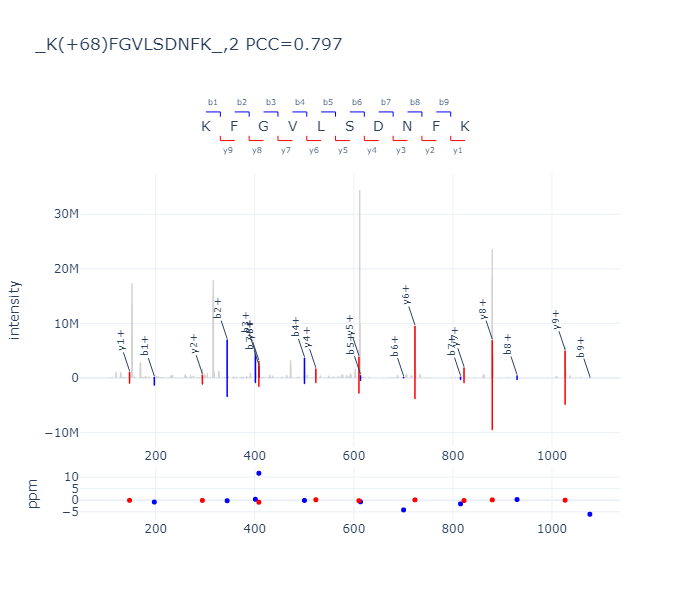

Supplement: Supplementary file 6 — Supplementary Data 3 [file 41467_2022_34904_MOESM6_ESM.zip › mirror-ms2-21ptm/Kmod_Crotonyl/_K(+68)FGVLSDNFK_charge=2_nce=30_transfer_pcc=0.80.png]

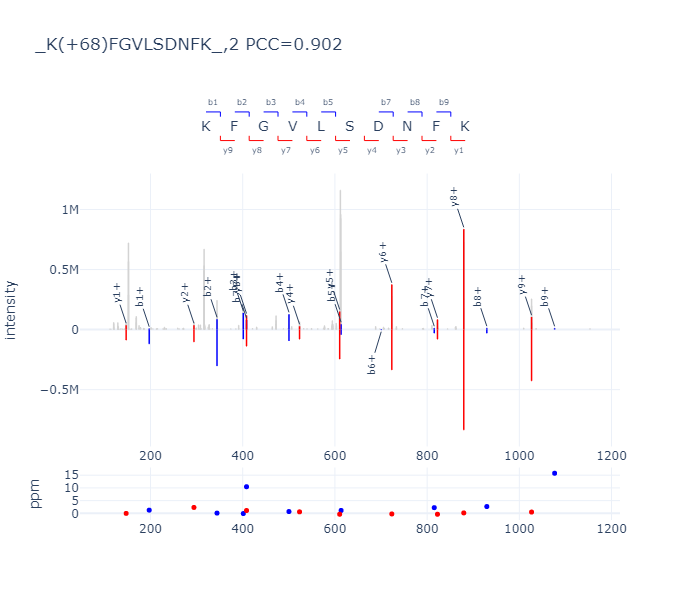

Supplement: Supplementary file 6 — Supplementary Data 3 [file 41467_2022_34904_MOESM6_ESM.zip › mirror-ms2-21ptm/Kmod_Crotonyl/_K(+68)FGVLSDNFK_charge=2_nce=30_transfer_pcc=0.90.png]

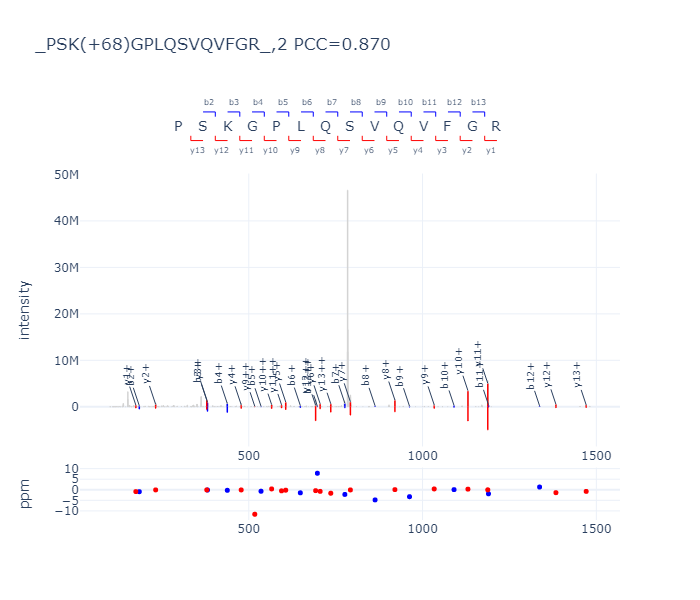

Supplement: Supplementary file 6 — Supplementary Data 3 [file 41467_2022_34904_MOESM6_ESM.zip › mirror-ms2-21ptm/Kmod_Crotonyl/_PSK(+68)GPLQSVQVFGR_charge=2_nce=30_pretrain_pcc=0.87.png]

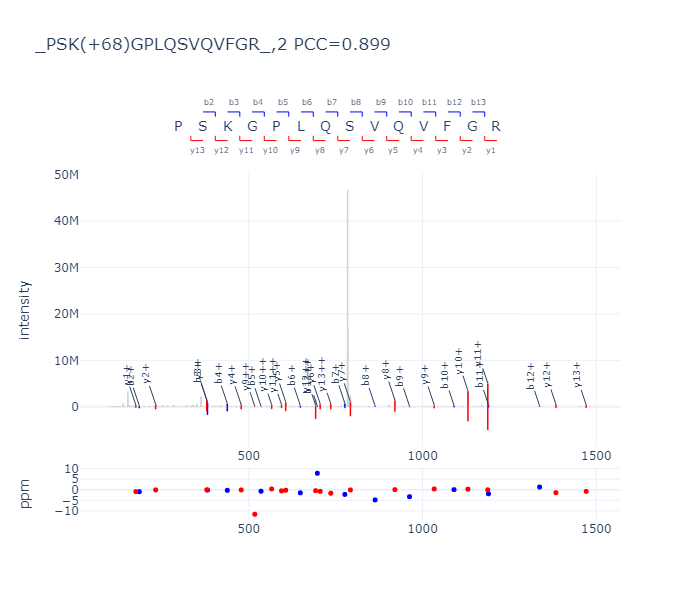

Supplement: Supplementary file 6 — Supplementary Data 3 [file 41467_2022_34904_MOESM6_ESM.zip › mirror-ms2-21ptm/Kmod_Crotonyl/_PSK(+68)GPLQSVQVFGR_charge=2_nce=30_transfer_pcc=0.90.png]

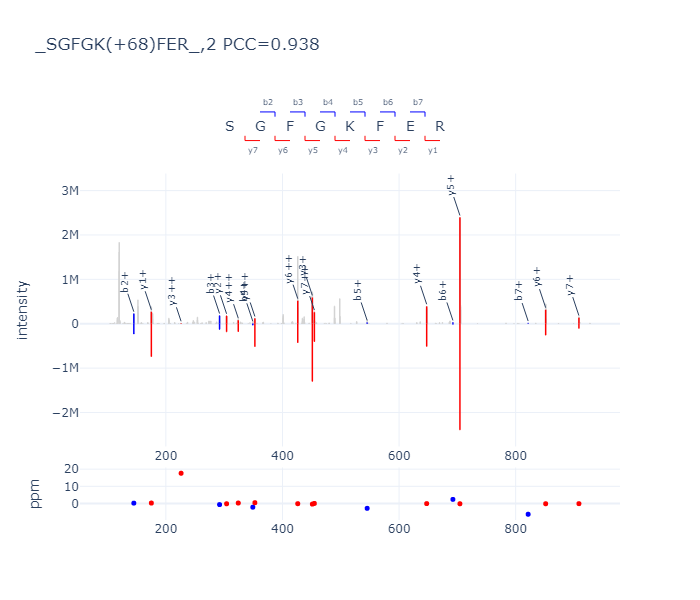

Supplement: Supplementary file 6 — Supplementary Data 3 [file 41467_2022_34904_MOESM6_ESM.zip › mirror-ms2-21ptm/Kmod_Crotonyl/_SGFGK(+68)FER_charge=2_nce=35_pretrain_pcc=0.94.png]

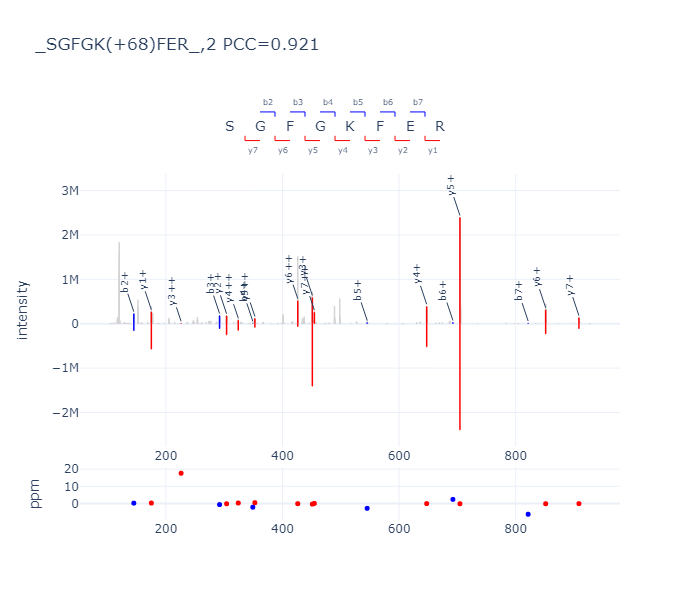

Supplement: Supplementary file 6 — Supplementary Data 3 [file 41467_2022_34904_MOESM6_ESM.zip › mirror-ms2-21ptm/Kmod_Crotonyl/_SGFGK(+68)FER_charge=2_nce=35_transfer_pcc=0.92.png]

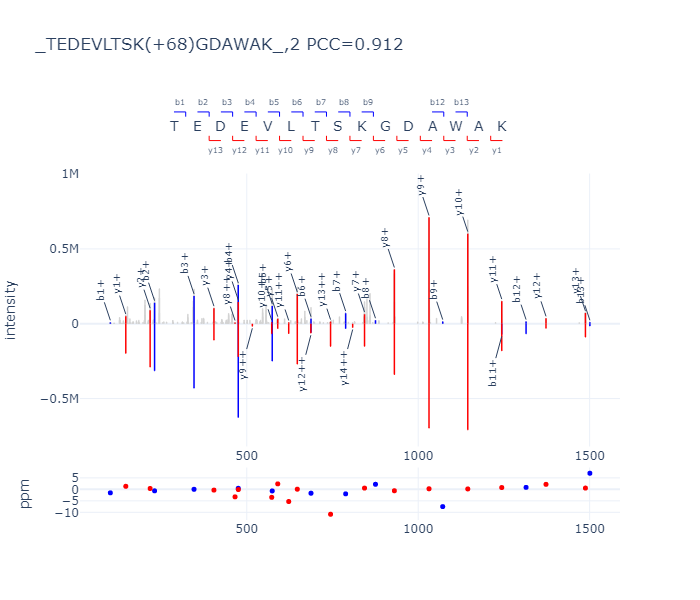

Supplement: Supplementary file 6 — Supplementary Data 3 [file 41467_2022_34904_MOESM6_ESM.zip › mirror-ms2-21ptm/Kmod_Crotonyl/_TEDEVLTSK(+68)GDAWAK_charge=2_nce=30_pretrain_pcc=0.91.png]

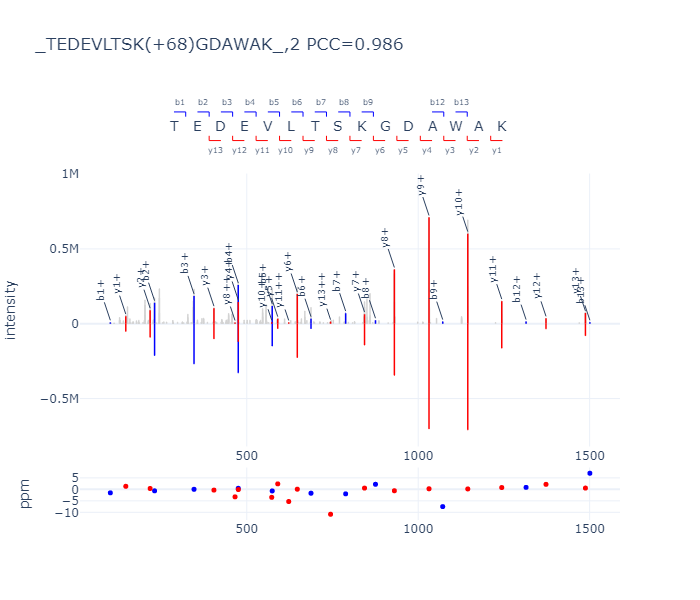

Supplement: Supplementary file 6 — Supplementary Data 3 [file 41467_2022_34904_MOESM6_ESM.zip › mirror-ms2-21ptm/Kmod_Crotonyl/_TEDEVLTSK(+68)GDAWAK_charge=2_nce=30_transfer_pcc=0.99.png]

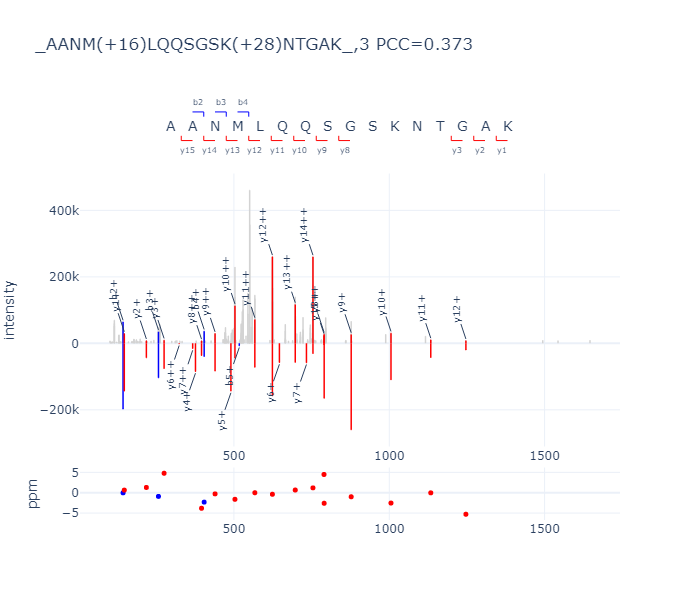

Supplement: Supplementary file 6 — Supplementary Data 3 [file 41467_2022_34904_MOESM6_ESM.zip › mirror-ms2-21ptm/Kmod_Dimethyl/_AANM(+16)LQQSGSK(+28)NTGAK_charge=3_nce=35_pretrain_pcc=0.37.png]

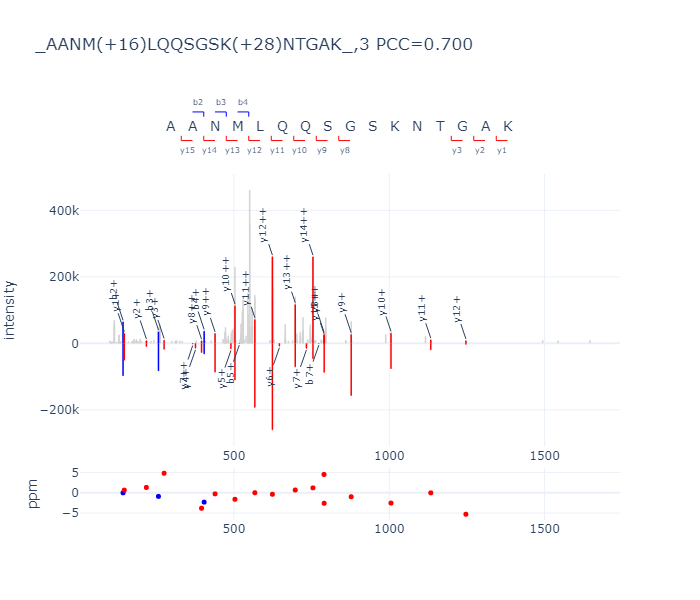

Supplement: Supplementary file 6 — Supplementary Data 3 [file 41467_2022_34904_MOESM6_ESM.zip › mirror-ms2-21ptm/Kmod_Dimethyl/_AANM(+16)LQQSGSK(+28)NTGAK_charge=3_nce=35_transfer_pcc=0.70.png]

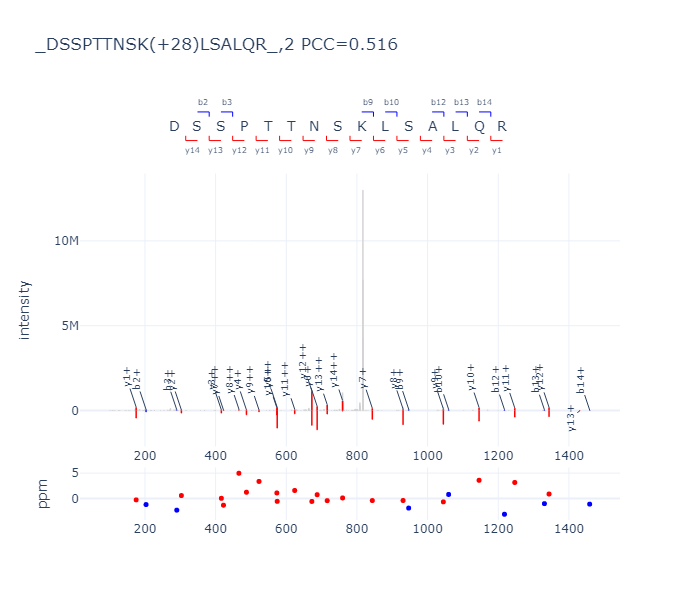

Supplement: Supplementary file 6 — Supplementary Data 3 [file 41467_2022_34904_MOESM6_ESM.zip › mirror-ms2-21ptm/Kmod_Dimethyl/_DSSPTTNSK(+28)LSALQR_charge=2_nce=35_pretrain_pcc=0.52.png]

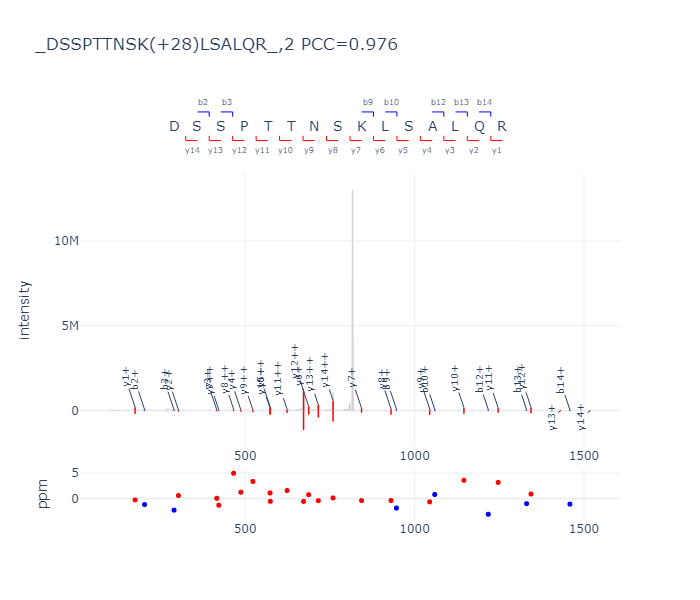

Supplement: Supplementary file 6 — Supplementary Data 3 [file 41467_2022_34904_MOESM6_ESM.zip › mirror-ms2-21ptm/Kmod_Dimethyl/_DSSPTTNSK(+28)LSALQR_charge=2_nce=35_transfer_pcc=0.98.png]

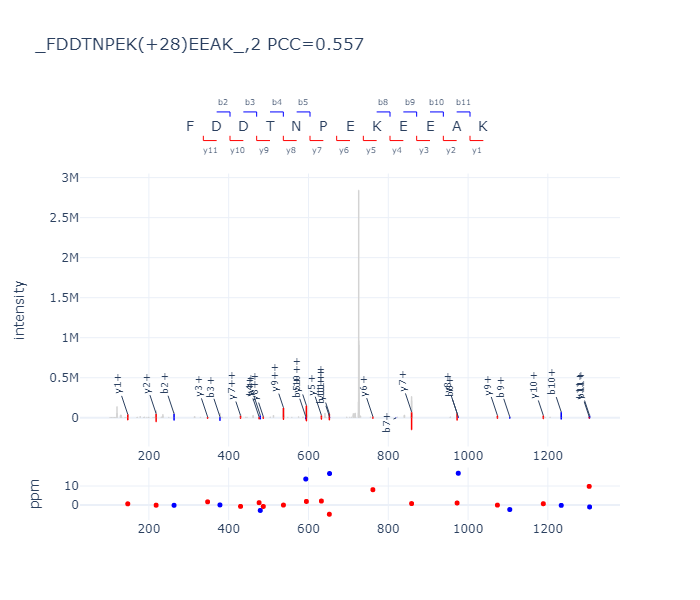

Supplement: Supplementary file 6 — Supplementary Data 3 [file 41467_2022_34904_MOESM6_ESM.zip › mirror-ms2-21ptm/Kmod_Dimethyl/_FDDTNPEK(+28)EEAK_charge=2_nce=30_pretrain_pcc=0.56.png]

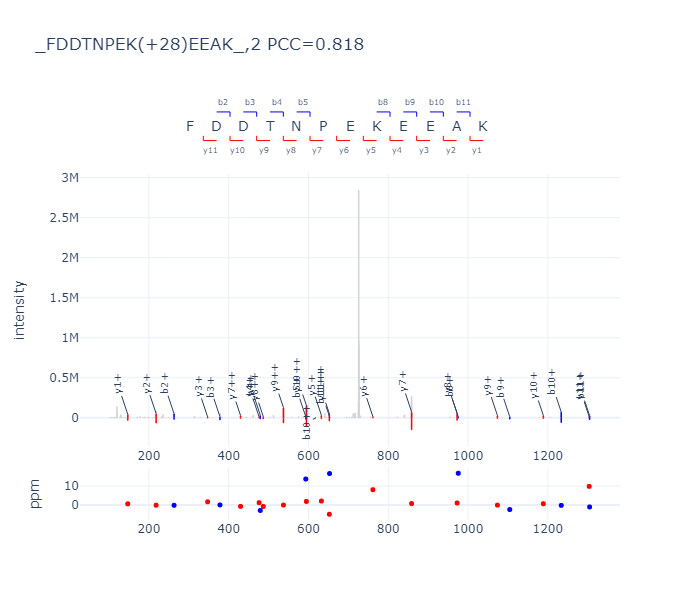

Supplement: Supplementary file 6 — Supplementary Data 3 [file 41467_2022_34904_MOESM6_ESM.zip › mirror-ms2-21ptm/Kmod_Dimethyl/_FDDTNPEK(+28)EEAK_charge=2_nce=30_transfer_pcc=0.82.png]

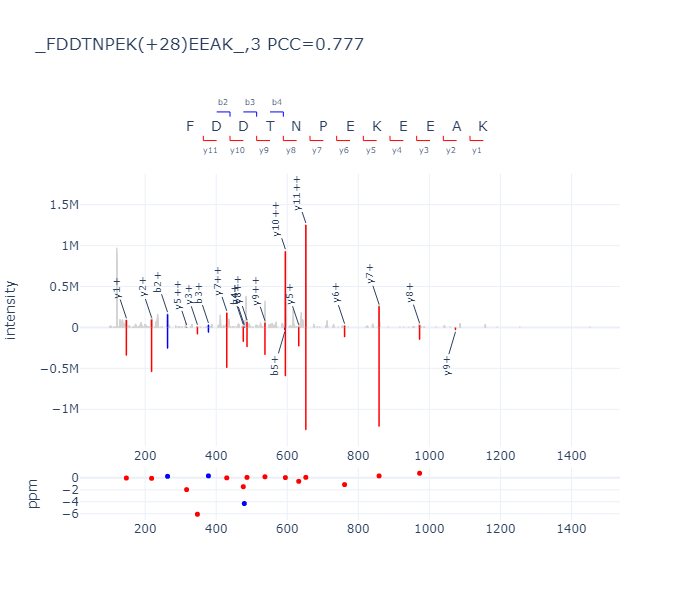

Supplement: Supplementary file 6 — Supplementary Data 3 [file 41467_2022_34904_MOESM6_ESM.zip › mirror-ms2-21ptm/Kmod_Dimethyl/_FDDTNPEK(+28)EEAK_charge=3_nce=30_pretrain_pcc=0.78.png]

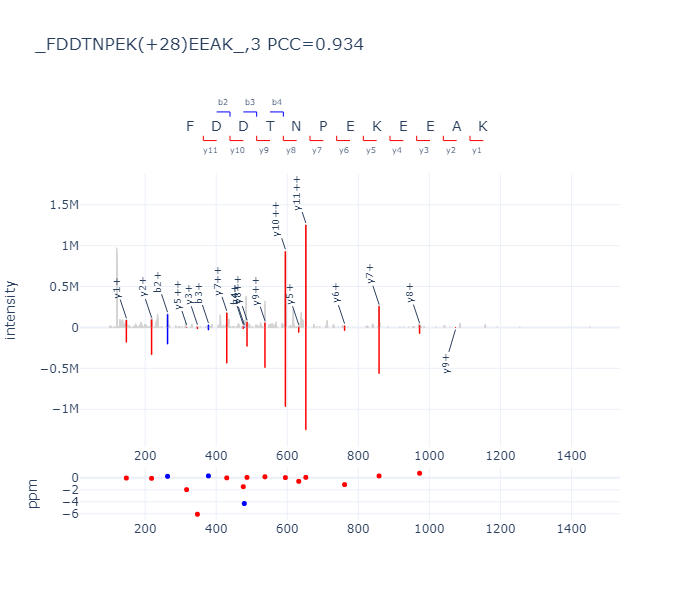

Supplement: Supplementary file 6 — Supplementary Data 3 [file 41467_2022_34904_MOESM6_ESM.zip › mirror-ms2-21ptm/Kmod_Dimethyl/_FDDTNPEK(+28)EEAK_charge=3_nce=30_transfer_pcc=0.93.png]

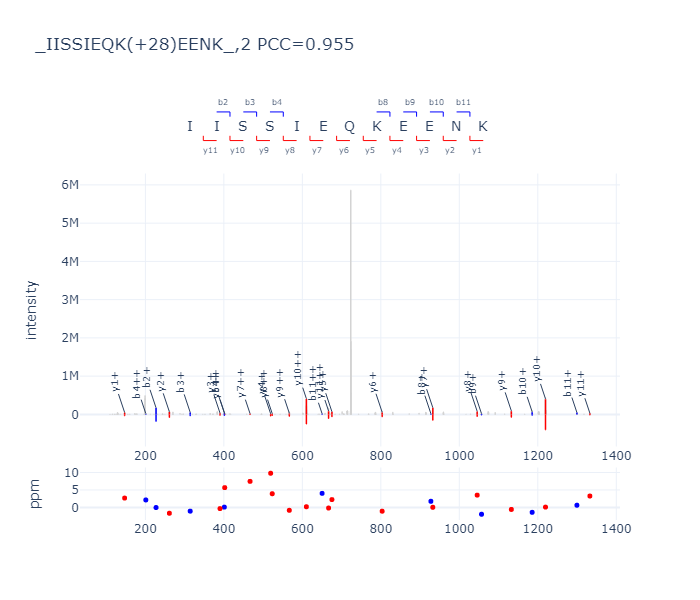

Supplement: Supplementary file 6 — Supplementary Data 3 [file 41467_2022_34904_MOESM6_ESM.zip › mirror-ms2-21ptm/Kmod_Dimethyl/_IISSIEQK(+28)EENK_charge=2_nce=30_pretrain_pcc=0.95.png]

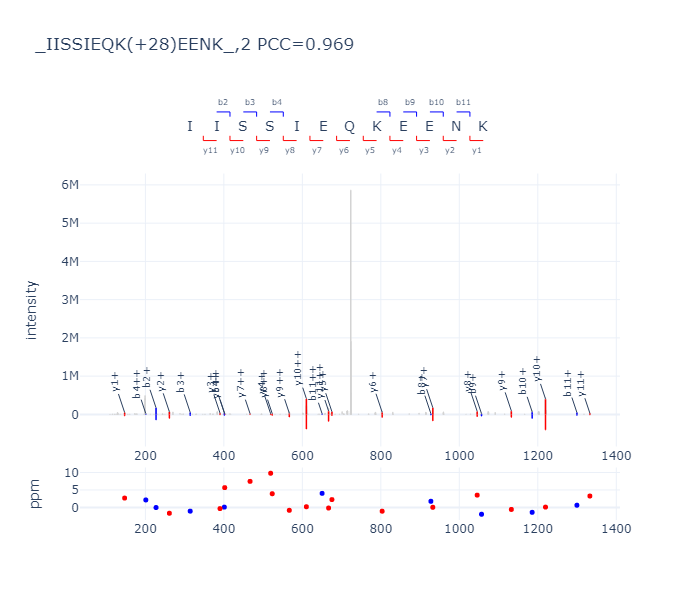

Supplement: Supplementary file 6 — Supplementary Data 3 [file 41467_2022_34904_MOESM6_ESM.zip › mirror-ms2-21ptm/Kmod_Dimethyl/_IISSIEQK(+28)EENK_charge=2_nce=30_transfer_pcc=0.97.png]

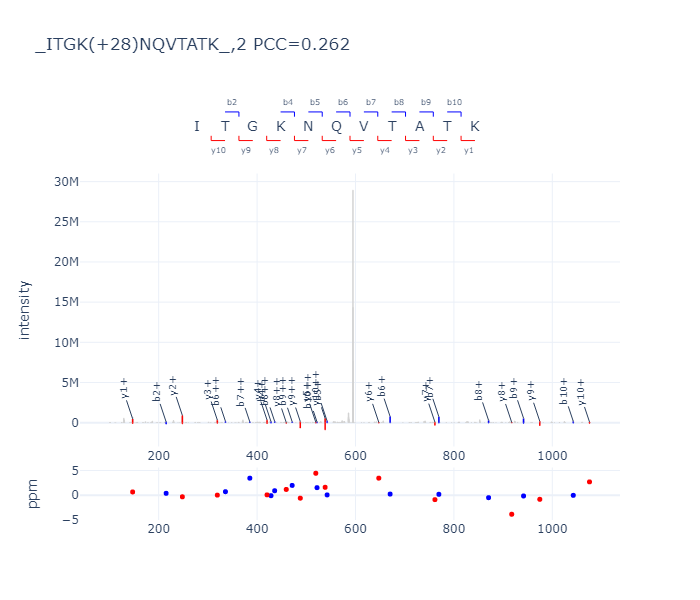

Supplement: Supplementary file 6 — Supplementary Data 3 [file 41467_2022_34904_MOESM6_ESM.zip › mirror-ms2-21ptm/Kmod_Dimethyl/_ITGK(+28)NQVTATK_charge=2_nce=25_pretrain_pcc=0.26.png]

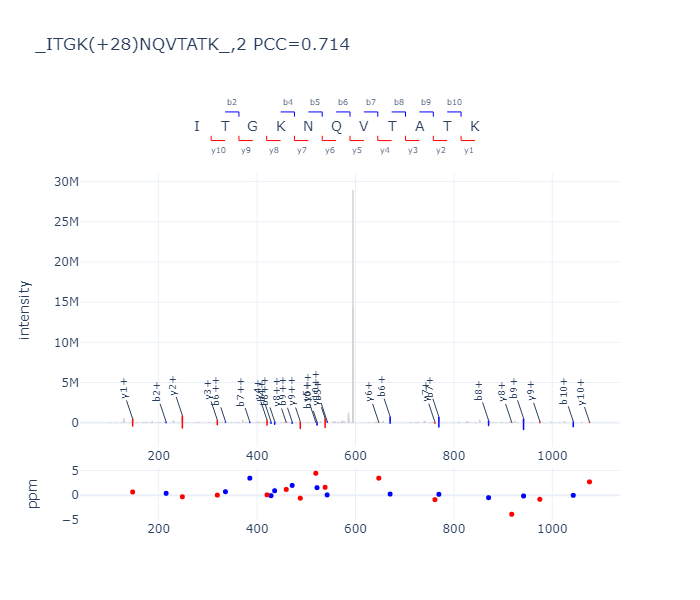

Supplement: Supplementary file 6 — Supplementary Data 3 [file 41467_2022_34904_MOESM6_ESM.zip › mirror-ms2-21ptm/Kmod_Dimethyl/_ITGK(+28)NQVTATK_charge=2_nce=25_transfer_pcc=0.71.png]

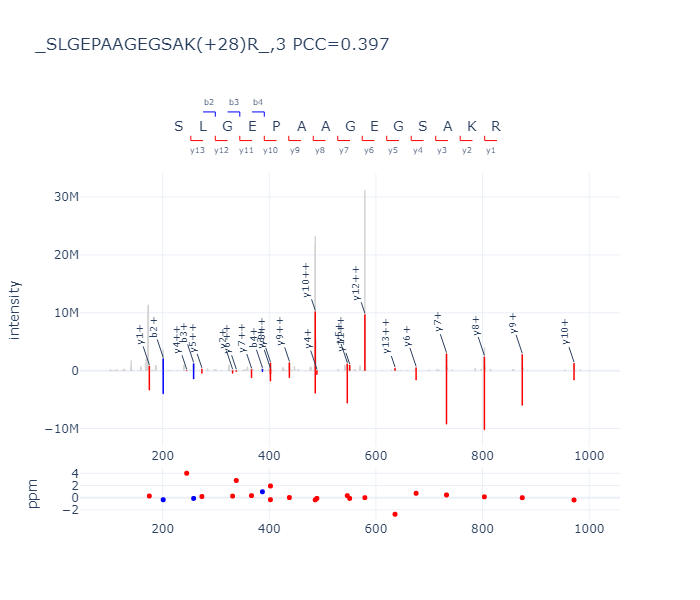

Supplement: Supplementary file 6 — Supplementary Data 3 [file 41467_2022_34904_MOESM6_ESM.zip › mirror-ms2-21ptm/Kmod_Dimethyl/_SLGEPAAGEGSAK(+28)R_charge=3_nce=35_pretrain_pcc=0.40.png]

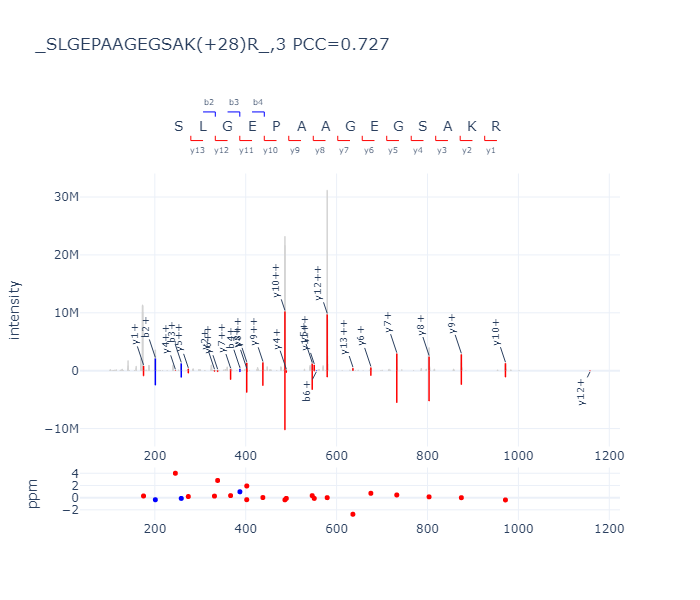

Supplement: Supplementary file 6 — Supplementary Data 3 [file 41467_2022_34904_MOESM6_ESM.zip › mirror-ms2-21ptm/Kmod_Dimethyl/_SLGEPAAGEGSAK(+28)R_charge=3_nce=35_transfer_pcc=0.73.png]

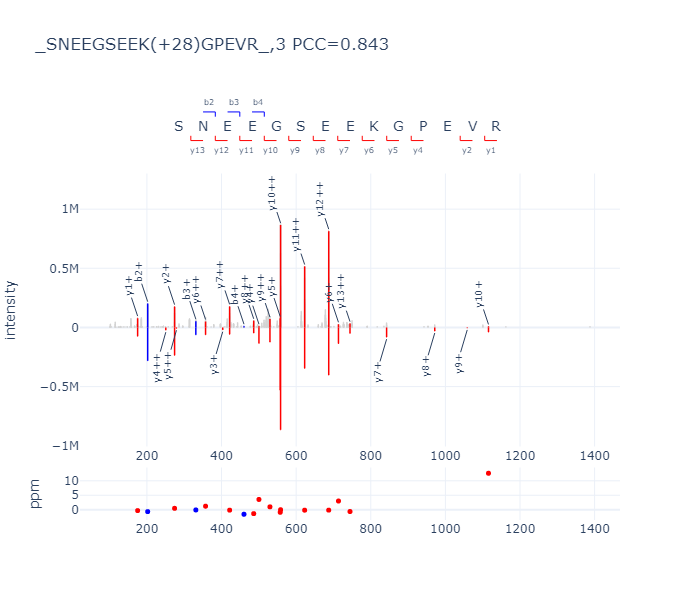

Supplement: Supplementary file 6 — Supplementary Data 3 [file 41467_2022_34904_MOESM6_ESM.zip › mirror-ms2-21ptm/Kmod_Dimethyl/_SNEEGSEEK(+28)GPEVR_charge=3_nce=30_pretrain_pcc=0.84.png]

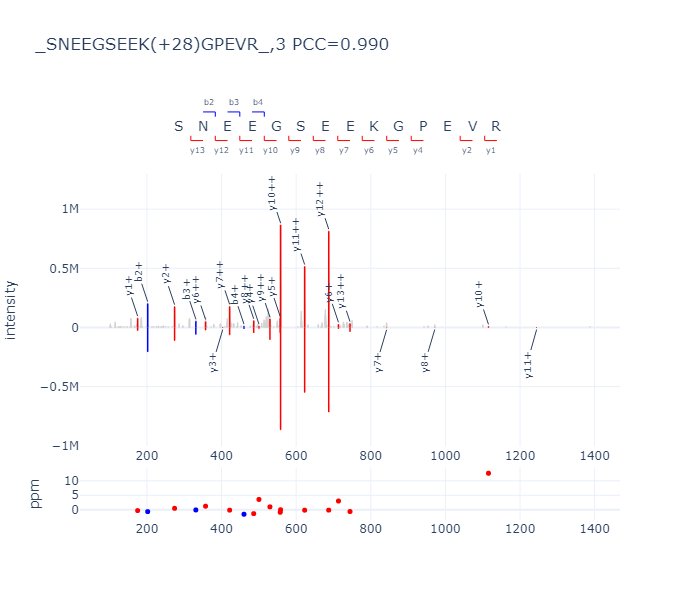

Supplement: Supplementary file 6 — Supplementary Data 3 [file 41467_2022_34904_MOESM6_ESM.zip › mirror-ms2-21ptm/Kmod_Dimethyl/_SNEEGSEEK(+28)GPEVR_charge=3_nce=30_transfer_pcc=0.99.png]

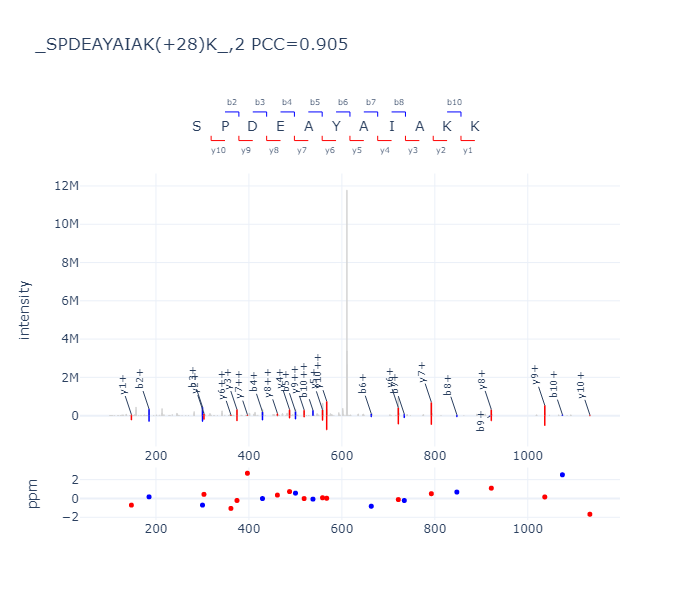

Supplement: Supplementary file 6 — Supplementary Data 3 [file 41467_2022_34904_MOESM6_ESM.zip › mirror-ms2-21ptm/Kmod_Dimethyl/_SPDEAYAIAK(+28)K_charge=2_nce=30_pretrain_pcc=0.91.png]

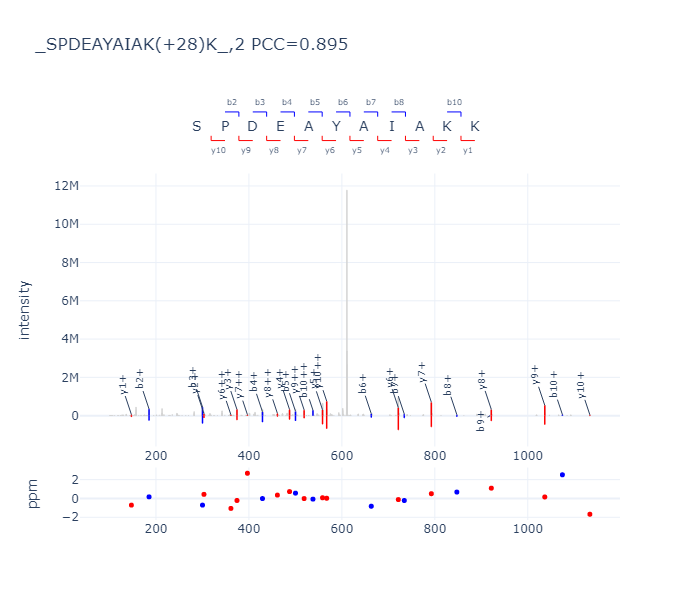

Supplement: Supplementary file 6 — Supplementary Data 3 [file 41467_2022_34904_MOESM6_ESM.zip › mirror-ms2-21ptm/Kmod_Dimethyl/_SPDEAYAIAK(+28)K_charge=2_nce=30_transfer_pcc=0.89.png]

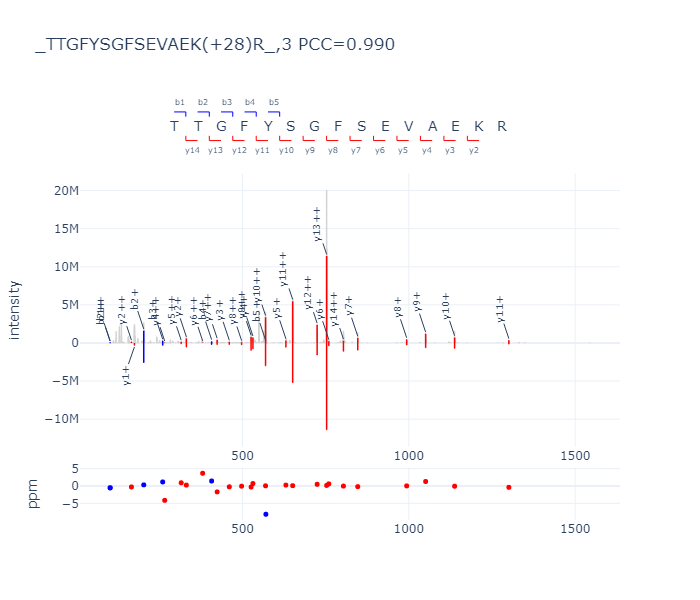

Supplement: Supplementary file 6 — Supplementary Data 3 [file 41467_2022_34904_MOESM6_ESM.zip › mirror-ms2-21ptm/Kmod_Dimethyl/_TTGFYSGFSEVAEK(+28)R_charge=3_nce=25_pretrain_pcc=0.99.png]

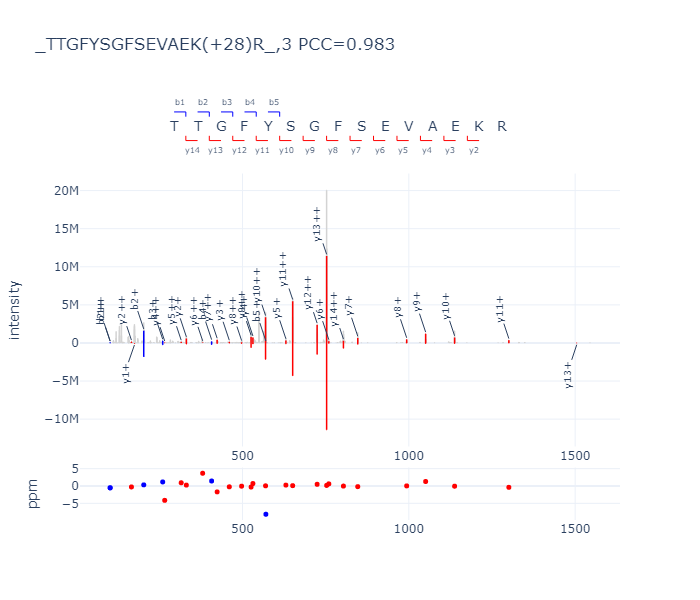

Supplement: Supplementary file 6 — Supplementary Data 3 [file 41467_2022_34904_MOESM6_ESM.zip › mirror-ms2-21ptm/Kmod_Dimethyl/_TTGFYSGFSEVAEK(+28)R_charge=3_nce=25_transfer_pcc=0.98.png]

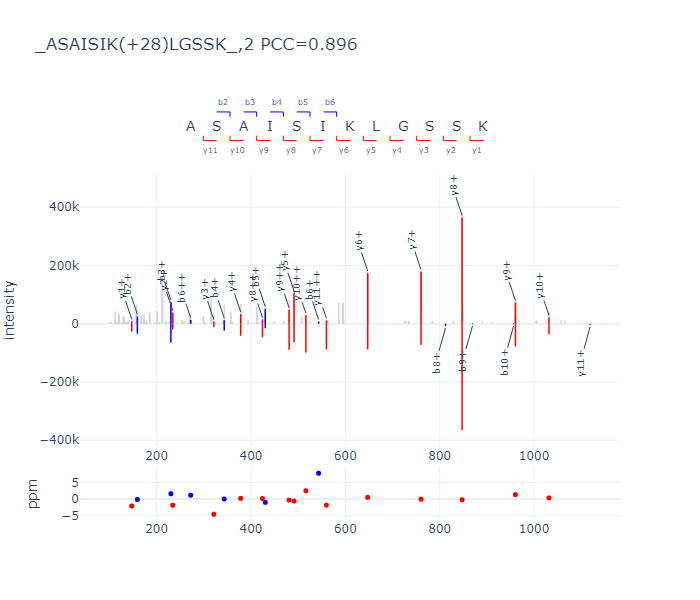

Supplement: Supplementary file 6 — Supplementary Data 3 [file 41467_2022_34904_MOESM6_ESM.zip › mirror-ms2-21ptm/Kmod_Formyl/_ASAISIK(+28)LGSSK_charge=2_nce=30_pretrain_pcc=0.90.png]
